# Intracranial Compliance Concepts and Assessment: A Scoping Review

**DOI:** 10.3389/fneur.2021.756112

**Published:** 2021-10-25

**Authors:** Gabriela Nagai Ocamoto, Thiago Luiz Russo, Rafaella Mendes Zambetta, Gustavo Frigieri, Cintya Yukie Hayashi, Sérgio Brasil, Nicollas Nunes Rabelo, Deusdedit Lineu Spavieri Júnior

**Affiliations:** ^1^Federal University of São Carlos, São Carlos, Brazil; ^2^Braincare, São Carlos, Brazil; ^3^Department of Neurology, Faculty of Medicine, University of São Paulo, São Paulo, Brazil; ^4^Department of Neurosurgery, Atenas School of Medicine, Passos, Brazil

**Keywords:** mapping review, brain compliance, intracranial elastance, brain pulsatility, intracranial compliance

## Abstract

**Background:** Intracranial compliance (ICC) has been studied to complement the interpretation of intracranial pressure (ICP) in neurocritical care and help predict brain function deterioration. It has been reported that ICC is related to maintaining ICP stability despite changes in intracranial volume. However, this has not been properly translated to clinical practice. Therefore, the main objective of this scoping review was to map the key concepts of ICC in the literature. This review also aimed to characterize the relationship between ICC and ICP and systematically describe the outcomes used to assess ICC using both invasive and non-invasive measurement methods.

**Methods:** This review included the following: (1) population: animal and humans, (2) concept of compliance or its inverse “elastance,” and (3) context: neurocritical care. Therefore, literature searches without a time frame were conducted on several databases using a combination of keywords and descriptors.

**Results and Discussion:** 43,339 articles were identified, and 297 studies fulfilled the inclusion criteria after the selection process. One hundred and five studies defined ICC. The concept was organized into three main components: physiological definition, clinical interpretation, and localization of the phenomena. Most of the studies reported the concept of compliance related to variations in volume and pressure or its inverse (elastance), primarily in the intracranial compartment. In addition, terms like “accommodation,” “compensation,” “reserve capacity,” and “buffering ability” were used to describe the clinical interpretation. The second part of this review describes the techniques (invasive and non-invasive) and outcomes used to measure ICC. A total of 297 studies were included. The most common method used was invasive, representing 57–88% of the studies. The most commonly assessed variables were related to ICP, especially the absolute values or pulse amplitude. ICP waveforms should be better explored, along with the potential of non-invasive methods once the different aspects of ICC can be measured.

**Conclusion:** ICC monitoring could be considered a complementary resource for ICP monitoring and clinical examination. The combination and validation of invasive/non-invasive or non-invasive measurement methods are required.

## Background

The “consensus summary statement of the International Multidisciplinary Consensus Conference on Multimodality Monitoring in Neurocritical Care” has provided a key rationale for neurocritical care units around the world (1). Multimodality monitoring of patients with acute neurological disorders is a complementary approach to frequent bedside examinations. It should be considered for the detection of early neurological worsening before irreversible brain damage occurs and to help clinicians guide individualized therapeutic decisions, which can improve, for example, acute and long-term prognosis (2).

In this sense, monitoring intracranial pressure (ICP) is fundamental to the care of patients with acute brain injury. In the literature, there is a strong recommendation with moderate quality evidence that ICP and cerebral perfusion pressure should be used as part of protocol-driven care in patients who are at risk of elevated ICP based on clinical and/or imaging features (1). The fundamental principles of raised ICP are condensed in the Monro-Kellie doctrine. The volume of the intracranial cavity is constant under normal conditions, and the maintenance of a steady ICP depends on the volume of the intracranial contents (brain tissue, blood, and cerebrospinal fluid) (3, 4).

Methods for ICP monitoring include both invasive and non-invasive approaches. Regarding invasive methods, the external ventricular drain is considered the gold standard for ICP monitoring (1). A study with a large cohort recently indicated the significant impact of ICP monitoring on outcomes for patients with an acute brain injury, despite the considerable heterogeneity of therapy protocols among specialized centers (5). Before this study, the efficacy of treatments based on invasive ICP monitoring for improving outcomes such as mortality, impaired consciousness, and functional and psychological status after hospital discharge was questionable (6). This previous study consolidated the diagnostic practice and recommended the standardization of treatment in neurocritical care.

Nevertheless, intracranial compliance (ICC) or its inverse, intracranial elastance, has been studied for decades in an attempt to complement ICP interpretation and describe brain homeostasis more precisely. Such findings would help neurocritical care teams anticipate brain function deterioration more accurately (7, 8). ICC has been found to be related to the compensatory mechanisms used to maintain ICP stability (4). For example, the higher the compensation, the higher the compliance of intracranial content to adapt to the volume and pressure changes. However, ICC has not been properly translated to clinical practice, and there remains a critical gap in clinical neuroscience technology.

Czosnyka and Citerio (7) hypothesized that ICC is a non-linear function of the association of venous, cerebrospinal fluid, and arterial pools, with the influence of other vascular factors such as the regulation of cerebral blood flow, tension of arterial smooth muscles, partial pressure of CO_2_, endothelial function, brain hydration, and metabolism.

Comprehensive reviews are available on ICP monitoring in neurocritical care (4, 9), which are focused on new technological approaches and gaps in research that can be translated into clinical practice. Still, there remains no standardization of ICC concepts according to invasive and non-invasive techniques.

This scoping review aims to map the key ICC concepts used in the literature. Second, this review characterizes the relationship between ICC and ICP and systematically describes the outcomes used to assess ICC, considering both invasive and non-invasive methods. Third, this review provides information about gaps in the body of knowledge to support future studies.

## Methods/Design

The scoping review was conducted according to the recommendations of the Joanna Briggs Institute (8, 10). Although scoping review protocols are not eligible for registration in the International Prospective Register of Systematic Review (PROSPERO) database, the respective protocol was previously available at Research Square (https://assets.researchsquare.com/files/rs-43616/v1_stamped.pdf). PRISMA-ScR (Preferred Reporting Items for Systematic Reviews and Meta-Analysis Extension for Scoping Reviews), which was developed according to guidelines published by the EQUATOR (Enhancing the Quality and Transparency of Health Research) Network for the development of reporting guidelines, was followed (11).

## Search Strategy and Data Source

A primary limited search conducted on April 16, 2020, was performed independently by three investigators (TLR, GNO, and RMZ) on PubMed (via the National Library of Medicine) and CINAHL databases to analyze the titles, abstracts, and index terms used. The combination of “intracranial compliance” OR “intracranial elastance” OR “cerebral compliance” OR “brain compliance” OR “compliance monitoring” AND “intracranial pressure” OR “intracranial pulse amplitude” OR “intracranial pulse wave” OR “pressure-volume curve” was used for this first search. Then, the MeSH database was consulted to identify and confirm the appropriate terms obtained in this first search, which were included in the second search strategy.

Subsequently, in the second search conducted on August 6, 2020, and updated on May 14, 2021, all the identified keywords and index terms described in the first step were used across PubMed (via the National Library of Medicine), CINAHL with Full Text (EBSCO), Web of Science (Thomson Scientific/ISI Web Services), and EMBASE databases. In addition, a search was conducted at Epistemonikos, Gray Literature Report, Clinical Trial Register, and Cochrane Clinical Trials. To follow the Clinical Trials Register search template, the search strategy was adapted to intracranial compliance, brain compliance, cerebral compliance, cerebrospinal compliance, intracranial elastance, brain elastance, cerebral elastance, cerebrospinal elastance, intracranial pulsatility, brain pulsatility, cerebral pulsatility, cerebrospinal pulsatility, compensatory reserve, and compliance monitoring. Additional articles were included through the manual revision of references listed on the articles found.

## Selection Process

To systematize and organize the search and data extraction, the State of the Art through Systematic Review (StArt, Available from http://lapes.dc.ufscar.br/tools/start_tool) were used. Two reviewers independently performed the selection process based on the eligibility criteria. Initially, duplicates were excluded using the StArt. Then the potentially eligible articles were selected based on their titles and abstracts. Afterward, these articles were retrieved for full text reading to verify whether they met all the inclusion criteria. In cases of disagreements between the two reviewers, a third reviewer was consulted to determine the study's eligibility.

## Eligibility Criteria

The main question of this study was based on *population, concept*, and *context* criteria. Studies were selected for inclusion if they met the following criteria: (1) animals or humans with no restriction of age; (2) conceptualization of compliance or its inverse “elastance;” and (3) health conditions related to the neurocritical care context.

As a secondary question, the ICC-related outcomes using invasive or non-invasive methods, described among the full read studies, were used to map the most common variables considered by the authors to assess ICC.

Experimental and epidemiological studies, including randomized controlled trials, non-randomized controlled trials, quasi-experimental studies, before and after studies, prospective and retrospective cohort studies, case-control studies, and analytical cross-sectional studies, were included in the analysis. In addition, literature reviews such as narrative reviews, meta-analyses, systematic reviews, gray literature (e.g., governmental reports, theses), and theoretical and opinion articles were considered. On the other hand, conference abstracts, personal blogs, and social media were excluded.

For feasibility reasons, articles in English, Spanish, Italian, Portuguese, and German were considered, with no limitation to the year of publication or country of origin.

## Data Analysis

### Data Extraction

A standardized electronic data extraction form built in Microsoft Excel was used to obtain key information about the studies included in this review. The data extracted from each primary study were as follows: authors, year of publication, type of study/research design, health condition/model, methods used for ICC assessment (invasive or non-invasive), and outcomes considered as ICC by the authors.

## Data Synthesis

The extracted data were condensed and displayed according to similarities and differences under the main conceptual categories. Three main categories were used to characterize the ICC concept: physiological definition, clinical interpretation, and anatomical localization. The results are presented as a map of the data extracted using descriptive analyses, frequency tables, and graphics.

## Results

**Figure 1:**
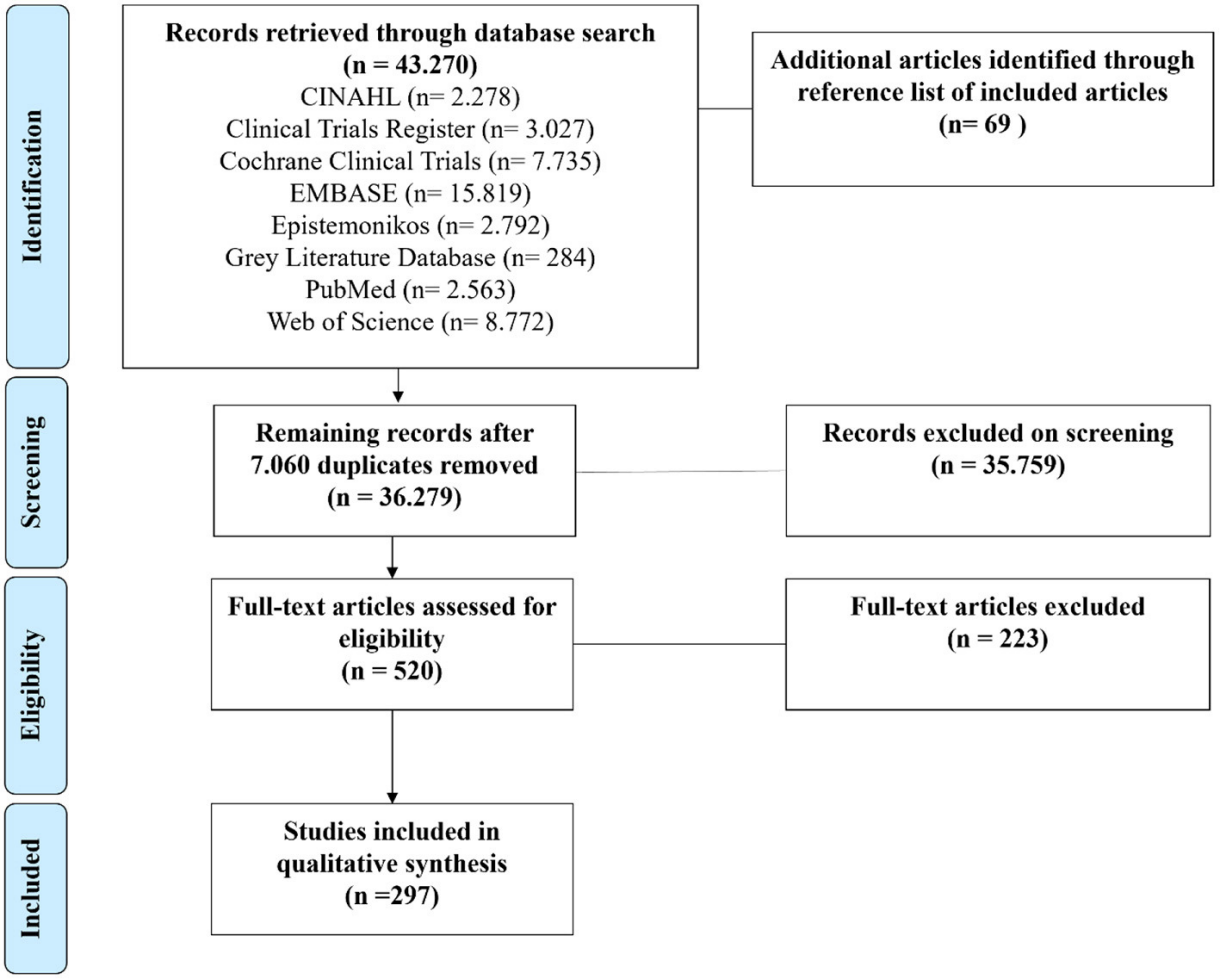
Study flow diagram.

A total of 43,339 articles were identified through the databases (CINAHL = 2,278, Clinical Trials Register = 3,027, Cochrane Clinical Trials = 7,735, EMBASE = 15,819, Epistemonikos = 2,792, gray literature database = 284, PubMed = 2,563, and Web of Science = 8,772), and 69 articles were identified through reviewing the reference lists of the included articles. After the selection process, 297 studies fulfilled the inclusion criteria (Figure 1).

**Figure 2:**
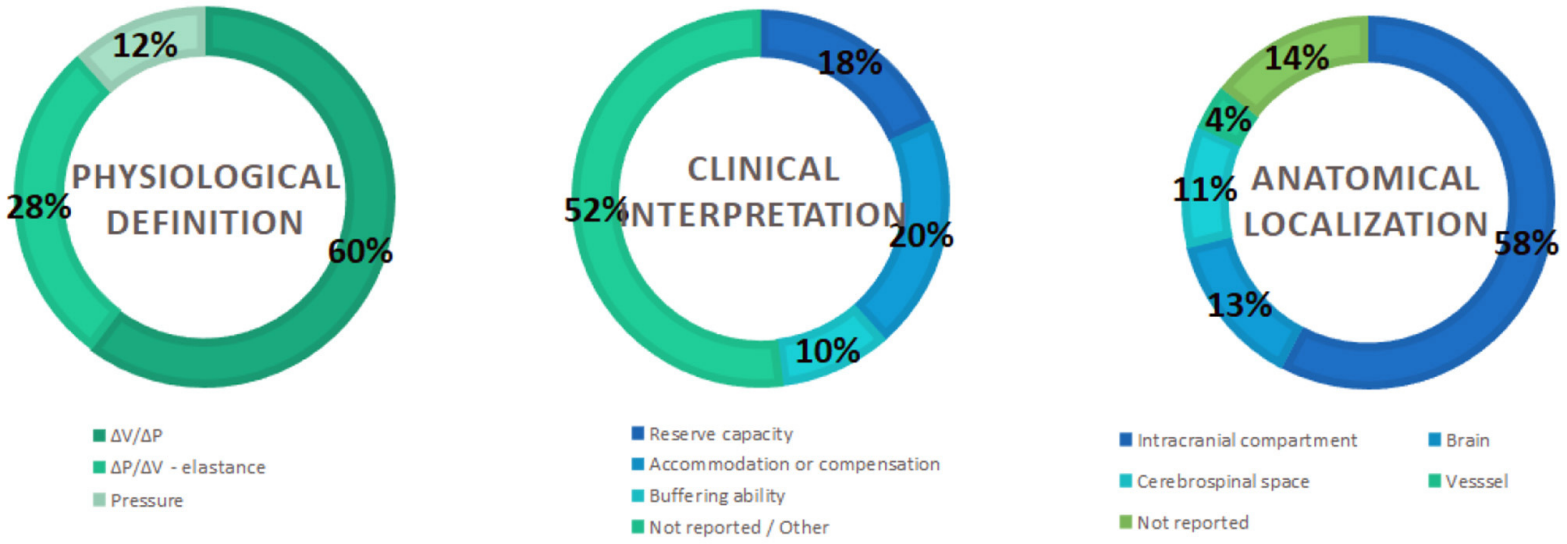
Percentage of mapped concepts.

Supplementary Table 1 shows the main concepts of ICC reported in the literature. The concept was organized into three main components: physiological definition, clinical interpretation, and localization of the phenomena. One hundred and five studies conceptualized ICC. As a physiological definition, 60% of the studies described ICC as the relationship between intracranial volume and pressure, while 28% described it as its inverse “elastance,” which is the ratio of ICP and volume. The other 12% of the studies correlated ICC with ICP variations. Concerning the clinical interpretation, 20% of the studies correlated ICC with accommodation or compensation, 18% as reserve capacity, and 10% as the buffering ability of the system, while 52% did not report ICC definition. Regarding anatomical localization, ICC was associated with the intracranial compartment in 58% of the studies, as well as with the brain (13%), cerebrospinal space (11%), and vessel compliance (4%). Fourteen percent did not report the association between ICC and anatomical localization. Figure 2 summarizes the percentage of the most frequent physiological definitions, clinical interpretations, and anatomical localization.

**Figure 3:**
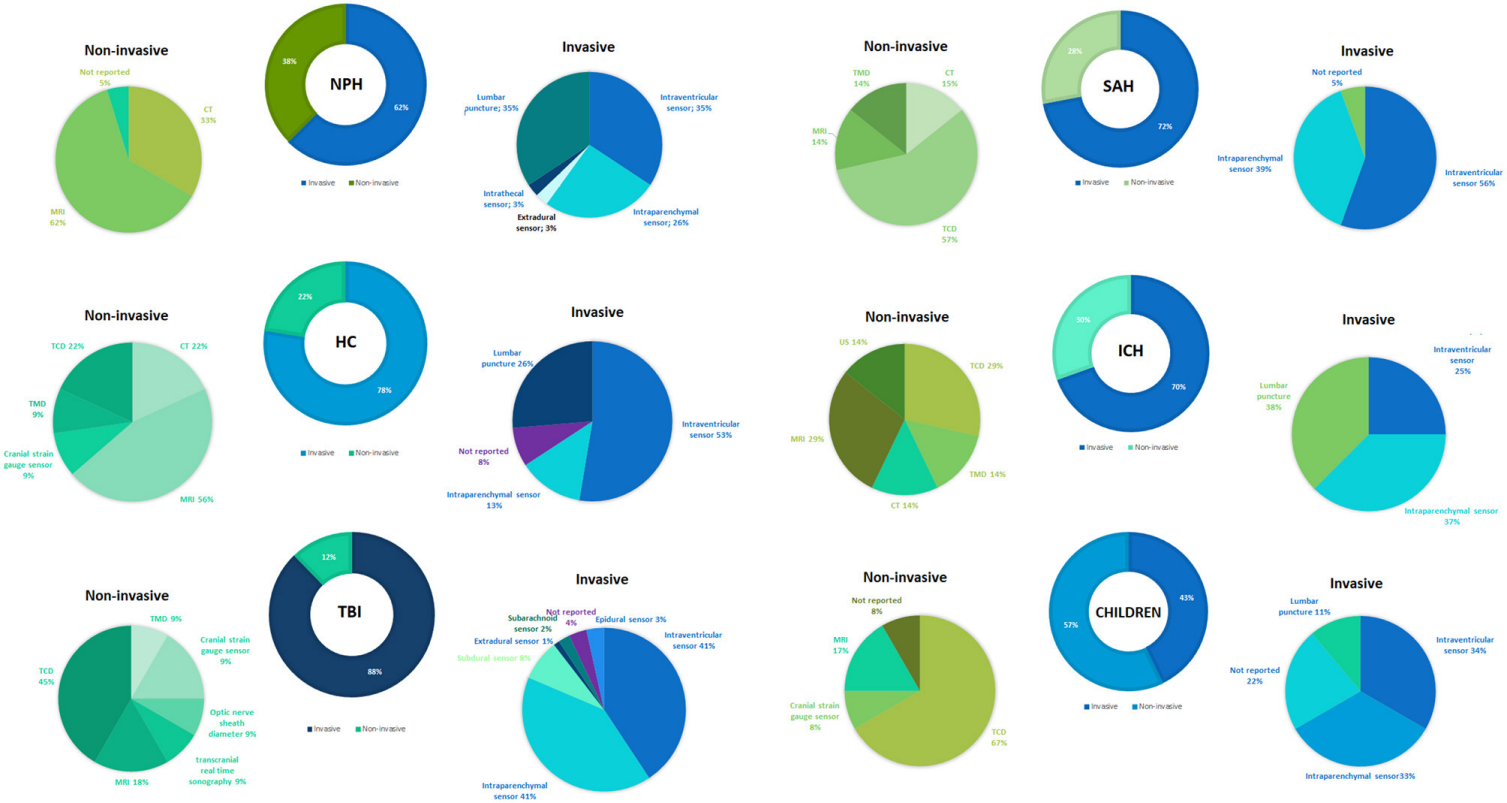
Most frequently reported methods and techniques for the assessment of intracranial compliance.

Supplementary Table 2 describes the methods (invasive or non-invasive), techniques, and outcomes used to measure ICC in the literature. Any study that suggested that the outcome could measure or infer ICC was considered to reflect the neurocritical care scenario. Therefore, 297 papers were included in this review. The most common diseases or health conditions related to neurocritical care scenarios and ICC in adults and the elderly were normal pressure hydrocephalus (NPH), hydrocephalus (HC), traumatic brain injury (TBI), subarachnoid hemorrhage (SAH), and intracranial hypertension (ICH), with invasive methods representing 57–88% of the studies included in this review (Figure 3).

Figure 3 presents an overview of the most common methods used for the invasive assessment of ICC. Invasive sensor placement (intraventricular, intraparenchymal, intrathecal, subarachnoid, epidural) and lumbar puncture were the most frequently reported invasive techniques. On the other hand, CT, MRI, TMD, TCD, cranial strain gauge sensor, optic nerve sheath diameter, transcranial real-time sonography, and ultrasonography (US) were used for the non-invasive assessment of ICC. Among children, the invasive method (intraventricular, intraparenchymal, and lumbar puncture) is the most frequently reported (62%). Furthermore, CT and MRI techniques were described for the non-invasive assessment of ICC (Figure 3).

**Figure 4:**
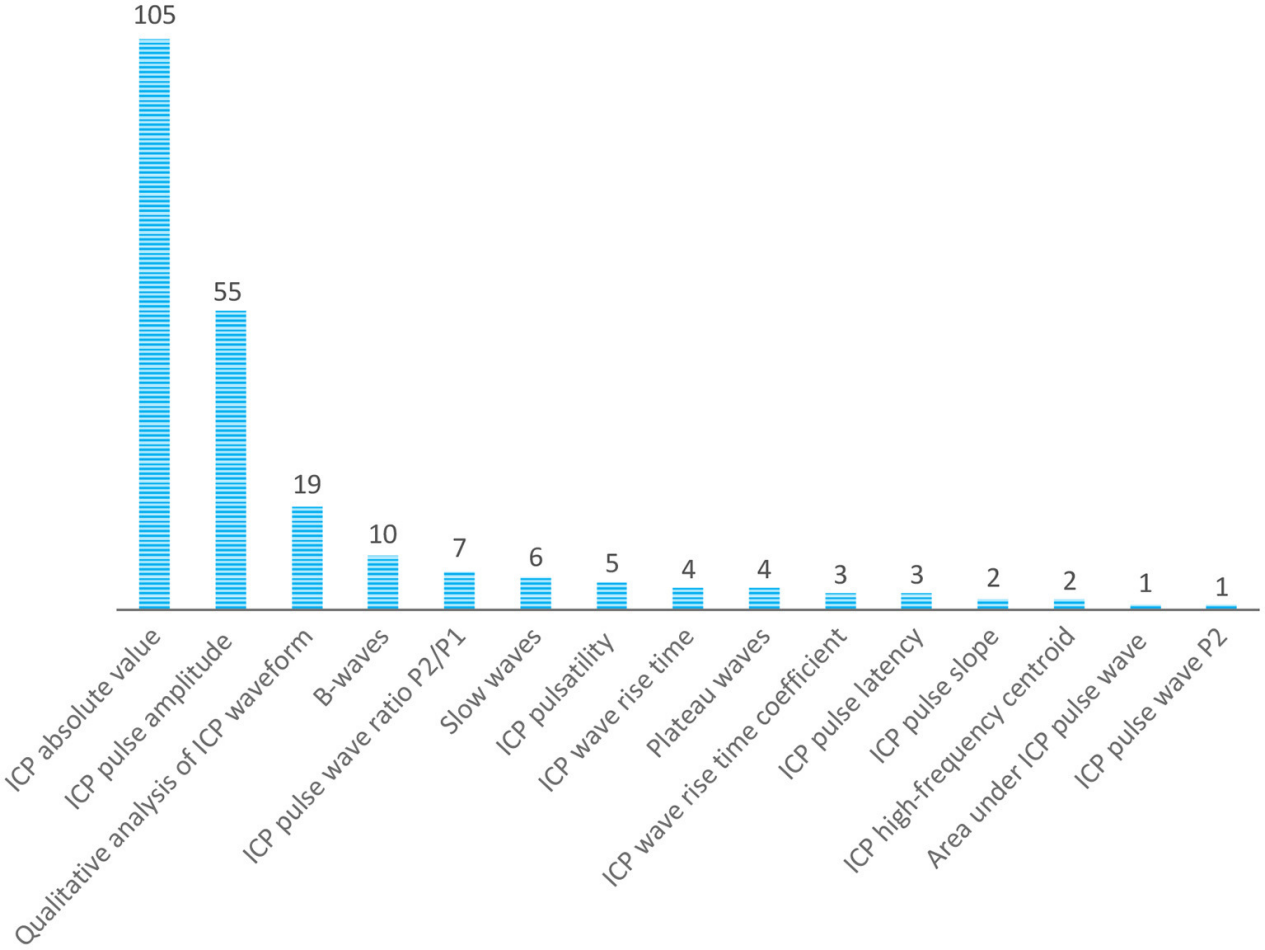
Overview of the most assessed variables related to ICP.

Figure 4 presents an overview of the most commonly assessed variables related to ICP. Most of the studies reported ICP absolute values or pulse amplitude. Variables derived from ICP waveform morphology are less frequently reported.

## Discussion

This review systematically mapped the concept of ICC in the context of neurocritical care. For decades, compliance has been defined as a volume pressure relationship, although elastance (pressure-volume relationship) has been extensively used for similar clinical applications. Studies usually reported compliance as the inverse of elastance and vice versa. In addition, clinical interpretation varied among the studies, but the understanding of the regulatory effect of intracranial content to support volume variations without changes in pressure is frequently attributed to ICC in invasive or non-invasive methods.

Invasive methods were most frequently reported for monitoring ICC. Moreover, ICP outcomes, such as the absolute value or pulse amplitude, were frequently used to assess and infer ICC. Although ICP waveform morphology has been less explored by researchers, the advances in data science in the last decade have brought new perspectives with which to explore ICP waveform morphology and establish more accurate relationships to ICC. In this sense, large, well-conducted multidisciplinary studies are necessary to explore and validate this association. In addition, open data can optimize the efforts for big data studies in ICP and ICC. It would also be helpful to compare different diseases and methods of assessment.

Likewise, complementary approaches besides ICP monitoring can be relevant guides to help with therapeutic decision making, improve survival, and patients' recovery after acute brain injury. In this sense, ICC monitoring could be considered a complementary resource for ICP monitoring and clinical examination. Although ICC gained much attention in the neurosurgical literature decades ago, it has rarely been used in clinical practice (12). With the advancement of data science and the interface between cardiovascular and central nervous system research, different methods are being used to directly or indirectly assess the intracranial volume pressure reserve capacity. It is recognized that ICP combines markers of cerebrovascular hemodynamics and ICC (13).

Considering the application of ICC in the context of disease, most clinical and experimental studies have used infusion tests to derive interest indices. This test is conducted by injecting saline and/or withdrawing CSF while the intracranial volume is controlled and observing the ICP response. Some important indices acquired from this test are CSF dynamics and resistance to outflow, compliance and elastance slope, PVR, VPR, and PVI. Nevertheless, many studies have attempted to predict ICC changes by assessing the characteristics of ICP, such as amplitude and waveform pulse, through long-term monitoring. Nevertheless, more studies are necessary to improve the sensitivity of predictions.

Regarding non-invasive technologies for ICC monitoring, some benefits can be achieved compared to invasive methods since invasive methods are expensive, require experienced neurosurgical teams, and present intrinsic risks such as infections and hemorrhage. In the present study, CT, RMN, TCD, TMD, cranial strain gauge sensors, optic nerve sheath diameter measurement, and transcranial sonography were used to assess ICC. However, the most valuable feature of these non-invasive techniques may be the possibility of assessment for patients at risk of ICC impairment development, with either contraindication or ethical hindrances for ICP catheter placement, such as liver failure (14), severe acute respiratory syndrome (15), or sepsis. Hence, a task force is necessary to better explore the potential of non-invasive methods and provide robust evidence to guide clinical practice. The use of artificial intelligence to highlight key issues during acute brain injury to support clinicians' decision making should be explored.

Furthermore, the combination of either invasive or non-invasive methods is desirable because they probably assess different aspects of ICC. The integration of different ICC biomarkers might also help discover a better combination of methods and techniques to predict changes in ICC. Studies in specific populations with different diseases and age groups can be valuable for refining applications. Furthermore, studies on the cost-effectiveness of using invasive and/or non-invasive methods are necessary to support the adoption of health technology by hospital managers.

Despite many emergent techniques to assess ICC, most require validation and/or better clinical interpretation. Due to advances in this field and its clear applicability to the neurocritical care context, further studies are required on ICC so that it can be further integrated into clinical practice.

## Author Contributions

GNO, TLR, and RMZ were involved in the study design, collection, analysis, interpretation of data, writing of this article, and decision to submit it for publication. GF, CYH, SB, NNR, and DLSJ helped in the study design and preliminary review of the manuscript. All authors contributed to the article and approved the submitted version.

## Funding

The authors declare that this study received funding from São Paulo Research Foundation (FAPESP grant number: 2017/22173-5 and 2019/14877-8), CAPES (Financial Code 001), and Braincare Desenvolvimento e Inovação Tecnológica S.A. The funders São Paulo Research Foundation (FAPESP) and CAPES were not involved in the study design, collection, analysis, interpretation of data, the writing of this article, or the decision to submit it for publication.

## Conflict of Interest

GF, CYH, and DLSJ were employed by the company Braincare Desenvolvimento e Inovação Tecnológica S.A. TLR, SB, and NNR were health consultants for the company Braincare Desenvolvimento e Inovação Tecnológica S.A. GNO and RMZ received financial support in the form of research grant from the company Braincare Desenvolvimento e Inovação Tecnológica S.A. The funder Braincare Desenvolvimento e Inovação Tecnológica S.A. had the following involvement: study design.

## Publisher's Note

All claims expressed in this article are solely those of the authors and do not necessarily represent those of their affiliated organizations, or those of the publisher, the editors and the reviewers. Any product that may be evaluated in this article, or claim that may be made by its manufacturer, is not guaranteed or endorsed by the publisher.

## Glossary

1. Alpha-delta frequency ratios—alpha and delta are two of the frequencies from EEG monitoring. Their ratio can precede vasospasm and provide some information about cerebral autoregulation impairment.
2. Area under ICP pulse wave—this index is estimated using the ICP waveform, in which the total area under the pulse is calculated. High values of this index are probably associated with poor intracranial compliance.
3. Arterial inflow and venous outflow—in this instance, the main objective is to identify venous outflow impairment by comparison with arterial inflow, as reduced venous outflow and venous pressure tend to increase and cause a decrease in compliance.
4. Brain eigenfrequency spectrum—eigenfrequencies are the resonance frequencies at which the system vibrates in response to the addition of volume. Higher values are associated with high pressure and low compliance.
5. B-waves, along with plateau and slow waves, are derived from ICP through spectral analysis in the frequency domain and represent short repeating elevations in ICP with a frequency of 0.5–2 Hz (or waves/min). B-waves can be associated with faster and greater systolic arterial pressure decrements.
6. CBF—cerebral blood flow.
7. CBF velocity—flow velocity is associated with ICP pulsatility and both increments during the presence of plateau waves, which can be indicative of low CPP and compliance.
8. Cerebral oxygenation—measuring the balance between CBF and oxygen consumption, it is possible to quantify the relative absorption of oxyhemoglobin and deoxyhemoglobin in the cerebral tissue. When the cerebral circulation is compromised by high ICP and poor intracranial compliance, this balance is altered, and oxyhemoglobin diminishes while deoxyhemoglobin increases.
9. Cerebral vasoreactivity: the vascular response to fluctuations in end-tidal carbon dioxide concentration. Impaired reactivity was associated with low ICC.
10. Cerebrospinal compensatory reserve: concept of cerebrospinal capacity to compensate for ICP with an increase in volume. A low compensatory reserve can lead to low compliance.
11. Cerebrospinal elastance—the pressure response to an increase in volume. Once elastance is the inverse of compliance, higher values of CSF elastance are related to low compliance of the system.
12. Cerebrovascular resistance—relationship between CPP and CBF. If the ICP is augmented, this index tends to present lower values and indicates a non-reactive vascular bed.
13. Compensatory-reserve-weighted—for the calculation, the RAP and ICP are considered [compensatory-reserve-weighted = (1—RAP) × ICP]. Lower values indicate better ICC and favorable prognosis.
14. Compliance index—ratio of intracranial volume change to ICP change during a cardiac cycle (dV/dP). A high compliance index is related to the worsening of the compensatory reserve.
15. Compliance slope—logarithm of the relationship between volume and pressure alterations. It is calculated with infusions or withdrawals of fluid, which is observed as the pressure response with the volume elevation. Then, a linear regression analysis is performed to obtain the pressure-volume curve slope. A higher compliance (or pressure-volume curve slope) indicates low ICC.
16. CPP—cerebral perfusion pressure (CPP) is reduced when ICP is elevated and can be used as an indicator of low compliance.
17. CSF absorption—this outcome is related to deficits in CSF absorptive capacity, which is present in some clinical conditions, such as hydrocephalus. This situation may lead to a high intracranial volume and consequent high ICP with low compliance.
18. CSF dynamics—it is possible to identify the reabsorption rate and ventricular CSF reflux through imaging. Abnormal CSF dynamics and increased ventricular reflux are associated with impaired ICC.
19. CSF flow—CSF flow is observed with imaging examinations, and it is interesting to detect ventricular reflux, which can be associated with low compliance when its values are high.
20. CSF outflow conductance—this is the inverse of CSF resistance to outflow and is denoted as volume change per pressure change. A high conductance can lead to an increase in ICC.
21. CSF pressure gradient—alteration of CSF pressure during the cardiac cycle. When this alteration is elevated and remains high, it is associated with low ICC.
22. CSF production rate—this index is the ratio between CSF production and absorption. When production is higher than absorption, the intracranial volume can be increased.
23. CSF pulsatility—this index is essentially the same as ICP pulsatility. This is the difference between the systolic and diastolic pulse peaks of the CSF pulse pressure, and the elevated pulsatility corresponds to poor ICC.
24. CSF pulse pressure—cerebrospinal fluid pulse pressure. It is equivalent to ICP, and its value is usually expressed in mmHg or mmH_2_O.
25. CSF pulse wave velocity—this measure is defined as the spatial distance divided by the transit time required by the pulse wave to cover this distance. The major velocity can be indicative of decreased ICC.
26. CSF reabsorption—this outcome is also related to a deficit of the CSF absorptive capacity and can derive the CSF reabsorption rate, which is calculated as the ratio between absorption and production.
27. CSF resistance to outflow—cerebrospinal fluid resistance to outflow CSF pressure is observed during the infusion test, and the resistance to outflow is the change in pressure per unit of volume per minute. When this index is elevated, CSF does not circulate in the cerebrospinal compartment or is absorbed in the way it should be.
28. CSF volume—CSF volume can be indicative of CSF absorption impairment, which generates an increase in the intracranial volume.
29. CSF volumetric net flow rate: this is an estimation of the CSF flow volume over one cardiac cycle and is related to CSF redistribution and flux. In the low compliance condition and with CSF retrograde flux, the CSF volumetric net flow rate has lower values.
30. CT—computed tomography.
31. CVPT—cerebrovascular pressure transmission. Using spectral analysis in the frequency domain, this index is an indicator of how much arterial blood pressure is transmitted to the ICP. When ICP is elevated, CPP and compliance decrease, and CVPT increases.
32. Delta-displacement analysis—the delta-displacement is the difference between the maximal and minimum water displacement in the white matter during a cardiac cycle. Under low compliance conditions, water molecules in the white matter have larger oscillations by volume loading and, consequently, higher delta-displacement values.
33. EEG—electroencephalography.
34. Elastance coefficient—this index is an exponential pressure-volume relationship and can be demonstrated with the pressure-volume curve, where a steeper curve indicates an increase in brain stiffness and low compliance.
35. Elastance index: this is defined as a change in pressure per unit change in volume (dP/dV). The elastance index is directly proportional to ICP, and higher values can be associated with low ICC.
36. Elasticity slope—this index is defined as the natural logarithm of pressure to the volume infused into the system. Therefore, it is calculated with infusions or withdrawals of fluid. Then, a linear regression analysis using the least-squares technique can be performed to obtain the elasticity slope. A steeper elasticity slope usually indicates low ICC.
37. ETCO_2_—end-tidal carbon dioxide. This outcome is commonly used with ICP, in which better compliance is related to a low correlation between changes in ICP in response to changes in ETCO_2_. Thus, poor compliance can be reflected by a positive correlation between changes in ICP in response to ETCO_2_ changes.
38. Evans index—this index is calculated using the widest diameter of the frontal horns divided by the maximum inner skull width, and it is an index of ventricular size.
39. Finite element model—it is a model that includes the brain, CSF, and skull, with the aim to study these element relationships and simulate altered intracranial volume.
40. Frontal horn ratio -calculated by dividing the frontal horns by brain parenchyma diameters and is indicative of ventricular size. High values of the frontal horn ratio are related to major ventricular size and an increase in intracranial volume.
41. HC—hydrocephaly.
42. High-frequency centroid—this index is acquired using an ICP spectral analysis in the frequency domain and represents the power-weighted mean frequency in the 4 to 15 Hz band of the ICP waveform.
43. ICH—intracranial hypertension.
44. ICP absolute value—intracranial pressure absolute value. Higher values of ICP (≥ 20 mmHg) are widely used as a mark of low ICC since an increase in volume causes a significant increase in ICP, and the compensatory strategies are not enough to keep ICP under stable values.
45. ICP power spectrum—spectral analysis in the frequency domain, which derives slow waves, b-waves, and plateau waves.
46. ICP pulsatility- is the difference between systolic and diastolic ICP pulse peaks (or the difference between P1 and P3 amplitudes). Often, increased pulsatility corresponds to a decrease in ICC.
47. ICP pulse amplitude—derived from the ICP waveform and is the amplitude of the first peak (P1). It is expressed in mmHg and corresponds to the ICP value. When ICC decreases, the ICP pulse amplitude increases. However, this is not always true, as the waveform may change its morphology due to alterations in ICC.
48. ICP pulse latency—another index derived from the ICP waveform, which is the time interval from the low point of the pulse corresponding to the beginning diastolic pressure to the high point of the pulse (corresponding to systolic pressure). In low compliance conditions, the ICP pulse latency tends to be shorter.
49. ICP pulse slope—calculated as the pulse amplitude divided by the pulse latency. A steep ICP slope indicates low compliance once there is a rapid ICP response to the arterial pressure wave.
50. ICP pulse wave P2—similar to ICP pulse amplitude, this index considers the amplitude of the second peak of the ICP morphology and is related to altered ICC when its value is higher than the P1 amplitude.
51. ICP pulse wave ratio P2/P1—relationship between the second (P2) and the first (P1) ICP peaks from a single wave (from waveform morphology). This relationship is expected to be lower than one under normal conditions as P2 is lower than P1. If this value is higher than one, it can be indicative of low ICC.
52. ICP wave rise time—derived from the ICP waveform. This is the time that the pulse takes to reach the first peak (P1). There is a shortening in the rise time in low compliance situations and in response to cardiac pulsation.
53. ICP wave rise time coefficient—this index is also derived from the ICP waveform and is calculated by dividing the pulse amplitude by the wave rise time. When compliance diminishes, this index tends to increase.
54. ICP waveform morphology—the waveform is used to derive other indices. However, it may be used as a classification. It is expected that under normal conditions, the ICP exhibits three peaks in one wave, where the first (P1) is the highest, the second (P2) is lower than P1, and the third (P3) is the lowest. In pathological conditions, these peak magnitudes can differ, as more P2 and P3 rise compared to P1, more changes may be in compliance. It can even lose peak differentiation, and the curve can become rounded in more severe situations.
55. ICP-PCO_2_—relationship between absolute ICP values and carbon dioxide pressure. This correlation is based on PCO_2_ cerebrovascular reactivity, and if changes in cerebral blood vessel diameter alter ICP, it suggests a low compliance condition.
56. Index of autoregulation: correlation coefficient between slow waves in mean blood flow velocity and CPP. A positive correlation indicates a positive association between blood flow velocity, CPP, and impaired autoregulation. Negative correlations or values equal to zero represent good cerebral autoregulation.
57. Intracranial elastance—the same as the elastance index, which is the relationship between a change in pressure per unit change in volume (dP/dV).
58. MCA blood flow velocity—middle cerebral artery blood flow velocity.
59. MRI—magnetic resonance imaging.
60. MR-ICP—ICP is obtained with MRI from measurements of blood and CSF flow to and from the cranium during the cardiac cycle, and their waveforms are used to estimate ICP.
61. NIRS—near infrared spectroscopy.
62. NPH—normal pressure hydrocephalus.
63. Optic nerve sheath diameter: this outcome can be assessed using ultrasonography or fundoscopy. Higher sheath diameter values can be indicative of high ICP.
64. Plateau waves—plateau waves can be derived from an ICP spectral analysis in the frequency domain and are related to sustaining the ICP wave peak from waveform morphology. The presence of plateau waves is associated with delayed ischemic response and lower CPP.
65. Pressure autoregulatory response: the capacity to regulate CPP with high arterial blood pressure oscillations. A low or absence of this response is indicative of poor compliance.
66. Pressure resistive index—instantaneous effects of pressure application and release to the system and its response on the Doppler spectral waveform. Higher values are associated with poor compliance.
67. Pressure-volume compensatory reserve—this index is actually a concept and is expressed based on the elastance coefficient and ICP pulse amplitude. High values of the elastance coefficient and ICP pulse amplitude indicate a decrease in the compensatory reserve, and consequently, low ICC.
68. PRx—pressure reactivity index. It is a measure of the relationship between arterial blood pressure and ICP waves. PRx represents the ICP response through cerebrovascular reactions to changes in blood pressure. Negative values reflect a normal correlation, whereas positive values are associated with non-reactive vascular beds.
69. Pulsatility index—refers to CBF pulsatility in the cerebral arteries. Lower values represent better cerebral perfusion, while higher values are associated with worse cerebral perfusion and can be due to low cerebral compliance.
70. PVI—pressure-volume index. This is defined as the volume necessary to cause a tenfold increase in ICP. Therefore, low PVI values indicate insufficient compensatory reserve and poor compliance.
71. PVR—pressure-volume response is the pressure alteration that occurs due to an injection of volume, usually during an infusion test. Under normal conditions, ICP is expected to demonstrate a small and rapid elevation and return to basal values. However, ICP has an important increment in low compliance situations and does not return to the baseline.
72. Qualitative analysis of ICP waveform: the ICP wave has three pulses (P1, P2, and P3), in which P1>P2>P3. The waveform morphology is affected when the balance between pulses is altered. The waveform can be classified into five patterns: normal, two patterns of possible pathological (when P2 is equal or higher than P1), probably pathological (P2 and P3 are higher than usual), and severe compliance lost (loss of shape).
73. RAC: correlation between ICP pulse amplitude and CPP. It denotes cerebrovascular reactivity, and its value is negative with normal reactivity values. However, a positive RAC indicates worse vascular reactivity and may reflect low ICC.
74. RAP: compensatory reserve index. Correlation coefficient between the mean ICP absolute value and ICP pulse amplitude (from waveform morphology). When its value is approximately zero, the compliance is normal. Values near one represent an exhaustion of compensatory strategies, and values near minus one can be indicative of severe compliance deterioration.
75. Reserve capacity of craniovertebral contents—this index is also a concept that depends on intracranial and ICP.
76. Resistivity index: the relationship between CPP and ICP. Higher values are associated with poor compliance.
77. SAH—subarachnoid hemorrhage.
78. Signal-void phenomenon: through imaging examination, it is possible to look at CSF flow. The signal intensity of the flowing fluid is low when the flow velocity is high. In this case, the signal-void phenomenon is present and is associated with low pressure-buffering capacity.
79. Slow waves—derived from an ICP spectral analysis in the frequency domain and related to cerebral autoregulation, considering the vascular response and brain metabolism. A lower spectral density is associated with worsening cerebral autoregulation.
80. TBI—traumatic brain injury.
81. TDC—transcranial Doppler ultrasonography.
82. TMD—tympanic membrane displacement.
83. Transfer function—measure in the frequency domain that reflects how much of the arterial blood pressure fluctuations are transmitted to the ICP. When ICP is elevated, this relationship is stronger.
84. US—ultrasonography.
85. Venous flow velocity: there is a linear relationship between elevated ICP and high venous flow velocity. Thus, higher values of this index can indirectly indicate impaired ICC.
86. Ventricular dilatation: through imaging examinations, it is possible to identify ventricular dilatation that can occur when the CSF volume is augmented. This index is an indirect method of presuming low ICC.
87. Ventricular size—through imaging examinations, ventricular size measurements can indicate CSF accumulation and ventricular dilatation. This alteration may enable the identification of high ICP and worsening compliance.
88. VPR—volume pressure response. This outcome is usually collected during an infusion test, in which injection or withdrawal of a volume is made, followed by an ICP monitoring period until the ICP returns to baseline. This measurement is indicative of intracranial elastance and an indicator of surgical decompression.
