# Supplementary material for: Intracranial Compliance Concepts and Assessment: A Scoping Review
Source: Front Neurol. 2021 Oct 25;12:756112. doi: 10.3389/fneur.2021.756112 (PMC8573119; doi:10.3389/fneur.2021.756112)
Supplement: Supplementary file 1 [file Table_1.docx]

Table 1. Frequency table of the main related intracranial compliance concepts observed in the literature.

| **Authors** | **Year** | **Type of study** | **Health Condition** | **Physiological definition** | | | **Clinical interpretation** | | | | **Anatomical localization** | | | | |
| --- | --- | --- | --- | --- | --- | --- | --- | --- | --- | --- | --- | --- | --- | --- | --- |
|  |  |  |  | ∆V/∆P | ∆P/∆V elastance | Pressure | Reserve capacity | Accommodation or compensation | Buffering ability | Not reported / Other | Intracranial compartment | Brain | Cerebrospinal space | Vessel | Not reported |
| Adams-Powers (1) | 1997 | Review | SAH |  | X |  |  | X |  |  | X |  |  |  |  |
| Alperin (2) | 2004 | Review | Not reported | X |  |  |  |  |  | X |  |  |  |  | X |
| Alperin et al. (3) | 2014 | CS | Chiari |  | X |  |  |  |  | X | X |  |  |  |  |
| Alperin et al. (4) | 2005 | CS | Chiari |  |  | X |  |  |  | X |  |  |  |  | X |
| Anile et al. (5) | 2010 | CS | NPH | X |  |  |  |  | X |  | X |  |  |  |  |
| Azevedo Filho et al (6) | 1979 | CS | Intracranial aneurysms |  | X |  |  |  |  | X | X |  |  |  |  |
| Bateman (7) | 2013 | Case report | Slit ventricle syndrome | X |  |  |  | X |  |  |  | X |  |  |  |
| Bateman et al. (8) | 2003 | CS | NPH | X |  |  |  |  |  | X |  |  |  | X |  |
| Behzadnia et al. (9) | 2018 | CS | TBI | X |  |  |  |  |  | X | X |  |  |  |  |
| Bentsen (10) | 2005 | Clinical trial protocol | SAH |  |  | X |  |  |  | X | X |  |  |  |  |
| Bentsen et al. (11) | 2008 | CS | SAH | X |  |  |  |  |  | X | X |  |  |  |  |
| Borgesen et al. (12) | 1979 | Experimental | NPH | X |  |  |  |  |  | X |  |  |  |  | X |
| Burchiel et al. (13) | 1981 | CS | TBI, SAH | X |  |  |  |  |  | X |  | X |  |  |  |
| De Simone et al. (14) | 2019 | Review | ICH | X |  |  |  | X |  |  |  | X |  |  |  |
| Dias et al. (15) | 2021 | CS | HC |  |  | X | X |  |  |  |  |  | X |  |  |
| Diringer (16) | 1993 | Review | SAH | X |  |  |  |  |  | X | X |  |  |  |  |
| Doyle-Mark (17) | 1992 | Review | Mixed | X |  |  |  | X |  |  | X |  |  |  |  |
| Eide (18) | 2005 | CS | Children (HC) |  | X |  |  |  |  | X |  |  |  |  | X |
| Eide et al. (19) | 2014 | CS | NPH and ICH |  | X |  | X |  |  |  | X |  |  |  |  |
| Eide et al. (20) | 2010 | CS | SAH |  | X |  | X |  |  |  | X |  |  |  |  |
| Eide et al. (21) | 2016 | Research report | ICH |  | X |  | X |  |  |  | X |  |  |  |  |
| Eide et al. (22) | 2020 | CS | NPH | X |  |  | X |  |  |  | X |  |  |  |  |
| Eide-Sorteberg (23) | 2006 | CS | SAH | X |  |  | X |  |  |  | X |  |  |  |  |
| Eide-Sorteberg (24) | 2007 | CS | SAH | X |  |  |  |  |  | X | X |  |  |  |  |
| Eide-Stanisic (25) | 2010 | CS | NPH |  | X |  | X |  |  |  | X |  |  |  |  |
| Elixmann et al. (26) | 2014 | CS | NPH | X |  |  |  | X |  |  | X |  |  |  |  |
| Foltz et al. (27) | 1990 | CS | HC | X |  |  |  | X |  |  | X |  |  |  |  |
| Foltz-Blanks (28) | 1988 | CS | HC | X |  |  |  | X |  |  | X |  |  |  |  |
| Fukuhara et al. (29) | 2001 | Experimental | HC |  | X |  | X |  | X |  |  | X |  |  |  |
| Germon (30) | 1994 | Review | Mixed |  | X |  |  |  |  | X |  | X |  |  |  |
| Germon (31) | 1988 | Review | Not reported | X |  |  |  |  |  | X | X |  |  |  |  |
| Gholampour-Fatouraee (32) | 2021 | Computational simulation | HC | X |  |  |  |  |  | X | X |  |  |  |  |
| González-Darder and Barcia-Salorio (33) | 1989 | Experimental | HC |  | X |  |  |  |  | X | X |  |  | X |  |
| Greitz (34) | 2004 | Review | HC | X |  |  |  |  | X |  |  |  |  |  | X |
| Heldt et al. (35) | 2019 | Review | TBI |  | X |  |  |  | X |  | X |  |  |  |  |
| Hickey et al. (36) | 2009 | CS | Mixed | X |  |  |  | X |  |  |  | X |  |  |  |
| Jacobsen et al. (37) | 2020 | CS | NPH |  |  | X | X |  |  |  | X |  |  |  |  |
| Jaeger-Layton (38) | 2000 | Overview | HC |  | X |  | X |  |  |  |  |  | X |  |  |
| Jha & Kochanek (39) | 2018 | Review | TBI | X |  |  |  |  |  | X | X |  |  |  |  |
| Johnson et al. (40) | 1999 | Experimental | HC | X |  |  | X |  |  |  |  | X |  |  |  |
| Kan et al. (41) | 2015 | CS | NPH |  | X |  |  |  | X |  | X |  |  |  |  |
| Kiening et al. (42) | 2003 | CS | TBI | X |  |  |  |  |  | X | X |  |  |  |  |
| Kim et al. (43) | 2012 | CS | TBI | X |  |  |  |  |  | X |  | X |  |  |  |
| Kim et al. (44) | 2009 | CS | NPH |  |  | X |  |  |  | X |  |  | X |  |  |
| Lai et al. (45) | 2016 | CS | Mixed | X |  |  |  | X |  |  | X |  |  |  |  |
| Lang et al. (46) | 2003 | Technical notes | TBI, SAH, ICH |  | X |  |  | X |  |  |  | X |  |  |  |
| Latka et al. (47) | 2007 | CS | Mixed |  | X |  |  |  |  | X |  |  | X |  |  |
| Lesniak et al. (48) | 2002 | CS | NPH | X |  |  |  |  |  | X | X |  |  |  |  |
| Lewis (49) | 1998 | Review | TBI | X |  |  |  | X |  |  | X |  |  |  |  |
| Lim et al. (50) | 1973 | Experimental | Aqueductal obstruction | X |  |  |  |  |  | X |  |  |  |  | X |
| Lindstrom et al. (51) | 2018 | CS | NPH |  | X |  | X |  |  |  | X |  |  |  |  |
| Lodi et al. (52) | 1998 | MM | Not reported |  |  | X |  |  |  | X | X |  |  |  |  |
| Lokossou et al. (53) | 2018 | CS | HC | X |  |  |  | X |  |  |  |  | X |  |  |
| March K (54) | 2000 | Review | TBI |  |  | X |  |  |  | X |  |  |  |  | X |
| Marshall et al. (55) | 2013 | Review | Not reported | X |  |  |  |  |  | X | X |  |  |  |  |
| Meier et al. (56) | 2002 | Experimental | HC |  |  | X |  |  |  | X |  |  | X |  |  |
| Meier et al. (57) | 1999 | MM | Not reported |  |  | X |  |  |  | X |  |  | X |  |  |
| Miyati et al. (58) | 2007 | CS | NPH | X |  |  |  |  |  | X | X |  |  |  |  |
| Morandi et al. (59) | 2006 | Medical hypotheses | HC | X |  |  |  |  |  | X |  |  |  |  | X |
| Mursch et al. (60) | 1995 | CS | TBI | X |  |  |  |  | X |  | X |  |  |  |  |
| Ng et al. (61) | 2005 | Pilot study | SAH | X |  |  |  |  |  | X | X |  |  |  |  |
| Nujaimin et al. (62) | 2009 | CS | TBI | X |  |  |  |  |  | X |  |  |  |  | X |
| Nyholm et al. (63) | 2017 | CS | TBI | X |  |  |  |  |  | X | X |  |  |  |  |
| Paraguassu et al. (64) | 2021 | CR | HC |  |  | X |  |  |  | X | X |  |  |  |  |
| Park et al. (65) | 2012 | CS | NPH |  | X |  |  |  |  | X |  | X |  |  |  |
| Park et al. (66) | 2010 | Experimental | HC |  | X |  |  |  |  | X |  | X |  |  |  |
| Piper et al. (67) | 1999 | CS | NPH | X |  |  |  |  |  | X | X |  |  |  |  |
| Portella et al. (68) | 2005 | CS | TBI | X |  |  |  |  | X |  | X |  |  |  |  |
| Qvarlander et al. (69) | 2013 | CS | NPH |  | X |  |  |  |  | X |  |  |  |  | X |
| Qvarlander et al. (70) | 2014 | MM | NPH |  | X |  |  | X |  |  |  |  | X |  |  |
| Raabe et al. (71) | 1999 | CS | HC, SAH, TBI | X |  |  |  |  | X |  | X |  |  |  |  |
| Rasulo et al. (72) | 2020 | CS | ICH |  |  | X |  |  |  | X | X |  |  |  |  |
| Robba et al. (73) | 2017 | Review | Mixed | X |  |  |  | X |  |  |  | X |  |  |  |
| Ross-Eynon (74) | 2005 | CS | Mixed |  | X |  |  |  |  | X |  | X |  |  |  |
| Saehle-Eide (75) | 2015 | CS | HC |  | X |  | X |  |  |  | X |  |  |  |  |
| Sahuquillo et al. (76) | 1991 | CS | NPH |  |  | X |  |  |  | X |  |  |  | X |  |
| Salci et al. (77) | 2004 | Experimental | TBI | X |  |  |  | X |  |  | X |  |  |  |  |
| Salci et al. (78) | 2006 | Experimental | TBI | X |  |  |  | X |  |  | X |  |  |  |  |
| Schaller-Graf (79) | 2005 | Review | Mixed | X |  |  | X |  |  |  | X |  |  |  |  |
| Schummer et al. (80) | 2003 | CS | Mixed | X |  |  |  | X |  |  | X |  |  |  |  |
| Smith et al. (81) | 2012 | Experimental | TBI | X |  |  |  |  |  | X | X |  |  |  |  |
| Solheim et al. (82) | 2008 | Case report | Seizure |  | X |  | X |  |  |  | X |  |  |  |  |
| Sonnabend et al. (83) | 2021 | MM | NPH | X |  |  |  |  |  | X |  |  |  | X |  |
| Sood et al. (84) | 2004 | CS | Shunt therapy | X |  |  |  |  |  | X |  |  |  |  | X |
| Tain & Alperin (85) | 2009 | MM | Not reported | X |  |  |  | X |  |  | X |  |  |  |  |
| Tain et al. (86) | 2011 | CS | ICH | X |  |  |  | X |  |  |  |  |  |  | X |
| Taylor (87) | 1994 | CS | Children (mixed) |  | X |  |  |  |  | X | X |  |  |  |  |
| Timofeev et al. (88) | 2008 | CS | TBI | X |  |  |  |  |  | X | X |  |  |  |  |
| Tisell et al. (89) | 2002 | CS | HC |  | X |  |  |  | X |  |  |  | X |  |  |
| Wagshul et al. (90) | 2011 | Review | Mixed | X |  |  |  |  |  | X |  |  |  |  | X |
| Wettervik et al. (91) | 2020 | CS | TBI | X |  |  |  |  |  | X | X |  |  |  |  |
| Wilkinson et al. (92) | 1979 | CS | Mixed |  | X |  |  |  |  | X |  | X |  |  |  |
| Wolf et al. (93) | 2021 | CS | Children (TBI) | X |  |  |  |  |  | X | X |  |  |  |  |
| Xu et al. (94) | 2021 | Experimental | HC | X |  |  |  | X |  |  | X |  |  |  |  |
| Yang et al. (95) | 1993 | Experimental | Acute hypoxia |  | X |  |  |  |  | X | X |  |  |  |  |
| Tirado-Caballero et al. (96) | 2021 | CS | Mixed |  | X |  |  |  |  | X |  |  |  |  | X |
| Shapiro et al. (97) | 1980 | CS | Mixed | X |  |  |  |  |  | X |  |  |  |  | X |
| Lodi et al. (98) | 1997 | MM | Mixed | X |  |  | X |  |  |  |  |  | X |  |  |
| Qiu et al. (99) | 2010 | MM | Mixed | X |  |  | X |  |  |  | X |  |  |  |  |
| Ursino et al. (100) | 1998 | MM | Mixed | X |  |  | X |  |  |  | X |  |  |  |  |
| Portella et al. (101) | 2002 | CS | TBI | X |  |  |  | X |  |  | X |  |  |  |  |
| Ursino et al. (102) | 2000 | MM | TBI | X |  |  | X |  |  |  | X |  |  |  |  |
| Kazimierska et al. (103) | 2021 | CS | NPH | X |  |  |  |  | X |  |  |  | X |  |  |

Abbreviations: ∆P, delta pressure; ∆V, delta volume; CS, clinical study; HC, hydrocephalus; ICH, intracranial hypertension; Mixed, more than two neurological conditions; MM, mathematical model; NPH, normal pressure hydrocephalus; SAH, subarachnoid hemorrhage; TBI, traumatic brain injury.

***References***

1. Adams RE, Powers WJ. Management of hypertension in acute intracerebral hemorrhage. Critical Care Clinics. 1997 Jan;13(1):131–61. doi: [10.1016/S0749-0704(05)70299-2](https://doi-org.ez31.periodicos.capes.gov.br/10.1016/S0749-0704(05)70299-2)

2. Alperin N. MR–Intracranial Compliance and Pressure: A Method for Noninvasive Measurement of Important Neurophysiologic Parameters. Methods in Enzymology. 2004;386:323–49. doi: [10.1016/S0076-6879(04)86016-6](https://doi-org.ez31.periodicos.capes.gov.br/10.1016/S0076-6879(04)86016-6)

3. Alperin N, Loftus JR, Oliu CJ, Bagci AM, Lee SH, Ertl-Wagner B, et al. Magnetic Resonance Imaging Measures of Posterior Cranial Fossa Morphology and Cerebrospinal Fluid Physiology in Chiari Malformation Type I. Neurosurgery. 2014 Nov 1;75(5):515–22. doi: [10.1227/NEU.0000000000000507](https://doi-org.ez31.periodicos.capes.gov.br/10.1227/NEU.0000000000000507)

4. Alperin N, Sivaramakrishnan A, Lichtor T. Magnetic resonance imaging—based measurements of cerebrospinal fluid and blood flow as indicators of intracranial compliance in patients with Chiari malformation. Journal of Neurosurgery. 2005 Jul;103(1):46–52. doi: [10.3171/jns.2005.103.1.0046](https://doi.org/10.3171/jns.2005.103.1.0046)

5. Anile C, De Bonis P, Albanese A, Di Chirico A, Mangiola A, Petrella G, et al. Selection of patients with idiopathic normal-pressure hydrocephalus for shunt placement: a single-institution experience: Clinical article. JNS. 2010 Jul;113(1):64–73. doi: [10.3171/2010.1.JNS091296](https://doi.org/10.3171/2010.1.JNS091296)

6. Azevedo Filho HRC de, Adams CBT, Kerr J. Intracranial compliance during the post-operative period after surgery for intracranial aneurysms. Arq Neuro-Psiquiatr. 1979 Sep;37(3):250–4. doi: [10.1590/S0004-282X1979000300003](https://doi.org/10.1590/S0004-282X1979000300003)

7. Bateman GA. Hypertensive slit ventricle syndrome: pseudotumor cerebri with a malfunctioning shunt?: Report of 3 cases. JNS. 2013 Dec;119(6):1503–10. doi: [10.3171/2013.7.JNS13390](https://doi.org/10.3171/2013.7.JNS13390)

8. Bateman GA. The reversibility of reduced cortical vein compliance in normal-pressure hydrocephalus following shunt insertion. Neuroradiology. 2003 Feb;45(2):65–70. doi: 10.1007/s00234-002-0901-0

9. Behzadnia H, Emamhadi M, Chabok SY, Alijani B, Jafari S, Andalib S. The Correlation Between Intracranial Pressure Amplitude and Glasgow Outcome Scale in Patients with Traumatic Brain Injury. Trauma Mon. 2018 Nov;23(6):1–5. doi: [10.5812/traumamon.79490](http://dx.doi.org/10.5812/traumamon.79490)

10. Bentsen G. ICP Versus Intracranial Compliance (ICC) Guided Management in SAH. ClinicalTrials.gov. 2011 Jan. Available from https://clinicaltrials.gov/ct2/show/NCT00248690

11. Bentsen G, Stubhaug A, Eide PK. Differential effects of osmotherapy on static and pulsatile intracranial pressure: Critical Care Medicine. 2008 Aug;36(8):2414–9. doi: 10.1097/CCM.0b013e318180fe04

12. Børgesen SE, Gjerris F, Sørensen SC. Cerebrospinal fluid conductance and compliance of the craniospinal space in normal-pressure hydrocephalus: A comparison between two methods for measuring conductance to outflow. Journal of Neurosurgery. 1979 Oct;51(4):521–5. doi: [10.3171/jns.1979.51.4.0521](https://doi.org/10.3171/jns.1979.51.4.0521)

13. Burchiel KJ, Steege TD, Wyler AR. Intracranial Pressure Changes in Brain-injured Patients Requiring Positive End-Expiratory Pressure Ventilation. Neurosurgery. 1981;8(4):443–9. doi: [10.1227/00006123-198104000-00007](https://doi-org.ez31.periodicos.capes.gov.br/10.1227/00006123-198104000-00007)

14. De Simone R, Ranieri A, Sansone M, Marano E, Russo CV, Saccà F, et al. Dural sinus collapsibility, idiopathic intracranial hypertension, and the pathogenesis of chronic migraine. Neurol Sci. 2019 May;40(S1):59–70. doi: 10.1007/s10072-019-03775-w

15. Dias SF, Jehli E, Haas-Lude K, Bevot A, Okechi H, Zipfel J, et al. Ventriculomegaly in children: nocturnal ICP dynamics identify pressure-compensated but active paediatric hydrocephalus. Childs Nerv Syst. 2021 Jun;37(6):1883–93. doi: 10.1007/s00381-021-05164-1

16. Diringer MN. Intracerebral hemorrhage: Pathophysiology and management. Neurologic Critical Care. 1993;21(10):1591–1603. doi: 10.1097/00003246-199310000-00032

17. Doyle DJ, Mark PWS. Analysis of intracranial pressure. Journal of Clinical Monitoring. 1992;8(1):81–90. doi: 10.1007/BF01618093

18. Eide PK. Assessment of Childhood Intracranial Pressure Recordings Using a New Method of Processing Intracranial Pressure Signals. Pediatr Neurosurg. 2005;41(3):122–30. doi: [10.1159/000085868](https://doi-org.ez31.periodicos.capes.gov.br/10.1159/000085868)

19. Eide PK, Eidsvaag VA, Hansson H-A. Antisecretory factor (AF) exerts no effects on intracranial pressure (ICP) waves and ICP in patients with idiopathic normal pressure hydrocephalus and idiopathic intracranial hypertension. Journal of the Neurological Sciences. 2014 Aug;343(1–2):132–7. doi: [10.1016/j.jns.2014.05.054](https://doi-org.ez31.periodicos.capes.gov.br/10.1016/j.jns.2014.05.054)

20. Eide PK, Rapoport BI, Gormley WB, Madsen JR. A dynamic nonlinear relationship between the static and pulsatile components of intracranial pressure in patients with subarachnoid hemorrhage: Clinical article. JNS. 2010 Mar;112(3):616–25. doi: [10.3171/2009.7.JNS081593](https://doi.org/10.3171/2009.7.JNS081593)

21. Eide PK. The correlation between pulsatile intracranial pressure and indices of intracranial pressure-volume reserve capacity: results from ventricular infusion testing. JNS. 2016 Dec;125(6):1493–503. doi: [10.3171/2015.11.JNS151529](https://doi.org/10.3171/2015.11.JNS151529)

22. Eide PK, Pripp AH, Ringstad G. Magnetic resonance imaging biomarkers of cerebrospinal fluid tracer dynamics in idiopathic normal pressure hydrocephalus. Brain Communications. 2020 Jul 1;2(2):1–16. doi: [10.1093/braincomms/fcaa187](https://doi.org/10.1093/braincomms/fcaa187)

23. Eide PK, Sorteberg W. Intracranial pressure levels and single wave amplitudes, Glasgow Coma Score and Glasgow Outcome Score after subarachnoid haemorrhage. Acta Neurochir (Wien). 2006 Dec;148(12):1267–76. doi: 10.1007/s00701-006-0908-0

24. Eide PK, Sorteberg W. Association among intracranial compliance, intracranial pulse pressure amplitude and intracranial pressure in patients with intracranial bleeds. Neurological Research. 2007 Dec;29(8):798–802. doi: [10.1179/016164107X224132](https://doi-org.ez31.periodicos.capes.gov.br/10.1179/016164107X224132)

25. Eide PK. Cerebral microdialysis and intracranial pressure monitoring in patients with idiopathic normal-pressure hydrocephalus: association with clinical response to extended lumbar drainage and shunt surgery. J Neurosurg. 2010;112:11. doi: [10.3171/2009.5.JNS09122](https://doi.org/10.3171/2009.5.JNS09122)

26. Elixmann IM, Kwiecien M, Goffin C, Walter M, Misgeld B, Kiefer M, et al. Control of an Electromechanical Hydrocephalus Shunt—a New Approach. IEEE Trans Biomed Eng. 2014 Sep;61(9):2379–88. doi: [10.1109/TBME.2014.2308927](https://doi-org.ez31.periodicos.capes.gov.br/10.1109/TBME.2014.2308927)

27. Foltz EL, Blanks JP, Yonemura K. CSF pulsatility in hydrocephalus: respiratory effect on pulse wave slope as an indicator of intracranial compliance. Neurological Research. 1990 Jun;12(2):67–74. doi: [10.1080/01616412.1990.11739918](https://doi-org.ez31.periodicos.capes.gov.br/10.1080/01616412.1990.11739918)

28. Foltz EL, Blanks JP. Symptomatic low intracranial pressure in shunted hydrocephalus. J Neurosurg. 1988;68:401–8. doi: [10.3171/jns.1988.68.3.0401](https://doi.org/10.3171/jns.1988.68.3.0401)

29. Fukuhara T, Luciano MG, Brant CL, Klauscie J. Effects of ventriculoperitoneal shunt removal on cerebral oxygenation and brain compliance in chronic obstructive hydrocephalus. Journal of Neurosurgery. 2001 Apr;94(4):573–81. doi: [10.3171/jns.2001.94.4.0573](https://doi.org/10.3171/jns.2001.94.4.0573)

30. Germon K. Intracranial pressure monitoring in the 1990s. Critical Care Nursing Quarterly. 1994 May;17(1):21–32. doi: [10.1097/00002727-199405000-00004](https://doi.org/10.1097/00002727-199405000-00004)

31. Germon K. Interpretation of ICP pulse waves to determine intracerebral compliance. Journal of Neuroscience Nursing. 1988 Dec;20(6):344–350. doi: [10.1097/01376517-198812000-00004](https://doi.org/10.1097/01376517-198812000-00004)

32. Gholampour S, Fatouraee N. Boundary conditions investigation to improve computer simulation of cerebrospinal fluid dynamics in hydrocephalus patients. Commun Biol. 2021 Dec;4(1):394. doi: [10.1038/s42003-021-01920-w](https://doi.org/10.1038/s42003-021-01920-w)

33. González-Darder JM, Barcia-Salorio JL. Pulse Amplitude and Volume-pressure Relationships in Experimental Hydrocephalus. 1989;97:166–170. doi: 10.1007/BF01772830

34. Greitz D. Radiological assessment of hydrocephalus: new theories and implications for therapy. Neurosurg Rev. 2004;27:145–165. doi: [10.1007/s10143-004-0326-9](https://doi-org.ez31.periodicos.capes.gov.br/10.1007/s10143-004-0326-9)

35. Heldt T, Zoerle T, Teichmann D, Stocchetti N. Intracranial Pressure and Intracranial Elastance Monitoring in Neurocritical Care. Annu Rev Biomed Eng. 2019 Jun 4;21(1):523–49. doi: [10.1146/annurev-bioeng-060418-052257](https://doi-org.ez31.periodicos.capes.gov.br/10.1146/annurev-bioeng-060418-052257)

36. Hickey JV, Olson DM, Turner DA. Intracranial Pressure Waveform Analysis During Rest and Suctioning. Biological Research For Nursing. 2009 Oct;11(2):174–86. doi: 10.1177/1099800409332902

37. Jacobsen HH, Sandell T, Jørstad ØK, Moe MC, Ringstad G, Eide PK. In Vivo Evidence for Impaired Glymphatic Function in the Visual Pathway of Patients With Normal Pressure Hydrocephalus. Invest Ophthalmol Vis Sci. 2020 Nov 17;61(13):24. doi: [10.1167/iovs.61.13.24](https://doi.org/10.1167/iovs.61.13.24)

38. Jaeger KM, Layton TN. Hydrodynamic principles in hydrocephalus The engineer’s perspective. Neurological Research. 2000 Jan;22(1):97–101. doi: 10.1080/01616412.2000.11741042

39. Jha RM, Kochanek PM. A Precision Medicine Approach to Cerebral Edema and Intracranial Hypertension after Severe Traumatic Brain Injury: Quo Vadis? Curr Neurol Neurosci Rep. 2018 Dec;18(12):105. doi: [10.1007/s11910-018-0912-9](https://doi-org.ez31.periodicos.capes.gov.br/10.1007/s11910-018-0912-9)

40. Johnson MJ, Ayzman I, Wood AS, Tkach JA, Klauschie J, Skarupa DJ, et al. Development and characterization of an adult model of obstructive hydrocephalus. Journal of Neuroscience Methods. 1999 Sep;91(1–2):55–65. doi: [10.1016/S0165-0270(99)00072-2](https://doi-org.ez31.periodicos.capes.gov.br/10.1016/S0165-0270(99)00072-2)

41. Kan H, Miyati T, Mase M, Osawa T, Ohno N, Kasai H, et al. Dynamic state of water molecular displacement of the brain during the cardiac cycle in idiopathic normal pressure hydrocephalus. Computerized Medical Imaging and Graphics. 2015 Mar;40:88–93. doi: [10.1016/j.compmedimag.2014.12.004](https://doi-org.ez31.periodicos.capes.gov.br/10.1016/j.compmedimag.2014.12.004)

42. Kiening K, Schoening W, Stover J, Unterberg A. Continuous monitoring of intracranial compliance after severe head injury: relation to data quality, intracranial pressure and brain tissue PO _2_. British Journal of Neurosurgery. 2003 Jan;17(4):311–8. doi: [10.1080/02688690310001601199](https://doi-org.ez31.periodicos.capes.gov.br/10.1080/02688690310001601199)

43. Kim D-J, Czosnyka Z, Kasprowicz M, Smieleweski P, Baledent O, Guerguerian A-M, et al. Continuous Monitoring of the Monro-Kellie Doctrine: Is It Possible? Journal of Neurotrauma. 2012 May;29(7):1354–63. doi: 10.1089/neu.2011.2018

44. Kim D-J, Czosnyka Z, Keong N, Radolovich DK, Smielewski P, Sutcliffe MPF, et al. Index of cerebrospinal compensatory reserve in hydrocephalus. Neurosurgery. 2009 Mar 1;64(3):494–502. doi: [10.1227/01.NEU.0000338434.59141.89](https://doi-org.ez31.periodicos.capes.gov.br/10.1227/01.NEU.0000338434.59141.89)

45. Lai H-Y, Lee C-H, Lee C-Y. The Intracranial Volume Pressure Response in Increased Intracranial Pressure Patients: Clinical Significance of the Volume Pressure Indicator. Boltze J, editor. PLoS ONE. 2016 Oct 10;11(10):e0164263. doi: [10.1371/journal.pone.0164263](https://doi.org/10.1371/journal.pone.0164263)

46. Lang EW, Paulat K, Witte C, Zolondz J, Mehdorn HM. Noninvasive intracranial compliance monitoring: Technical note and clinical results. Journal of Neurosurgery. 2003 Jan;98(1):214–8. doi: [10.3171/jns.2003.98.1.0214](https://doi.org/10.3171/jns.2003.98.1.0214)

47. Latka M, Kolodziej W, Turalska M, Latka D, Zub W, West BJ. Wavelet assessment of cerebrospinal compensatory reserve and cerebrovascular reactivity. Physiol Meas. 2007 May 1;28(5):465–79. doi: 10.1088/0967-3334/28/5/002

48. Lesniak MS, Clatterbuck RE, Rigamonti D, Williams MA. Low pressure hydrocephalus and ventriculomegaly: hysteresis, non-linear dynamics, and the benefits of CSF diversion. British Journal of Neurosurgery. 2002 Jan;16(6):555–61. doi: [10.1080/02688690209168360](https://doi-org.ez31.periodicos.capes.gov.br/10.1080/02688690209168360)

49. Lewis S. Cerebrovascular pressure transmission analysis as a guide to the pathophysiology of raised intracranial pressure. Clin Exp Pharmacol Physiol. 1998 Nov;25(11):947–50. doi: [10.1111/j.1440-1681.1998.tb02350.x](https://doi-org.ez31.periodicos.capes.gov.br/10.1111/j.1440-1681.1998.tb02350.x)

50. Lim ST, Potts DG, Deonarine V, Deck MDF. Ventricular compliance in dogs with and without aqueductal obstruction. Journal of Neurosurgery. 1973 Oct;39(4):463–73. doi: [10.3171/jns.1973.39.4.0463](https://doi.org/10.3171/jns.1973.39.4.0463)

51. Lindstrøm EK, Ringstad G, Mardal K-A, Eide PK. Cerebrospinal fluid volumetric net flow rate and direction in idiopathic normal pressure hydrocephalus. NeuroImage: Clinical. 2018;20:731–41. doi: [10.1016/j.nicl.2018.09.006](https://doi-org.ez31.periodicos.capes.gov.br/10.1016/j.nicl.2018.09.006)

52. Lodi CA, Ter Minassian A, Beydon L, Ursino M. Modeling cerebral autoregulation and CO _2_ reactivity in patients with severe head injury. American Journal of Physiology-Heart and Circulatory Physiology. 1998 May 1;274(5):H1729–41. doi: 10.1152/ajpheart.1998.274.5.H1729

53. Lokossou A, Balédent O, Garnotel S, Page G, Balardy L, Czosnyka Z, et al. ICP Monitoring and Phase-Contrast MRI to Investigate Intracranial Compliance. Intracranial Pressure & Neuromonitoring XVI, Acta Neurochirugica Supplement. 2018;126:247–53. doi: [10.1007/978-3-319-65798-1_50](https://doi.org/10.1007/978-3-319-65798-1_50)

54. March K. Intracranial Pressure Monitoring and Assessing Intracranial Compliance in Brain Injury. Critical Care Nursing Clinics of North America. 2000 Dec;12(4):429–36. doi: [10.1016/S0899-5885(18)30079-0](https://doi.org/10.1016/S0899-5885(18)30079-0)

55. Marshall SA, Kalanuria A, Markandaya M, Nyquist PA. Management of Intracerebral Pressure in the Neurosciences Critical Care Unit. Neurosurgery Clinics of North America. 2013 Jul;24(3):361–73. doi: [10.1016/j.nec.2013.03.004](https://doi.org/10.1016/j.nec.2013.03.004)

56. Meier U, Kiefer M, Bartels P. The ICP-dependency of resistance to cerebrospinal fluid outflow: a new mathematical method for CSF-parameter calculation in a model with H-TX rats. Journal of Clinical Neuroscience. 2002 Jan;9(1):58–63. doi: [10.1054/jocn.2001.0930](https://doi.org/10.1054/jocn.2001.0930)

57. Meier U, Zeilinger FSt, Kintzel D. Diagnostic in Normal Pressure Hydrocephalus: A Mathematical Model for Determination of the ICP-Dependent Resistance and Compliance. Acta Neurochirurgica. 1999 Sep 22;141(9):941–8. doi: [10.1007/s007010050400](https://doi.org/10.1007/s007010050400)

58. Miyati T, Mase M, Kasai H, Hara M, Yamada K, Shibamoto Y, et al. Noninvasive MRI assessment of intracranial compliance in idiopathic normal pressure hydrocephalus. J Magn Reson Imaging. 2007 Aug;26(2):274–8. doi: [10.1002/jmri.20999](https://doi.org/10.1002/jmri.20999)

59. Morandi X, Amlashi SFA, Riffaud L. A dynamic theory for hydrocephalus revealing benign intraspinal tumours: Tumoural obstruction of the spinal subarachnoid space reduces total CSF compartment compliance. Medical Hypotheses. 2006 Jan;67(1):79–81. doi: [10.1016/j.mehy.2006.01.005](https://doi.org/10.1016/j.mehy.2006.01.005)

60. Mursch K, Vogelsang JP, Zimmerer B, Ludwig HC, Behnke J, Markakis E. Bedside measurement of the third ventricle’s diameter during episodes of arising intracranial pressure after head trauma: Using transcranial real-time sonography for a non-invasive examination of intracranial compensation mechanisms. Acta neurochir. 1995 Mar;137(1–2):19–24. doi: [10.1007/BF02188774](https://doi.org/10.1007/bf02188774)

61. Ng SCP, Poon WS, Chan MTV. Cerebral haemodynamic assessment in patients with thalamic haemorrhage: a pilot study with continuous compliance monitoring. Acta Neurochir. 2005;95:299–301. doi: [10.1007/3-211-32318-x_61](https://doi.org/10.1007/3-211-32318-x_61)

62. Nujaimin U, Saufi A, A. Rahman G, Badrisyah I, Sani S, Zamzuri I, et al. Post Traumatic Cerebral Oedema in Severe Head Injury is Related to Intracranial Pressure and Cerebral Perfusion Pressure but not to Cerebral Compliance. Asian Journal of Surgery. 2009 Jul;32(3):157–62. doi: [10.1016/s1015-9584(09)60387-0](https://doi.org/10.1016/s1015-9584(09)60387-0)

63. Nyholm L, Howells T, Enblad P. Predictive Factors That May Contribute to Secondary Insults With Nursing Interventions in Adults With Traumatic Brain Injury. Journal of Neuroscience Nursing. 2017 Feb;49(1):49–55. doi: [10.1097/JNN.0000000000000260](https://doi.org/10.1097/jnn.0000000000000260)

64. Paraguassu G, Khilnani M, Rabelo NN, Cobos LD, Frigieri G. Case Report: Untreatable Headache in a Child With Ventriculoperitoneal Shunt Managed by Use of New Non-invasive Intracranial Pressure Waveform. Front Neurosci. 2021 Feb 10;15:1–4. doi: [10.3389/fnins.2021.601945](https://doi.org/10.3389/fnins.2021.601945)

65. Park E-H, Eide PK, Zurakowski D, Madsen JR. Impaired pulsation absorber mechanism in idiopathic normal pressure hydrocephalus: Laboratory investigation. JNS. 2012 Dec;117(6):1189–96. doi: [10.3171/2012.9.JNS121227](https://doi.org/10.3171/2012.9.jns121227)

66. Park E-H, Dombrowski S, Luciano M, Zurakowski D, Madsen JR. Alterations of pulsation absorber characteristics in experimental hydrocephalus: Laboratory investigation. PED. 2010 Aug;6(2):159–70. doi: [10.1080/02688699943097](https://doi.org/10.1080/02688699943097)

67. Piper I, Spiegelberg A, Whittle I, Signorini D, Mascia L. A comparative study of the Spiegelberg Compliance Device with a manual volume-injection method: a clinical evaluation in patients with hydrocephalus. British Journal of Neurosurgery. 1999 Jan;13(6):581–6.

68. Portella G, Cormio M, Citerio G, Contant C, Kiening K, Enblad P, et al. Continuous cerebral compliance monitoring in severe head injury: its relationship with intracranial pressure and cerebral perfusion pressure. Acta Neurochir (Wien). 2005 Jul;147(7):707–13. doi: [10.1007/s00701-005-0537-z](https://doi.org/10.1007/s00701-005-0537-z)

69. Qvarlander S, Lundkvist B, Koskinen L-OD, Malm J, Eklund A. Pulsatility in CSF dynamics: pathophysiology of idiopathic normal pressure hydrocephalus. Journal of Neurology, Neurosurgery & Psychiatry. 2013 Jul 1;84(7):735–41. doi: [10.1136/jnnp-2012-302924](http://dx.doi.org/10.1136/jnnp-2012-302924)

70. Qvarlander S, Malm J, Eklund A. CSF dynamic analysis of a predictive pulsatility-based infusion test for normal pressure hydrocephalus. Med Biol Eng Comput. 2014 Jan;52(1):75–85. doi: [10.1007/s11517-013-1110-1](https://doi.org/10.1007/s11517-013-1110-1)

71. Raabe A, Czosnyka M, Piper I, Seifert V. Monitoring of Intracranial Compliance: Correction for a Change in Body Position. Acta Neurochirurgica. 1999 Jan 25;141(1):31–6. doi: [10.1007/s007010050263](https://doi.org/10.1007/s007010050263)

72. Rasulo F, Piva S, Park S, Oddo M, Megjhani M, Cardim D, et al. The Association Between Peri-Hemorrhagic Metabolites and Cerebral Hemodynamics in Comatose Patients With Spontaneous Intracerebral Hemorrhage: An International Multicenter Pilot Study Analysis. Front Neurol. 2020 Oct 26;11:568536. doi: [10.3389/fneur.2020.568536](https://doi.org/10.3389/fneur.2020.568536)

73. Robba C, Cardim D, Sekhon M, Budohoski K, Czosnyka M. Transcranial Doppler: a stethoscope for the brain-neurocritical care use. J Neuro Res. 2018 Apr;96(4):720–30. doi: [10.1002/jnr.24148](http://dx.doi.org/10.1002/jnr.24148)

74. Ross N, Eynon CA. Intracranial pressure monitoring. Current Anaesthesia & Critical Care. 2005 Jan;16(4):255–61. doi: [10.1016/j.cacc.2005.11.013](https://doi.org/10.1016/j.cacc.2005.11.013)

75. Sæhle T, Eide PK. Association between ventricular volume measures and pulsatile and static intracranial pressure scores in non-communicating hydrocephalus. Journal of the Neurological Sciences. 2015 Mar;350(1–2):33–9. doi: [10.1016/j.jns.2015.02.003](https://doi.org/10.1016/j.jns.2015.02.003)

76. Sahuquillo J, Rubio E, Codina A, Molins A, Guitart JM, Poca MA, et al. Reappraisal of the intracranial pressure and cerebrospinal fluid dynamics in patients with the so-called "Normal pressure hydrocephalus" syndrome. Acta neurochir. 1991 Mar;112(1–2):50–61. doi: 10.1007/BF01402454

77. Salci K, Enblad P, Piper I, Contant C, Nilsson P. A Model for Studies of Intracranial Volume Pressure Dynamics in Traumatic Brain Injury. Journal of Neurotrauma. 2004 Mar;21(3):317–27. doi: [10.1089/089771504322972103](https://doi.org/10.1089/089771504322972103)

78. Salci K, Nilsson P, Goiny M, Contant C, Piper I, Enblad P. Low Intracranial Compliance Increases the Impact of Intracranial Volume Insults to the Traumatized Brain. Neurosurgery. 2006 Aug 1;59(2):367–73. doi: [10.1227/01.NEU.0000222648.61065.38](https://doi.org/10.1227/01.NEU.0000222648.61065.38)

79. Schaller B, Graf R. Different Compartments of Intracranial Pressure and Its Relationship to Cerebral Blood Flow: The Journal of Trauma: Injury, Infection, and Critical Care. 2005 Dec;59(6):1521–31. doi: [10.1097/01.ta.0000197402.20180.6b](https://doi.org/10.1097/01.ta.0000197402.20180.6b)

80. Schummer W, Schummer C, Niesen W-D, Gerstenberg H. Doppler-guided cannulation of internal jugular vein, subclavian vein and innominate (brachiocephalic) vein—a case-control comparison in patients with reduced and normal intracranial compliance. Intensive Care Med. 2003 Sep;29(9):1535–40. doi: [10.1007/s00134-003-1862-4](https://doi.org/10.1007/s00134-003-1862-4)

81. Smith DW, Bailes JE, Fisher JA, Robles J, Turner RC, Mills JD. Internal Jugular Vein Compression Mitigates Traumatic Axonal Injury in a Rat Model by Reducing the Intracranial Slosh Effect: Neurosurgery. 2012 Mar;70(3):740–6. doi: [10.1227/NEU.0b013e318235b991](https://doi.org/10.1227/neu.0b013e318235b991)

82. Solheim O, Vik A, Gulati S, Eide PK. Rapid and severe rise in static and pulsatile intracranial pressures during a generalized epileptic seizure. Seizure. 2008 Dec;17(8):740–3. doi: [10.1016/j.seizure.2008.05.006](https://doi.org/10.1016/j.seizure.2008.05.006)

83. Sonnabend K, Brinker G, Maintz D, Bunck AC, Weiss K. Cerebrospinal fluid pulse wave velocity measurements: In vitro and in vivo evaluation of a novel multiband cine phase‐contrast MRI sequence. Magn Reson Med. 2021 Jan;85(1):197–208. doi: [10.1002/mrm.28430](https://doi.org/10.1002/mrm.28430)

84. Sood S, Kumar CR, Jamous M, Schuhmann MU, Ham SD, Canady AI. Pathophysiological changes in cerebrovascular distensibility in patients undergoing chronic shunt therapy. Journal of Neurosurgery: Pediatrics. 2004 May;100(5):447–53. doi: [10.3171/ped.2004.100.5.0447](https://doi.org/10.3171/ped.2004.100.5.0447)

85. Tain R-W, Alperin N. Compliance Effect on Amplitude and Phase of Cranio-Spinal CSF Flow Measured by MRI. International Conference on Bioinformatics and Biomedical Engineering. 2009:1–4. doi: [10.1109/ICBBE.2009.5162546](https://doi.org/10.1109/ICBBE.2009.5162546)

86. Tain R-W, Bagci AM, Lam BL, Sklar EM, Ertl-Wagner B, Alperin N. Determination of cranio-spinal canal compliance distribution by MRI: Methodology and early application in idiopathic intracranial hypertension. J Magn Reson Imaging. 2011 Dec;34(6):1397–404. doi: [10.1002/jmri.22799](https://doi.org/10.1002/jmri.22799)

87. Taylor GA, Phillips MD, Ichord RN, Carson BS, Gates JA, James CS. Intracranial Compliance in Infants: Evaluation with Doppler US. Pediatric Radiology. 1994;191:787–791. doi: [10.1148/radiology.191.3.8184065](https://doi.org/10.1148/radiology.191.3.8184065)

88. Timofeev I, Czosnyka M, Nortje J, Smielewski P, Kirkpatrick P, Gupta A, et al. Effect of decompressive craniectomy on intracranial pressure and cerebrospinal compensation following traumatic brain injury. JNS. 2008 Jan;108(1):66–73. doi: [10.3171/JNS/2008/108/01/0066](https://doi.org/10.3171/jns/2008/108/01/0066)

89. Tisell M, Stephensen H, Wikkelsø C. Elastance Correlates with Outcome after Endoscopic Third Ventriculostomy in Adults with Hydrocephalus Caused by Primary Aqueductal Stenosis. 2002;50(1):8. doi: [10.1097/00006123-200201000-00013](https://doi.org/10.1097/00006123-200201000-00013)

90. Wagshul ME, Eide PK, Madsen JR. The pulsating brain: A review of experimental and clinical studies of intracranial pulsatility. Fluids Barriers CNS. 2011 Dec;8(5):1–23. doi: [10.1186/2045-8118-8-5](https://doi.org/10.1186/2045-8118-8-5)

91. Svedung Wettervik T, Howells T, Enblad P, Lewén A. Intracranial pressure variability: relation to clinical outcome, intracranial pressure–volume index, cerebrovascular reactivity and blood pressure variability. J Clin Monit Comput. 2020 Aug;34(4):733–41. doi: [10.1007/s10877-019-00387-9](https://doi.org/10.1007/s10877-019-00387-9)

92. Wilkinson HA, Schuman N, Ruggiero J. Nonvolumetric methods of detecting impaired intracranial compliance or reactivity: Pulse width and wave form analysis. Journal of Neurosurgery. 1979 Jun;50(6):758–67. doi: [10.3171/jns.1979.50.6.0758](https://doi.org/10.3171/jns.1979.50.6.0758)

93. Wolf MS, Rakkar J, Horvat CM, Simon DW, Kochanek PM, Clermont G, et al. Assessment of Dynamic Intracranial Compliance in Children with Severe Traumatic Brain Injury: Proof-of-Concept. Neurocrit Care. 2021 Feb;34(1):209–17. doi: [10.1007/s12028-020-01004-3](https://doi.org/10.1007/s12028-020-01004-3)

94. Xu H, Fame RM, Sadegh C, Sutin J, Naranjo C, Della Syau, et al. Choroid plexus NKCC1 mediates cerebrospinal fluid clearance during mouse early postnatal development. Nat Commun. 2021 Dec;12(1):447. doi: [10.1038/s41467-020-20666-3](https://doi.org/10.1038/s41467-020-20666-3)

95. Yang YB, Sun B, Yang Z, Wang J, Pong Y. Effects of acute hypoxia on intracranial dynamics in unanesthetized goats. Journal of Applied Physiology. 1993 May 1;74(5):2067–71. doi: [10.1152/jappl.1993.74.5.2067](https://doi.org/10.1152/jappl.1993.74.5.2067)

96. Tirado-Caballero J, Rivero-Garvia M, Moreno-Madueño G, Gómez-González E, Márquez-Rivas J. Cranial expansion and aqueductoplasty for combined isolated fourth ventricle and slit-ventricle syndrome: a surgical alternative. Childs Nerv Syst. 2021 Mar;37(3):885–94. doi: [10.1007/s00381-020-04939-2](https://doi.org/10.1007/s00381-020-04939-2)

97. Shapiro K, Marmarou A, Shulman K. Characterization of clinical CSF dynamics and neural axis compliance using the pressure-volume index: I. The normal pressure-volume index. Ann Neurol. 1980 Jun;7(6):508–14. doi: [10.1002/ana.410070603](https://doi.org/10.1002/ana.410070603)

98. Lodi CA, Ursino M, Minassian AT, Beydon L. A Mathematical Model of Intracranial Pressure and Cerebral hemodynamics Response to CO_2_ changes. Transactions on Biomedicine and Health. 1997;4:101–11. doi: 10.2495/BIO970101

99. Qiu L, Xu L, Wang Y. Modeling of the Interaction between Intracranial Pressure and Cerebral Blood Flow. Third International Conference on Biomedical Engineering and Informatics. 2010:1217–20. doi: [10.1109/BMEI.2010.5639292](https://doi.org/10.1109/BMEI.2010.5639292)

100. Ursino M, Giulioni M, Lodi CA. Relationships among cerebral perfusion pressure, autoregulation, and transcranial Doppler waveform: a modeling study. Journal of Neurosurgery. 1998 Aug;89(2):255–66. doi: [10.3171/jns.1998.89.2.0255](https://doi.org/10.3171/jns.1998.89.2.0255)

101. Portella G, Cormio M, Citerio G. Continuous Cerebral Compliance Monitoring in Severe Head Injury: Its Relationship with Intracranial Pressure and Cerebral Perfusion Pressure. Acta Neurochir. 2002;81:173–5. doi: [10.1007/978-3-7091-6738-0_45](http://doi.org/10.1007/978-3-7091-6738-0_45)

102. Ursino M, Ter Minassian A, Lodi CA, Beydon L. Cerebral hemodynamics during arterial and CO _2_ pressure changes: in vivo prediction by a mathematical model. American Journal of Physiology-Heart and Circulatory Physiology. 2000 Nov 1;279(5):H2439–55. doi: [10.1152/ajpheart.2000.279.5.H2439](https://doi.org/10.1152/ajpheart.2000.279.5.h2439)

103. Kazimierska A, Kasprowicz M, Czosnyka M, Placek MM, Baledent O, Smielewski P, et al. Compliance of the cerebrospinal space: comparison of three methods. Acta Neurochir. 2021 Jul;163(7):1979–89. doi: [10.1007/s00701-021-04834-y](https://doi.org/10.1007/s00701-021-04834-y)
