# Supplementary material for: Intracranial Compliance Concepts and Assessment: A Scoping Review
Source: Front Neurol. 2021 Oct 25;12:756112. doi: 10.3389/fneur.2021.756112 (PMC8573119; doi:10.3389/fneur.2021.756112)
Supplement: Supplementary file 2 [file Table_2.docx]

Table 2: Most frequently methods and outcomes for measuring intracranial compliance.

| **Disease** | **Type of Method** | **Method** | **Outcome** |
| --- | --- | --- | --- |
| NPH | Invasive (1–30) | Intraventricular sensor (1,2,9,15,16,20–22,25,27,28,30) | CSF pulse pressure (1,15), PVI (2,16,20,28), ICP absolute value (1,9,20,21,25,28), CBF (20), b-wave (2,20,22), CSF resistance to outflow (1,20,22,28), compliance index (20,25), intracranial elastance (1,27), CSF outflow (2), PVR (25), ICP pulse amplitude (28,30), qualitative analysis of ICP waveform (30) |
|  |  | Lumbar puncture (8,9,12–15,17–20,24,29) | CSF pulse pressure (1), PVI (12,17,20), RAP (8), ICP absolute value (8,9,12,13,18–20,23,24,29), b-waves (8,20), CBF (20), CSF resistance to outflow (13,20), compliance index (20), CSF outflow conductance (24), ICP pulse amplitude (12,13,29), elastance coefficient (29), ICP pulse wave ratio P2/P1 (14) |
|  |  | Intraparenchymal sensor (3–8,10,11,23) | ICP absolute value (3,5,6,8,23), ICP pulse amplitude (3,4,7,11), ICP wave rise time (3), qualitative analysis of ICP waveform (6), RAP (8), b-waves (8), ICP pulsatility (5,10,23) |
|  |  | Extradural sensor (20) | CBF (20), ICP absolute value (20), b-wave (20), CSF resistance to outflow (20), PVI (20), compliance index (10) |
|  |  | Intrathecal sensor (26) | CSF outflow resistance (26), compliance index (26) |
|  | Non-invasive (4,10,14–16,18,20,25,31–39) | CT (15,16,18,20,35,37,38) | CBF pulsatility index (6), PVI (16), CSF resistance to outflow (18), elastance coefficient (18), Evans index (15,20), ventricular size (15,16,20,35), CSF flow (37), frontal horn ratio (38), CSF volume (38) |
|  |  | MRI (4,10,18,20,25,31–36,38,39) | CBF pulsatiliy index (31,32,35,36), delta-displacement analysis (32), resistance to CSF outflow (18), elastance coefficient (18), compliance index (25,33), Evans index (20), ventricular size (20,35), CSF pulse wave velocity (34), frontal horn ratio (38), CSF volume (38), CSF pressure gradient (25), PVR (25), CSF volumetric net flow rate (10), transfer function (39), CSF dynamics (4) |
|  |  | TCD (14) | CSF dynamics (14), cerebral blood volume (14) |
|  | Not reported (40) | Not reported (40) | CSF pulsatility (40) |
| HC | Invasive (41–72) | Intraventricular sensor (41,42,44,49,50,52–58,60,61,63,67–70,72) | RAP (53,56), PVI (44,49,50,53,70), CSF pressure (54), ICP absolute value (42,55,57–59,63,68), pulsatility index (56), CSF resistance to outflow (52,60,61,70), elastance index (59), VPR (49,50), ICP pulse amplitude (41,42,60,67), pressure-volume compensatory reserve (60), elastance coefficient (52,60), b-waves (63,72), compliance index (44,63), pressure-volume response (41), ICP pulse latency (42), ICP pulse slope (42), qualitative analysis of ICP waveform (72), plateau waves (72) |
|  |  | Lumbar puncture (42,46,53,59,60,62,66,67,69,70) | RAP (53), PVI (53,62,66,70), ICP absolute value (42,59,62), elastance index (59), CSF resistance to outflow (60,62,70), ICP pulse amplitude (42,46,60,62,66,67), pressure-volume compensatory reserve (60), elastance coefficient (60), ICP pulse latency (42), ICP pulse slope (42), CSF absorptive (69), elasticity slope (69) |
|  |  | Intraparenchymal sensor (47,48,51,65,71) | ICP absolute value (48,51,65), ICP pulse amplitude (48,51,65), ICP pulsatility (51), ICP wave rise time (51), ICP slow waves (65), RAP (65), transfer function (71), compliance slope (47), PVI (47) |
|  |  | Intratecal infusion test (45) | ICP absolute value (45) |
|  |  | Transcutaneous puncture of the subgaleal shunt reservoir (43) | ICP absolute value (43), ICP pulse amplitude (43) |
|  |  | Not reported (64) | PVI (64) |
|  | Noninvasive (45,46,55,57,61,73–76) | MRI (45,46,55,73,74) | CSF flow velocity (55), CSF pulsatility (45,74), CSF absorption (74), CSF flow (46,73), intracranial volume (46) |
|  |  | CT (45,74) | CSF pulsatility (45,74), CSF absorption (74) |
|  |  | TCD (57,61) | MCA blood flow velocity (57), pulsatility index (61), index of autoregulation (61) |
|  |  | TMD(75) | Tympanic membrane kinematic (75) |
|  |  | Cranial strain gauge sensor (76) | ICP pulse wave ratio P2/P1 (76) |
|  | Not reported (77–81) | Not reported (77–81) | CSF pulse pressure (77,80,81), ICP absolute value (77,78), PVI (79), CSF resistance to outflow (79), pulsatility index (80), resistive index (80,81), CSF pulsatility (81) |
| TBI | Invasive (50,82–146) | Intraventricular sensor (50,82–84,86,89–91,93,95,96,98,100,101,105–107,110,112,114,116,121–123,125–128,131,133,134,136,142,143,146) | ICP absolute value (82,84,86,89,96,101,105–107,112,122,123,125,146), PRx (95,98,112,114,121,136), qualitative analysis of ICP waveform (89,90,100,136), ICP high-frequency centroid (100), ICP pulse amplitude (83,95,101,122,128,131), ICP slow waves (95), RAP (101,112,116,122), VPR (50,84,143), PVI (50,89,90,105,107,110,126,133,142), CPP (106), elastance coefficient (107,146), compliance index (91,93,110,127,134), ICP pulse slope (122) |
|  |  | Intraparenchymal sensor (82,87,88,90,92,94–98,100,101,103,104,108,109,111,113–115,117–121,123,124,131,135–140,145) | ICP absolute value (82,87,96,97,101,103,104,123,124,139), ICP slow waves (94,95,97), CPP (97), RAP (97,101,103,104,111,115,117,118,145), PRx (92,94,95,98,104,111,114,115,118,121,136,139), qualitative analysis of ICP waveform (82,88,90,121,136), ICP pulse amplitude (92,95,101,103,109,118,131,137), ICP high-frequency centroid (100), plateau waves (104), CBF velocity (104), pulsatility index (104), index of autoregulation (104), RAP (94,108,113,120,135,138–140), ICP pulse wave ratio P2/P1 (108), compliance index (109,113), RAC (115), PVI (90), compensatory-reserve-weighted (140) |
|  |  | Subdural sensor (89,90,97,116,132,141,144) | ICP absolute value (89,97,132,144), ICP slow waves (97), CPP (97), RAP (97,116,141,144), qualitative analysis of ICP waveform (90), PVI (89,90), ICP pulse amplitude (89,141) |
|  |  | Extradural sensor (102) | ICP absolute value (102) |
|  |  | Subarachnoid sensor (82,90) | ICP absolute value (82), qualitative analysis of ICP waveform (82,90), PVI (90) |
|  |  | Epidural sensor (82,90,130) | ICP absolute value (82), qualitative analysis of ICP waveform (82,90), PVI (90,130) |
|  |  | Not reported (85,99,129) | ICP absolute value (99), CPP (99), PRx (85), RAP (85), RAC (85), b-waves (129) |
|  | Noninvasive (82,87,88,104,106,109,147–150) | TCD (82,87,104,106,109) | CBF (82,87), compliance index (87,106), PRx (87,104), MCA blood flow velocity (104), plateau waves (104), CBF velocity (104), qualitative analysis of ICP waveform (82,104), pulsatility index (104,109), index of autoregulation (104), RAP (104), intracranial elastance (82), elastance coefficient (106), PVI (106) |
|  |  | MRI (148,149) | MR-ICP (148), compliance index (149) |
|  |  | TMD (88) | ICP high-frequency centroid (88) |
|  |  | Cranial strain gauge sensor (82,150) | Qualitative analysis of ICP waveform (82), ICP pulse wave ratio P2/P1 (150) |
|  |  | Optic nerve sheath diameter (82) | Intracranial elastance (82) |
|  |  | Transcranial real-time sonography (147) | CSF displacement (147) |
|  | Not reported (151,152) | Not reported (151,152) | Finite element model (151), ICP absolute value (152), RAP (152), PRx (152) |
| SAH | Invasive (50,57,84,88,96,98,106,153–160) | Intraventricular sensor (50,57,84,96,98,106,153,156,157,159) | ICP absolute value (57,84,96,106,153,156,157,159), PVI (50), VPR (50,84), PRx (98), CPP (106,157), ICP pulse amplitude (153,156), ICP wave latency (156), compliance index (156,157) |
|  |  | Intraparenchymal sensor (88,96,98,154–156,158) | ICP absolute value (96,155,156,158), PRx (98), qualitative analysis of ICP waveform (88), ICP pulse amplitude (154–156,158), ICP wave latency (156), CPP (155), compliance index (156), ICP wave rise time (154), ICP wave rise time coefficient (154) |
|  |  | Not reported (160) | ICP absolute value (160), ICP pulse amplitude (160), CPP (160) |
|  | Noninvasive (39,57,88,106,157,161) | MRI (39) | Transfer function (39) |
|  |  | CT (161) | Ventricular dilatation (161), CSF flow (161) |
|  |  | TCD (57,106,157,161) | MCA blood flow velocity (57), compliance index (106), PVI (106), pulsatility index (106), CSF pulsatility (161), cerebral vasoreactivity (157), pressure autoregulatory response (157) |
|  |  | TMD (88) | ICP high-frequency centroid (88) |
|  | Not reported (162,163) | Not reported (162,163) | ICP absolute value (162,163), CBF (162), CPP (162) |
| ICH | Invasive (3,6,88,164–177) | Intraventricular sensor (170,174,175,177) | ICP absolute value (170,174,176,177), ICP pulse amplitude (176), VRP (175), cerebrovascular pressure transmission (177), cerebrovascular resistance (177), CPP (177) |
|  |  | Lumbar puncture (165,166,168,169,171,172) | PVI (165,168), elastance index (165), ICP absolute value (166,169,171), craniospinal elastance (168), CSF pressure (172), CSF pulse amplitude (172) |
|  |  | Intraparenchymal sensor (3,6,88,164,167,173) | ICP absolute value (3,6,173), ICP pulse amplitude (3,164,167), ICP wave rise time (3), ICP wave rise time coefficient (167), qualitative analysis of ICP waveform (6,88), CPP (173), PRx (173) |
|  | Noninvasive (88,174,178–181) | MRI (178,179) | PVI (178), CSF pressure (179), CSF resistance to outflow (179), production rate (179), elastance index (179) |
|  |  | CT (179) | CSF pressure (179), CSF resistance to outflow (179), production rate (179), elastance index (93) |
|  |  | TCD (180,181) | Venous flow velocity (180), elastance index (181), reserve capacity of craniovertebral contents (181) |
|  |  | TMD (88) | ICP high-frequency centroid (88) |
|  |  | US (174) | Optic nerve sheath diameter (174) |
| Brain tumor | Invasive (57,182–185) | Intraventricular sensor (57,182,185) | ICP absolute value (57,185), intraventricular pressure (182), PVI (185), VPR (185) |
|  |  | Subarachnoid sensor (184) | ICP absolute value (184), CPP (184) |
|  |  | Not reported (183) | ICP absolute value (183) |
|  | Noninvasive (57,181) | TCD (57,181) | MCA blood flow velocity (57), elastance index (181), reserve capacity of craniovertebral contents (181) |
| Stroke | Invasive (99,186,187) | Intraventricular sensor (187) | ICP absolute value (187), ICP pulse amplitude (187), RAP (187) |
|  |  | Intraparenchymal sensor (186,187) | ICP absolute value (186,187), ICP pulse amplitude (186,187), ICP slow waves (186), RAP (186,187) |
|  |  | Not reported (99) | ICP absolute value (99), CPP (99) |
|  | Not reported (188) | Not reported (188) | VPR (188) |
| Seizure | Invasive (189,190) | Intraventricular sensor (189,190) | ICP absolute value (189,190), ICP pulse amplitude (189,190) |
|  |  | Intraparenchymal sensor (190) | ICP absolute value (190), ICP pulse amplitude (190) |
| Chiari | Invasive (191,192) | Intraparenchymal sensor (191,192) | ICP pulsatility (191), ICP absolute value (192), ICP pulse amplitude (192) |
|  | Noninvasive (193–199) | MRI (193–199) | Compliance index (193–195,198,199), ICP pulse amplitude (196,197) |
| Intracranial hypotension | Noninvasive (200) | MRI (200) | CSF flow (200), intracranial volume change (200), CSF pressure gradient (200), intracranial elastance (200) |
|  | Not reported (201) | Not reported (201) | CSF reabsorption (201), ICP absolute value (201), plateau waves (201) |
| Intracranial aneurysms | Invasive (202) | Intraventricular sensor (202) | ICP absolute value (202), CBF (202), compliance index (202), VPR (202) |
| Slit ventricle syndrome | Invasive (203) | Intraventricular sensor (203) | ICP absolute value (203) |
|  | Noninvasive (204) | MRI (204) | Arterial inflow (204), venous outflow (204) |
| Comatose | Invasive (205) | Intraventricular sensor (205) | PVI (205) |
| CSF circulatory disorders | Invasive (206) | Epidural sensor (206) | Cerebrospinal elastance (206) |
| COVID-19 | Invasive (207) | Intraventricular sensor (207) | ICP absolute value (207), PRx (207), RAP (207), CPP (207), CSF (207) |
|  | Noninvasive (208) | Cranial strain gauge sensor (208) | ICP pulse wave ratio P2/P1 (208) |
|  |  | TCD (208) | Pulsatility index (208) |
| Diabetes | Noninvasive (209) | MRI (209) | Compliance index (209) |
|  |  | Brain compliance measurement device (209) | Compliance index (209) |
| Intra-abdominal hypertension | Not reported (210) | Not reported (210) | ICP absolute value (210), CPP (210) |
| Dialysis disequilibrium syndrome | Invasive (211) | Intraventricular sensor (211) | ICP absolute value (211) |
| Multiple sclerosis | Not reported (40) | Not reported (40) | CSF pulsatility (40) |
| Leukoaraiosis | Not reported (40) | Not reported (40) | CSF pulsatility (40) |
| Cerebral oedema | Not reported (151) | Not reported (151) | Finite element model (151) |
| Brain death | Invasive (212) | Not reported (212) | ICP pulse pressure (212), qualitative analysis of ICP waveform (212) |
| Chronic shunt therapy | Invasive (213) | Intraventricular sensor (213) | Compliance index (213), PVI (213) |
| Aqueductal obstruction | Invasive (214) | Intraventricular sensor (214) | CSF pressure (214) |
| Spinal block | Invasive (215,216) | Intraventricular sensor (216) | CSF pressure (216) |
|  |  | Lumbar puncture (215,216) | CSF pressure (215,216), CSF volume (215) |
|  |  | Cisterna magna puncture (216) | CSF pressure (216) |
| Mixed | Invasive (217–241) | Intraventricular sensor (218,220,221,225,227,229,233,234,237–239,241) | ICP absolute value (218,225,227,229,233,234), qualitative analysis of ICP waveform (218), CPP (218,233), ICP spectral amplitude (220), VPR (221), intracranial elastance (237), compliance index (238), ICP pulse amplitude (239,241), PVR (241) |
|  |  | Intraparenchymal sensor (218,222,225,231,232,236,239,240) | ICP absolute value (218,225,232,240), qualitative analysis of ICP waveform (218), ICP slow waves (231), RAP (231), PRx (231), CPP (218), RAP (236), cerebrospinal compensatory reserve (222), ICP pulse amplitude (239,240), ICP pulse latency (240) |
|  |  | Epidural sensor (218) | ICP absolute value (218), qualitative analysis of ICP waveform (218), CPP (218) |
|  |  | Subdural sensor (218,226) | ICP absolute value (218), qualitative analysis of ICP waveform (218), CPP (218), b-waves (226) |
|  |  | Subarachnoid sensor (218) | ICP absolute value (218), qualitative analysis of ICP waveform (218), CPP (218) |
|  |  | Extracranial sensor (218) | ICP absolute value (218), qualitative analysis of ICP waveform (218), CPP (218) |
|  |  | Cisterna magna puncture (228) | PVI (228) |
|  |  | Not reported (217,219,223,224,230,235) | ICP absolute value (217,224,230,235), qualitative analysis of ICP waveform (217,223,224,230,235), CPP (235), PVI (219,235), VPR (219,235), elastance index (235), ICP pulse wave P2 (219) |
|  | Noninvasive (224,225,229,230,242–250) | Cranial strain gauge sensor (245) | ICP pulse wave ratio P2/P1 (245) |
|  |  | MRI (230,242,244,248,249) | CBF (230), PVI (230), VRP (230), CSF (242), elastance index (242,248), CSF pulsation (248), compliance index (248), signal-void phenomenon (249), pulsatility index (244) |
|  |  | CT (224,230) | CBF (224,230), PVI (230), VRP (230), CPP (224), plateau waves (224), b-waves (224) |
|  |  | TCD (225,229,243–245,247,250) | Pulsatility index (229,243,245), CSF resistance to outflow (225), CBF (225), elastance coefficient (225), b-waves (247), pulsatility index (244,250), RAP (244), ICP pulse amplitude (244) |
|  |  | Fundoscopy (229) | Optic nerve sheath diameter (229) |
|  |  | TMD (229) | Tympanic membrane kinematic (229) |
|  |  | NIRS (230,245) | Cerebral oxygenation (230,245) |
|  |  | EEG (229) | Alpha-delta frequencies ratio (229) |
|  |  | Vittamed ultrasonic intracranial blood volume pulse wave monitor (246) | Area under ICP pulse wave (246) |
|  | Not reported (251–254) | Not reported (251–254) | ICP absolute value (251,254), compliance index (252), qualitative analysis of ICP waveform (252), ICP power spectrum (252), compliance slope (252), MCA blood flow velocity (253) |
| Children (HC) | Invasive (255–257) | Lumbar puncture (255) | CSF resistance to outflow (255), PVI (255), cerebrospinal compensatory reserve (255) |
|  |  | Intraparenchymal sensor (256,257) | ICP pulse amplitude (256), ICP pulse latency (256), RAP (257) |
|  | Noninvasive (258–262) | MRI (258,259) | ICP pulse amplitude (258,259), ICP wave rise time (258), ICP wave rise time coefficient (258) |
|  |  | TCD (260,261) | Resistance index (260,261) |
|  |  | Cranial strain gauge sensor (262) | ICP pulse wave ratio P2/P1 (262) |
| Children (TBI) | Invasive (263–265) | Intraventricular sensor (263,265) | RAP (263), ICP absolute value (265), ETC0_2_ (265) |
|  |  | Intraparenchymal sensor (263) | RAP (263) |
|  |  | Not reported (265) | ICP absolute value (264), ICP-PCO_2_ compliance index (265) |
|  | Noninvasive (264) | TCD (264) | MCA blood flow velocity (264) |
| Children (meningitis) | Noninvasive (266) | TCD (266) | Cerebral arteries and veins flow (266), resistive index |
| Children (acute subdural hematoma) | Noninvasive (267) | TCD (267) | Pulsatility index (267), resistivity index (267) |
| Children (craniosynostosis repair surgery) | Noninvasive (268) | TCD (268) | Resistivity index (268), pressure resistive index (268) |
| Children (craniostenosis) | Invasive (269) | Not reported (269) | RAP (269) |
| Children (mixed) | Noninvasive (270,271) | TCD (270,271) | Resistance index (270,271) |
| Children (myelodysplasia) | Invasive (272) | Intraventricular sensor (272) | ICP absolute value (272), ETC0_2_ (272) |
| Animal model (HC) | Invasive (47,273–279) | Intraventricular sensor (47,273,274,276–278) | ICP absolute value (47,273,274), CSF resistance to outflow (47), compliance index (276), ICP wave amplitude (276), PVR (277), PVI (278) |
|  |  | Intraparenchymal sensor (47) | ICP absolute value (47), CSF resistance to outflow (47) |
|  |  | Subdural sensor (275) | ICP absolute value (275) |
|  |  | Cisterna magna puncture (279) | Compliance coefficient (279) |
|  | Noninvasive (273) | MRI (273) | Evans index (273) |
| Animal model (TBI) | Invasive (280–284) | Intraventricular sensor (280–283) | ICP absolute value (280), PVI (281,282), compliance index (282,283) |
|  |  | Intraparenchymal sensor (284) | ICP absolute value (284) |
| Animal model (SAH) | Invasive (285) | Subdural sensor (285) | ICP absolute value (285) |
| Animal model (ICH) | Invasive (285–288) | Intraventricular sensor (286–288) | PVR (286,288), VPR (287) |
|  |  | Subdural sensor (285) | ICP absolute value (285) |
| Animal model (epidural hematoma) | Invasive (289) | Epidural sensor (289) | Brain eigenfrequency spectrum (289) |
| Animal model (brain edema) | Invasive (290–293) | Intraventricular sensor (290–293) | PVI (290–293), craniospinal compliance (291–293) |
| Animal model (acute hypoxia) | Invasive (294) | Intraventricular sensor (294) | ICP absolute value (294), CBF (294), compliance index (294), PVI (294) |
| Animal model (hemorrhagic shock) | Invasive (295) | Subdural sensor (295) | Intracranial elastance (295) |
| Animal model (cerebral venous congestion) | Invasive (296) | Intraventricular sensor (296) | VPR (296) |
| Animal model (brain compression) | Invasive (297) | Intraventricular sensor (297) | PVI (297) |
| Animal model (brain death) | Invasive (298) | Epidural sensor (298) | PVR (298) |

Abbreviations: ∆P, delta pressure; ∆V, delta volume; CBF, cerebral blood flow; CPP, cerebral perfusion pressure; CS, clinical study; CSF, cerebrospinal fluid; CT, computed tomography; EEG, electroencephalography; ETC02, end-tidal carbon dioxide; HC, hydrocephalus; ICH, intracranial hypertension; ICP, intracranial pressure; MCA, middle cerebral artery; Mixed, more than two neurological conditions; MM, mathematical model; MRI, magnetic resonance imaging; NIRS, near infrared spectroscopy; NPH, normal pressure hydrocephalus; P1, ICP first peak; P2, ICP second peak; PCO2, pressure of carbon dioxide; PRx, pressure reactivity index; PVI, pressure-volume index; PVR, pressure-volume response; RAC, ICP pulse amplitude and CPP correlation; RAP, compensatory reserve index; SAH, subarachnoid hemorrhage; TBI, traumatic brain injury; TCD, transcranial doppler ultrasonography; TMD, tympanic membrane displacement; US, ultrasonography; VPR, volume pressure response.

***References***

1. Anile C, De Bonis P, Albanese A, Di Chirico A, Mangiola A, Petrella G, et al. Selection of patients with idiopathic normal-pressure hydrocephalus for shunt placement: a single-institution experience: Clinical article. JNS. 2010 Jul;113(1):64–73. doi: [10.3171/2010.1.JNS091296](https://doi.org/10.3171/2010.1.JNS091296)

2. Børgesen SE, Gjerris F, Sørensen SC. Cerebrospinal fluid conductance and compliance of the craniospinal space in normal-pressure hydrocephalus: A comparison between two methods for measuring conductance to outflow. Journal of Neurosurgery. 1979 Oct;51(4):521–5. doi: [10.3171/jns.1979.51.4.0521](https://doi.org/10.3171/jns.1979.51.4.0521)

3. Eide PK, Eidsvaag VA, Hansson H-A. Antisecretory factor (AF) exerts no effects on intracranial pressure (ICP) waves and ICP in patients with idiopathic normal pressure hydrocephalus and idiopathic intracranial hypertension. Journal of the Neurological Sciences. 2014 Aug;343(1–2):132–7. doi: [10.1016/j.jns.2014.05.054](https://doi-org.ez31.periodicos.capes.gov.br/10.1016/j.jns.2014.05.054)

4. Eide PK, Pripp AH, Ringstad G. Magnetic resonance imaging biomarkers of cerebrospinal fluid tracer dynamics in idiopathic normal pressure hydrocephalus. Brain Communications. 2020 Jul 1;2(2):1–16. doi: [10.1093/braincomms/fcaa187](https://doi.org/10.1093/braincomms/fcaa187)

5. Eide PK. Cerebral microdialysis and intracranial pressure monitoring in patients with idiopathic normal-pressure hydrocephalus: association with clinical response to extended lumbar drainage and shunt surgery. J Neurosurg. 2010;112:11. doi: [10.3171/2009.5.JNS09122](https://doi.org/10.3171/2009.5.JNS09122)

6. Elixmann IM, Kwiecien M, Goffin C, Walter M, Misgeld B, Kiefer M, et al. Control of an Electromechanical Hydrocephalus Shunt—a New Approach. IEEE Trans Biomed Eng. 2014 Sep;61(9):2379–88. doi: [10.1109/TBME.2014.2308927](https://doi-org.ez31.periodicos.capes.gov.br/10.1109/TBME.2014.2308927)

7. Jacobsen HH, Sandell T, Jørstad ØK, Moe MC, Ringstad G, Eide PK. In Vivo Evidence for Impaired Glymphatic Function in the Visual Pathway of Patients With Normal Pressure Hydrocephalus. Invest Ophthalmol Vis Sci. 2020 Nov 17;61(13):24. doi: [10.1167/iovs.61.13.24](https://doi.org/10.1167/iovs.61.13.24)

8. Kim D-J, Czosnyka Z, Keong N, Radolovich DK, Smielewski P, Sutcliffe MPF, et al. Index of cerebrospinal compensatory reserve in hydrocephalus. Neurosurgery. 2009 Mar 1;64(3):494–502. doi: [10.1227/01.NEU.0000338434.59141.89](https://doi-org.ez31.periodicos.capes.gov.br/10.1227/01.NEU.0000338434.59141.89)

9. Lesniak MS, Clatterbuck RE, Rigamonti D, Williams MA. Low pressure hydrocephalus and ventriculomegaly: hysteresis, non-linear dynamics, and the benefits of CSF diversion. British Journal of Neurosurgery. 2002 Jan;16(6):555–61. doi: [10.1080/02688690209168360](https://doi-org.ez31.periodicos.capes.gov.br/10.1080/02688690209168360)

10. Lindstrøm EK, Ringstad G, Mardal K-A, Eide PK. Cerebrospinal fluid volumetric net flow rate and direction in idiopathic normal pressure hydrocephalus. NeuroImage: Clinical. 2018;20:731–41. doi: [10.1016/j.nicl.2018.09.006](https://doi-org.ez31.periodicos.capes.gov.br/10.1016/j.nicl.2018.09.006)

11. Park E-H, Eide PK, Zurakowski D, Madsen JR. Impaired pulsation absorber mechanism in idiopathic normal pressure hydrocephalus: Laboratory investigation. JNS. 2012 Dec;117(6):1189–96. doi: [10.3171/2012.9.JNS121227](https://doi.org/10.3171/2012.9.jns121227)

12. Qvarlander S, Lundkvist B, Koskinen L-OD, Malm J, Eklund A. Pulsatility in CSF dynamics: pathophysiology of idiopathic normal pressure hydrocephalus. Journal of Neurology, Neurosurgery & Psychiatry. 2013 Jul 1;84(7):735–41. doi: [10.1136/jnnp-2012-302924](http://dx.doi.org/10.1136/jnnp-2012-302924)

13. Qvarlander S, Malm J, Eklund A. CSF dynamic analysis of a predictive pulsatility-based infusion test for normal pressure hydrocephalus. Med Biol Eng Comput. 2014 Jan;52(1):75–85. doi: [10.1007/s11517-013-1110-1](https://doi.org/10.1007/s11517-013-1110-1)

14. Kazimierska A, Kasprowicz M, Czosnyka M, Placek MM, Baledent O, Smielewski P, et al. Compliance of the cerebrospinal space: comparison of three methods. Acta Neurochir. 2021 Jul;163(7):1979–89. doi: 10.1007/s00701-021-04834-y

15. Bárcena A, Mestre C, Cañizal JM, Rivero B, Lobato RD. Idiopathic normal pressure hydrocephalus: Analysis of factors related to cerebrospinal fluid dynamics determining functional prognosis. Acta neurochir. 1997 Oct;139(10):933–41. doi: 10.1007/BF01411302

16. Bergsneider M, Peacock WJ, Mazziotta JC, Becker DP. Beneficial Effect of Siphoning in Treatment of Adult Hydrocephalus. Arch Neurol. 1999 Oct 1;56(10):1224. doi: 10.1001/archneur.56.10.1224

17. Czosnyka Z, Owler B, Keong N, Santarius T, Baledent O, Pickard JD, et al. Impact of duration of symptoms on CSF dynamics in idiopathic normal pressure hydrocephalus: Duration of symptoms and CSF dynamics. Acta Neurologica Scandinavica. 2011 Jun;123(6):414–8. doi: 10.1111/j.1600-0404.2010.01420.x

18. Kasprowicz M, Czosnyka Z, Czosnyka M, Momjian S, Juniewicz H, Pickard JD. Slight elevation of baseline intracranial pressure after fluid infusion into CSF space in patients with hydrocephalus. Neurological Research. 2004 Sep;26(6):628–31. doi: 10.1179/016164104225017596

19. Lavinio A, Czosnyka Z, Czosnyka M. Cerebrospinal fluid dynamics: disturbances and diagnostics. European Journal of Anaesthesiology. 2008 Feb;25:137–41. doi: 10.1017/S0265021507003298

20. Sahuquillo J, Rubio E, Codina A, Molins A, Guitart JM, Poca MA, et al. Reappraisal of the intracranial pressure and cerebrospinal fluid dynamics in patients with the so-called "Normal pressure hydrocephalus" syndrome. Acta neurochir. 1991 Mar;112(1–2):50–61. doi: 10.1007/BF01402454

21. Tiefenthaler W, Burtscher J, Moser PL, Lorenz IH, Kolbitsch C. Intraventricular pressure in non-communicating hydrocephalus patients before endoscopic third ventriculostomy. Open Medicine. 2019 Nov 29;14(1):909–12. doi: 10.1515/med-2019-0107

22. Bech RA, Juhler M, Waldemar G, Klinken L, Gjerris F. Frontal Brain and Leptomeningeal Biopsy Specimens Correlated with Cerebrospinal Fluid Outflow Resistance and B-wave Activity in Patients Suspected of Normal-pressure Hydrocephalus. Neurosurgery. 1997 Mar 1;40(3):497–502. doi: 10.1097/00006123-199703000-00013

23. Eide PK, Hansson H-A. Astrogliosis and impaired aquaporin-4 and dystrophin systems in idiopathic normal pressure hydrocephalus. Neuropathol Appl Neurobiol. 2018 Aug;44(5):474–90. doi: 10.1111/nan.12420

24. Lundkvist B, Eklund A, Kristensen B, Fagerlund M, Koskinen L-OD, Malm J. Cerebrospinal fluid hydrodynamics after placement of a shunt with an antisiphon device: a long-term study. Journal of Neurosurgery. 2001 May;94(5):750–6. doi: 10.3171/jns.2001.94.5.0750

25. Mase M, Miyati T, Yamada K, Kasai H, Hara M, Shibamoto Y. Non-invasive measurement of intracranial compliance using cine MRI in normal pressure hydrocephalus. Acta Neurochirurgica Supplementum. 2005;95:303–6. doi: 10.1007/3-211-32318-x_62

26. Meier U. The Importance of the Intrathecal Infusion Test in the Diagnostics of Normal Pressure Hydrocephalus. Biomedical Engineering. 2001;46(7–8):191–9. doi: 10.1515/bmte.2001.46.7-8.191

27. Trevisi G, Signorelli F, de Waure C, Stifano V, Sturdà C, Rapisarda A, et al. Intraventricular infusion test accuracy in predicting short- and long-term outcome of iNPH patients: a 10-year update of a three-decade experience at a single institution. Neurosurg Rev. 2021 Feb 15. doi: 10.1007/s10143-021-01495-4

28. Kosteljanetz M. CSF dynamics and pressure-volume relationships in communicating hydrocephalus. J Neurosurg. 1986;64:45–52. doi: 10.3171/jns.1986.64.1.0045

29. Qvarlander S, Malm J, Eklund A. The pulsatility curve—the relationship between mean intracranial pressure and pulsation amplitude. Physiol Meas. 2010 Nov 1;31(11):1517–28. doi: 10.1088/0967-3334/31/11/008

30. Raftopoulos C, Chaskis C, Delecluse F, Cantrainet F, Bidauti L, Brotchi J. Morphological quantitative analysis of intracranial pressure waves in normal pressure hydrocephalus. Neurological Research. 1992 Jan;14(5):389–96. doi: 10.1080/01616412.1992.11740091

31. Bateman GA. The reversibility of reduced cortical vein compliance in normal-pressure hydrocephalus following shunt insertion. Neuroradiology. 2003 Feb;45(2):65–70. doi: 10.1007/s00234-002-0901-0

32. Kan H, Miyati T, Mase M, Osawa T, Ohno N, Kasai H, et al. Dynamic state of water molecular displacement of the brain during the cardiac cycle in idiopathic normal pressure hydrocephalus. Computerized Medical Imaging and Graphics. 2015 Mar;40:88–93. doi: 10.1016/j.compmedimag.2014.12.004

33. Miyati T, Mase M, Kasai H, Hara M, Yamada K, Shibamoto Y, et al. Noninvasive MRI assessment of intracranial compliance in idiopathic normal pressure hydrocephalus. J Magn Reson Imaging. 2007 Aug;26(2):274–8. doi: 10.1002/jmri.20999

34. Sonnabend K, Brinker G, Maintz D, Bunck AC, Weiss K. Cerebrospinal fluid pulse wave velocity measurements: In vitro and in vivo evaluation of a novel multiband cine phase‐contrast MRI sequence. Magn Reson Med. 2021 Jan;85(1):197–208. doi: 10.1002/mrm.28430

35. Bateman GA, Loiselle AM. Can MR measurement of intracranial hydrodynamics and compliance differentiate which patient with idiopathic normal pressure hydrocephalus will improve following shunt insertion? Acta Neurochir (Wien). 2007 May;149(5):455–62. doi: 10.1007/s00701-007-1142-0

36. Bateman G. Pulse-wave encephalopathy: a comparative study of the hydrodynamics of leukoaraiosis and normal-pressure hydrocephalus. Neuroradiology. 2002 Sep;44(9):740–8. doi: 10.1007/s00234-002-0812-0

37. Trinh VT, Duckworth EAM. Revision to an adjustable non-siphon control valve in low pressure hydrocephalus: Therapeutic siphoning and a new perspective on NPH. Clinical Neurology and Neurosurgery. 2013 Feb;115(2):175–8. doi: 10.1016/j.clineuro.2012.05.017

38. Houlden D, Khodorskiy D, Miller-Portman S, Li M. Importance of Frontal Horn Ratio and Optimal CSF Drainage in the Treatment of Very Low-Pressure Hydrocephalus. J Neurol Surg B. 2018 Aug;79(04):379–85. doi: 10.1055/s-0037-1609033

39. Miyati T, Mase M, Banno T, Kasuga T, Yamada K, Fujita H, et al. Frequency analyses of CSF flow on cine MRI in normal pressure hydrocephalus. Eur Radiol. 2003 May;13(5):1019–24. doi: 10.1007/s00330-002-1697-3

40. Beggs CB. Venous hemodynamics in neurological disorders: an analytical review with hydrodynamic analysis. BMC Med. 2013 Dec;11(1):142. doi: 10.1186/1741-7015-11-142

41. Eide PK. The correlation between pulsatile intracranial pressure and indices of intracranial pressure-volume reserve capacity: results from ventricular infusion testing. JNS. 2016 Dec;125(6):1493–503. doi: 10.3171/2015.11.JNS151529

42. Foltz EL, Blanks JP, Yonemura K. CSF pulsatility in hydrocephalus: respiratory effect on pulse wave slope as an indicator of intracranial compliance. Neurological Research. 1990 Jun;12(2):67–74. doi: [10.1080/01616412.1990.11739918](https://doi-org.ez31.periodicos.capes.gov.br/10.1080/01616412.1990.11739918)

43. Foltz EL, Blanks JP. Symptomatic low intracranial pressure in shunted hydrocephalus. J Neurosurg. 1988;68:401–8. doi: [10.3171/jns.1988.68.3.0401](https://doi.org/10.3171/jns.1988.68.3.0401)

44. González-Darder JM, Barcia-Salorio JL. Pulse Amplitude and Volume-pressure Relationships in Experimental Hydrocephalus. 1989;97:166–170. doi: 10.1007/BF01772830

45. Greitz D. Radiological assessment of hydrocephalus: new theories and implications for therapy. Neurosurg Rev. 2004;27:145–165. doi: [10.1007/s10143-004-0326-9](https://doi-org.ez31.periodicos.capes.gov.br/10.1007/s10143-004-0326-9)

46. Lokossou A, Balédent O, Garnotel S, Page G, Balardy L, Czosnyka Z, et al. ICP Monitoring and Phase-Contrast MRI to Investigate Intracranial Compliance. Intracranial Pressure & Neuromonitoring XVI, Acta Neurochirugica Supplement. 2018;126:247–53. doi: [10.1007/978-3-319-65798-1_50](https://doi.org/10.1007/978-3-319-65798-1_50)

47. Meier U, Kiefer M, Bartels P. The ICP-dependency of resistance to cerebrospinal fluid outflow: a new mathematical method for CSF-parameter calculation in a model with H-TX rats. Journal of Clinical Neuroscience. 2002 Jan;9(1):58–63. doi: [10.1054/jocn.2001.0930](https://doi.org/10.1054/jocn.2001.0930)

48. Park E-H, Dombrowski S, Luciano M, Zurakowski D, Madsen JR. Alterations of pulsation absorber characteristics in experimental hydrocephalus: Laboratory investigation. PED. 2010 Aug;6(2):159–70. doi: [10.1080/02688699943097](https://doi.org/10.1080/02688699943097)

49. Piper I, Spiegelberg A, Whittle I, Signorini D, Mascia L. A comparative study of the Spiegelberg Compliance Device with a manual volume-injection method: a clinical evaluation in patients with hydrocephalus. British Journal of Neurosurgery. 1999 Jan;13(6):581–6. doi: 10.1080/02688699943097

50. Raabe A, Czosnyka M, Piper I, Seifert V. Monitoring of Intracranial Compliance: Correction for a Change in Body Position. Acta Neurochirurgica. 1999 Jan 25;141(1):31–6. doi: [10.1007/s007010050263](https://doi.org/10.1007/s007010050263)

51. Sæhle T, Eide PK. Association between ventricular volume measures and pulsatile and static intracranial pressure scores in non-communicating hydrocephalus. Journal of the Neurological Sciences. 2015 Mar;350(1–2):33–9. doi: [10.1016/j.jns.2015.02.003](https://doi.org/10.1016/j.jns.2015.02.003)

52. Tisell M, Stephensen H, Wikkelsø C. Elastance Correlates with Outcome after Endoscopic Third Ventriculostomy in Adults with Hydrocephalus Caused by Primary Aqueductal Stenosis. 2002;50(1):8. doi: [10.1097/00006123-200201000-00013](https://doi.org/10.1097/00006123-200201000-00013)

53. Czosnyka M, Whitehouse H, Smielewski P, Simac S, Pickard JD. Testing of cerebrospinal compensatory reserve in shunted and non-shunted patients: a guide to interpretation based on an observational study. Journal of Neurology, Neurosurgery & Psychiatry. 1996 May 1;60(5):549–58. doi: 10.1136/jnnp.60.5.549

54. Daniel RT, Lee GYF, Halcrow SJ. Low-pressure Hydrocephalic State Complicating Hemispherectomy: A Case Report. Epilepsia. 2002 May 24;43(5):563–5. doi: 10.1046/j.1528-1157.2002.46101.x

55. Gholampour S, Bahmani M, Shariati A. Comparing the Efficiency of Two Treatment Methods of Hydrocephalus: Shunt Implantation and Endoscopic Third Ventriculostomy. Basic Clin Neurosci J. 2018 Oct 30;10(3):185–197. doi: 10.32598/bcn.9.10.285

56. Haubrich C, Czosnyka Z, Lavinio A, Smielewski P, Diehl RR, Pickard JD, et al. Is There a Direct Link Between Cerebrovascular Activity and Cerebrospinal Fluid Pressure-Volume Compensation? Stroke. 2007 Oct;38(10):2677–80. doi: 10.1161/STROKEAHA.107.485847

57. Kopniczky Z, Barzó P, Pávics L, Dóczi T, Bodosi M, Csernay L. Our policy in diagnosis and treatment of hydrocephalus. Child’s Nerv Syst. 1995 Feb;11(2):102–6. doi: 10.1007/BF00303814

58. Perrini P, Scollato A, Cioffi F, Mouchaty H, Conti R, Di Lorenzo N. Radiation leukoencephalopathy associated with moderate hydrocephalus: intracranial pressure monitoring and results of ventriculoperitoneal shunting. Neurological Sciences. 2002 Dec 1;23(5):237–41. doi: 10.1007/s100720200048

59. Piechnik SK, Ferreira VM, Cieslicki K. Estimation of cerebrospinal fluid compensation parameters in hydrocephalus using short-lasting constant rate lumbar infusion tests. British Journal of Neurosurgery. 2012 Feb;26(1):38–44. doi: 10.3109/02688697.2011.591848

60. Czosnyka M, Czosnyka ZH, Whitfield PC, Donovan T, Pickard JD. Age dependence of cerebrospinal pressure—volume compensation in patients with hydrocephalus. Journal of Neurosurgery. 2001 Mar;94(3):482–6. doi: 10.3171/jns.2001.94.3.0482

61. Czosnyka ZH, Czosnyka M, Whitfield PC, Donovan T, Pickard JD, Chir M. Cerebral Autoregulation among Patients with Symptoms of Hydrocephalus. Neurosurgery. 2002;50(3):526–33. doi: [10.1097/00006123-200203000-00018](https://doi.org/10.1097/00006123-200203000-00018)

62. Czosnyka Z, Czosnyka M, Owler B, Momjian S, Kasprowicz M, Schmidt EA, et al. Clinical testing of CSF circulation in hydrocephalus. Acta Neurochirurgica Supplementum. 2005;95:247–51. doi: [10.1007/3-211-32318-x_50](https://doi.org/10.1007/3-211-32318-x_50)

63. Yau YH, Piper IR, Contant CF, Dunn LT, Whittle IR. Clinical Experience in the use of the Spiegelberg Automated Compliance Device in the Assessment of Patients with Hydrocephalus. Acta Neurochirurgica Supplementum. 2002;81:171–2. doi: 10.1007/978-3-7091-6738-0_44

64. Lenfeldt N, Andersson N, Ågren-Wilsson A, Bergenheim AT, Koskinen L-OD, Eklund A, et al. Cerebrospinal fluid pulse pressure method: a possible substitute for the examination of B waves. Journal of Neurosurgery. 2004 Dec;101(6):944–50. doi: [10.3171/jns.2004.101.6.0944](https://doi.org/10.3171/jns.2004.101.6.0944)

65. Dias SF, Jehli E, Haas-Lude K, Bevot A, Okechi H, Zipfel J, et al. Ventriculomegaly in children: nocturnal ICP dynamics identify pressure-compensated but active paediatric hydrocephalus. Childs Nerv Syst. 2021 Jun;37(6):1883–93. doi: [10.1007/s00381-021-05164-1](https://doi.org/10.1007/s00381-021-05164-1)

66. Czosnyka M, Batorski L, Laniewski P, Maksymowicz W, Koszewski W, Zaworski W. A computer system for the identification of the cerebrospinal compensatory model. Acta neurochir. 1990 Sep;105(3–4):112–6. doi: [10.1007/BF01669992](https://doi.org/10.1007/bf01669992)

67. Czosnyka M, Czosnyka Z, Keong N, Lavinio A, Smielewski P, Momjian S, et al. Pulse pressure waveform in hydrocephalus: what it is and what it isn’t. FOC. 2007 Apr;22(4):1–7. doi: [10.3171/foc.2007.22.4.3](https://doi.org/10.3171/foc.2007.22.4.3)

68. Di Rocco C, Pettorossi VE, Caldarelli M, Mancinelli R, Velardi F. Communicating Hydrocephalus Induced by Mechanically Increased Amplitude of the Intraventricular Cerebrospinal Fluid Pressure: Experimental Studies. Experimental Neurology. 1978;59:40–52. doi: [10.1016/0014-4886(78)90199-1](https://doi.org/10.1016/0014-4886(78)90199-1)

69. Sklar FH, Diehl JT, Beyer CW, Clark WK. Brain elasticity changes with ventriculomegaly. Journal of Neurosurgery. 1980 Aug;53(2):173–9. doi: [10.3171/jns.1980.53.2.0173](https://doi.org/10.3171/jns.1980.53.2.0173)

70. Tans JTJ, Poortvliet DCJ. Relationship between compliance and resistance to outflow of CSF in adult hydrocephalus. J Neurosurg. 1989;71:59–62. doi: 10.3171/jns.1989.71.1.0059

71. Zou R, Park E-H, Kelly EM, Egnor M, Wagshul ME, Madsen JR. Intracranial pressure waves: characterization of a pulsation absorber with notch filter properties using systems analysis: Laboratory investigation. PED. 2008 Jul;2(1):83–94. doi: [10.3171/PED/2008/2/7/083](https://doi.org/10.3171/ped/2008/2/7/083)

72. Teichmann D, Lynch JC, Heldt T. Distortion of the Intracranial Pressure Waveform by Extraventricular Drainage System. IEEE Trans Biomed Eng. 2021 May;68(5):1646–57. doi: [10.1109/TBME.2020.3036283](https://doi.org/10.1109/tbme.2020.3036283)

73. Gholampour S, Fatouraee N. Boundary conditions investigation to improve computer simulation of cerebrospinal fluid dynamics in hydrocephalus patients. Commun Biol. 2021 Dec;4(1):394. doi: [10.1038/s42003-021-01920-w](https://doi.org/10.1038/s42003-021-01920-w)

74. Greitz D. Paradigm shift in hydrocephalus research in legacy of Dandy’s pioneering work: rationale for third ventriculostomy in communicating hydrocephalus. Childs Nerv Syst. 2007 Apr 5;23(5):487–9. doi: [10.1007/s00381-007-0303-z](https://doi.org/10.1007/s00381-007-0303-z)

75. Manwaring P, Wichern D, Manwaring M, Manwaring J, Manwaring K. A Signal Analysis Algorithm for Determining Brain Compliance Non-Invasively. Twenty-sixth Annual International Conference of the IEEE EMBS. 2004: 353–6. doi: [10.1109/ISSMD.2004.1689554](https://doi.org/10.1109/ISSMD.2004.1689554)

76. Ballestero MFM, Frigieri G, Cabella BCT, de Oliveira SM, de Oliveira RS. Prediction of intracranial hypertension through noninvasive intracranial pressure waveform analysis in pediatric hydrocephalus. Childs Nerv Syst. 2017 Sep;33(9):1517–24. doi: [10.1007/s00381-017-3475-1](https://doi.org/10.1007/s00381-017-3475-1)

77. Morandi X, Amlashi SFA, Riffaud L. A dynamic theory for hydrocephalus revealing benign intraspinal tumours: Tumoural obstruction of the spinal subarachnoid space reduces total CSF compartment compliance. Medical Hypotheses. 2006 Jan;67(1):79–81. doi: [10.1016/j.mehy.2006.01.005](https://doi.org/10.1016/j.mehy.2006.01.005)

78. Salman MS. Benign intracranial hypertension or communicating hydrocephalus: factors in pathogenesis. Medical Hypotheses. 1997 Nov;49(5):371–3. doi: [10.1016/S0306-9877(97)90081-5](https://doi.org/10.1016/S0306-9877(97)90081-5)

79. Jaeger KM, Layton TN. Hydrodynamic principles in hydrocephalus The engineer’s perspective. Neurological Research. 2000 Jan;22(1):97–101. doi: [10.1080/01616412.2000.11741042](https://doi.org/10.1080/01616412.2000.11741042)

80. Egnor M, Zheng L, Rosiello A, Gutman F, Davis R. A Model of Pulsations in Communicating Hydrocephalus. Pediatr Neurosurg. 2002;36(6):281–303. doi: [10.1159/000063533](https://doi.org/10.1159/000063533)

81. Egnor M, Rosiello A, Zheng L. A Model of Intracranial Pulsations. Pediatr Neurosurg. 2001;35(6):284–98. doi: [10.1159/000050440](https://doi.org/10.1159/000050440)

82. Heldt T, Zoerle T, Teichmann D, Stocchetti N. Intracranial Pressure and Intracranial Elastance Monitoring in Neurocritical Care. Annu Rev Biomed Eng. 2019 Jun 4;21(1):523–49. doi: [10.1146/annurev-bioeng-060418-052257](https://doi.org/10.1146/annurev-bioeng-060418-052257)

83. Behzadnia H, Emamhadi M, Chabok SY, Alijani B, Jafari S, Andalib S. The Correlation Between Intracranial Pressure Amplitude and Glasgow Outcome Scale in Patients with Traumatic Brain Injury. Trauma Mon. 2018 Nov;23(6):1–5. doi: 10.5812/TRAUMAMON.79490

84. Burchiel KJ, Steege TD, Wyler AR. Intracranial Pressure Changes in Brain-injured Patients Requiring Positive End-Expiratory Pressure Ventilation. Neurosurgery. 1981;8(4):443–9. doi: [10.1227/00006123-198104000-00007](https://doi.org/10.1227/00006123-198104000-00007)

85. Jha RM, Kochanek PM. A Precision Medicine Approach to Cerebral Edema and Intracranial Hypertension after Severe Traumatic Brain Injury: Quo Vadis? Curr Neurol Neurosci Rep. 2018 Dec;18(12):105. doi: [10.1007/s11910-018-0912-9](https://dx.doi.org/10.1007%2Fs11910-018-0912-9)

86. Kiening K, Schoening W, Stover J, Unterberg A. Continuous monitoring of intracranial compliance after severe head injury: relation to data quality, intracranial pressure and brain tissue PO _2_. British Journal of Neurosurgery. 2003 Jan;17(4):311–8. doi: [10.1080/02688690310001601199](https://doi.org/10.1080/02688690310001601199)

87. Kim D-J, Czosnyka Z, Kasprowicz M, Smieleweski P, Baledent O, Guerguerian A-M, et al. Continuous Monitoring of the Monro-Kellie Doctrine: Is It Possible? Journal of Neurotrauma. 2012 May;29(7):1354–63. doi: [10.1089/neu.2011.2018](https://doi.org/10.1089/neu.2011.2018)

88. Lang EW, Paulat K, Witte C, Zolondz J, Mehdorn HM. Noninvasive intracranial compliance monitoring: Technical note and clinical results. Journal of Neurosurgery. 2003 Jan;98(1):214–8. doi: [10.3171/jns.2003.98.1.0214](https://doi.org/10.3171/jns.2003.98.1.0214)

89. Lewis S. Cerebrovascular pressure transmission analysis as a guide to the pathophysiology of raised intracranial pressure. Clin Exp Pharmacol Physiol. 1998 Nov;25(11):947–50. doi: [10.1111/j.1440-1681.1998.tb02350.x](https://doi.org/10.1111/j.1440-1681.1998.tb02350.x)

90. March K. Intracranial Pressure Monitoring and Assessing Intracranial Compliance in Brain Injury. Critical Care Nursing Clinics of North America. 2000 Dec;12(4):429–36. doi: [10.1016/S0899-5885(18)30079-0](https://doi.org/10.1016/S0899-5885(18)30079-0)

91. Nujaimin U, Saufi A, A. Rahman G, Badrisyah I, Sani S, Zamzuri I, et al. Post Traumatic Cerebral Oedema in Severe Head Injury is Related to Intracranial Pressure and Cerebral Perfusion Pressure but not to Cerebral Compliance. Asian Journal of Surgery. 2009 Jul;32(3):157–62. doi: [10.1016/s1015-9584(09)60387-0](https://doi.org/10.1016/s1015-9584(09)60387-0)

92. Nyholm L, Howells T, Enblad P. Predictive Factors That May Contribute to Secondary Insults With Nursing Interventions in Adults With Traumatic Brain Injury. Journal of Neuroscience Nursing. 2017 Feb;49(1):49–55. doi: [10.1097/JNN.0000000000000260](https://doi.org/10.1097/jnn.0000000000000260)

93. Portella G, Cormio M, Citerio G, Contant C, Kiening K, Enblad P, et al. Continuous cerebral compliance monitoring in severe head injury: its relationship with intracranial pressure and cerebral perfusion pressure. Acta Neurochir (Wien). 2005 Jul;147(7):707–13. doi: [10.1007/s00701-005-0537-z](https://doi.org/10.1007/s00701-005-0537-z)

94. Timofeev I, Czosnyka M, Nortje J, Smielewski P, Kirkpatrick P, Gupta A, et al. Effect of decompressive craniectomy on intracranial pressure and cerebrospinal compensation following traumatic brain injury. JNS. 2008 Jan;108(1):66–73. doi: [10.3171/JNS/2008/108/01/0066](https://doi.org/10.3171/jns/2008/108/01/0066)

95. Svedung Wettervik T, Howells T, Enblad P, Lewén A. Intracranial pressure variability: relation to clinical outcome, intracranial pressure–volume index, cerebrovascular reactivity and blood pressure variability. J Clin Monit Comput. 2020 Aug;34(4):733–41. doi: [10.1007/s10877-019-00387-9](https://doi.org/10.1007/s10877-019-00387-9)

96. Fletcher JJ, Bergman K, Carlson G, Feucht EC, Blostein PA. Continuous Renal Replacement Therapy for Refractory Intracranial Hypertension? Journal of Trauma: Injury, Infection & Critical Care. 2010 Jun;68(6):1506–9. doi: [10.1097/TA.0b013e3181dbbf1b](https://doi.org/10.1097/ta.0b013e3181dbbf1b)

97. Whitfield PC, Patel H, Hutchinson PJA, Czosnyka M, Parry D, Menon D, et al. Bifrontal decompressive craniectomy in the management of posttraumatic intracranial hypertension. British Journal of Neurosurgery. 2001 Jan;15(6):500–7. doi: [10.1080/02688690120105110](https://doi.org/10.1080/02688690120105110)

98. Proescholdt MA, Faltermeier R, Bele S, Brawanski A. Detection of Impaired Cerebral Autoregulation Using Selected Correlation Analysis: A Validation Study. Computational and Mathematical Methods in Medicine. 2017;2017:1–7. doi: [10.1155/2017/8454527](https://doi.org/10.1155/2017/8454527)

99. Faltermeier R, Proescholdt MA, Brawanski A. Computerized Data Analysis of Neuromonitoring Parameters Identifies Patients with Reduced Cerebral Compliance as Seen on CT. Acta Neurochirurgica Supplementum. 2012;114:35–8. doi: [10.1007/978-3-7091-0956-4_7](https://doi.org/10.1007/978-3-7091-0956-4_7)

100. Robertson CS, Narayan RK, Contant CF, Grossman RG, Gokaslan ZL, Pahwa R, et al. Clinical experience with a continuous monitor of intracranial compliance. Journal of Neurosurgery. 1989 Nov;71(5):673–80. doi: [10.3171/jns.1989.71.5.0673](https://doi.org/10.3171/jns.1989.71.5.0673)

101. Hall A, O’Kane R. The best marker for guiding the clinical management of patients with raised intracranial pressure—the RAP index or the mean pulse amplitude? Acta Neurochir. 2016 Oct;158(10):1997–2009. doi: [10.1007/s00701-016-2932-z](https://doi.org/10.1007/s00701-016-2932-z)

102. Hilt H, Gramm H-J, Link J. Changes in intracranial pressure associated with extradural anaesthesia. British Journal of Anaesthesia. 1986 Jun;58(6):676–80. doi: [10.1093/bja/58.6.676](https://doi.org/10.1093/bja/58.6.676)

103. Steiner LA, Balestreri M, Johnston AJ, Coles JP, Smielewski P, Pickard JD, et al. Predicting the response of intracranial pressure to moderate hyperventilation. Acta Neurochir (Wien). 2005 May;147(5):477–83. doi: [10.1007/s00701-005-0510-x](https://doi.org/10.1007/s00701-005-0510-x)

104. Czosnyka M, Smielewski P, Piechnik S, Schmidt EA, Al-Rawi PG, Kirkpatrick PJ, et al. Hemodynamic characterization of intracranial pressure plateau waves in head-injured patients. Journal of Neurosurgery. 1999 Jul;91(1):11–9. doi: [10.3171/jns.1999.91.1.0011](https://doi.org/10.3171/jns.1999.91.1.0011)

105. Frost EM. Effects of positive end-expiratory pressure on intracranial pressure and compliance in brain-injured patients. J Neurosurg. 1977;47:195–200. doi: [10.3171/jns.1977.47.2.0195](https://doi.org/10.3171/jns.1977.47.2.0195)

106. Caricato A, Conti G, Corte FD, Mancino A, Santilli F, Sandroni C, et al. Effects of PEEP on the Intracranial System of Patients With Head Injury and Subarachnoid Hemorrhage: The Role of Respiratory System Compliance. The Journal of Trauma: Injury, Infection, and Critical Care. 2005 Mar;58(3):571–6. doi: [10.1097/01.ta.0000152806.19198.db](https://doi.org/10.1097/01.ta.0000152806.19198.db)

107. Ursino M, Lodi CA, Rossi S, Stocchetti N. Intracranial pressure dynamics in patients with acute brain damage. Journal of Applied Physiology. 1997 Apr 1;82(4):1270–82. doi: [10.1152/jappl.1997.82.4.1270](https://doi.org/10.1152/jappl.1997.82.4.1270)

108. Dias C, Maia I, Cerejo A, Varsos G, Smielewski P, Paiva J-A, et al. Pressures, Flow, and Brain Oxygenation During Plateau Waves of Intracranial Pressure. Neurocrit Care. 2014 Aug;21(1):124–32. doi: [10.1007/s12028-013-9918-y](https://doi.org/10.1007/s12028-013-9918-y)

109. Kim D-J, Kasprowicz M, Carrera E, Castellani G, Zweifel C, Lavinio A, et al. The monitoring of relative changes in compartmental compliances of brain. Physiol Meas. 2009 Jul 1;30(7):647–59. doi: [10.1088/0967-3334/30/7/009](https://doi.org/10.1088/0967-3334/30/7/009)

110. Abdullah J, Zamzuri I, Awang S, Sayuthi S, Ghani A, Tahir A, et al. Preliminary report on spiegelberg pre and post-operative monitoring of severe head-injured patients who received decompressive craniectomy. Acta Neurochirurgica Supplementum. 2005;95:311–4. doi: [10.1007/3-211-32318-x_64](https://doi.org/10.1007/3-211-32318-x_64)

111. Balestreri M, Czosnyka M, Steiner LA, Schmidt E, Smielewski P, Matta B, et al. Intracranial hypertension: what additional information can be derived from ICP waveform after head injury? Acta Neurochir. 2004 Feb;146(2):131–41. doi: [10.1136/jnnp.2003.033126](http://dx.doi.org/10.1136/jnnp.2003.033126)

112. Bennis FC, Teeuwen B, Zeiler FA, Elting JW, van der Naalt J, Bonizzi P, et al. Improving Prediction of Favourable Outcome After 6 Months in Patients with Severe Traumatic Brain Injury Using Physiological Cerebral Parameters in a Multivariable Logistic Regression Model. Neurocrit Care. 2020 Oct;33(2):542–51. doi: [10.1007/s12028-020-00930-6](https://doi.org/10.1007/s12028-020-00930-6)

113. Beqiri E, Czosnyka M, Lalou AD, Zeiler FA, Fedriga M, Steiner LA, et al. Influence of mild-moderate hypocapnia on intracranial pressure slow waves activity in TBI. Acta Neurochir. 2020 Feb;162(2):345–56. doi: [10.1007/s00701-019-04118-6](https://doi.org/10.1007/s00701-019-04118-6)

114. Bor-Seng-Shu E, Figueiredo EG, Fonoff ET, Fujimoto Y, Panerai RB, Teixeira MJ. Decompressive craniectomy and head injury: brain morphometry, ICP, cerebral hemodynamics, cerebral microvascular reactivity, and neurochemistry. Neurosurg Rev. 2013 Jul;36(3):361–70. doi: [10.1007/s10143-013-0453-2](https://doi.org/10.1007/s10143-013-0453-2)

115. Castellani G, Zweifel C, Kim D-J, Carrera E, Radolovich DK, Smielewski P, et al. Plateau Waves in Head Injured Patients Requiring Neurocritical Care. Neurocrit Care. 2009 Oct;11(2):143–50. doi: [10.1007/s12028-009-9235-7](https://doi.org/10.1007/s12028-009-9235-7)

116. Czosnyka M, Price DJ, Williamson M. Monitoring of cerebrospinal dynamics using continuous analysis of intracranial pressure and cerebral perfusion pressure in head injury. Acta neurochir. 1994 Jun;126(2–4):113–9. doi: [10.1007/BF01476419](https://doi.org/10.1007/bf01476419)

117. Czosnyka M, Steiner L, Balestreri M, Schmidt E, Smielewski P, Hutchinson PJ, et al. Concept of “true ICP” in monitoring and prognostication in head trauma. Acta Neurochirurgica Supplementum. 2005;95:341–4. doi: [10.1007/3-211-32318-x_70](https://doi.org/10.1007/3-211-32318-x_70)

118. Donnelly J, Smielewski P, Adams H, Zeiler FA, Cardim D, Liu X, et al. Observations on the Cerebral Effects of Refractory Intracranial Hypertension After Severe Traumatic Brain Injury. Neurocrit Care. 2020 Apr;32(2):437–47. doi: [10.1007/s12028-019-00748-x](https://doi.org/10.1007/s12028-019-00748-x)

119. Hall CA. Patient management in head injury care: a nursing perspective. Intensive and Critical Care Nursing. 1997;13:329–37. doi: [10.1016/s0964-3397(97)81076-2](https://doi.org/10.1016/s0964-3397(97)81076-2)

120. Haubrich C, Steiner LA, Diehl RR, Kasprowicz M, Smielewski P, Pickard JD, et al. Doppler Flow Velocity and Intra-cranial Pressure: Responses to Short-Term Mild Hypocapnia Help to Assess the Pressure-Volume Relationship After Head Injury. Ultrasound in Medicine & Biology. 2013 Sep;39(9):1521–6. doi: [10.1016/j.ultrasmedbio.2013.03.020](https://doi.org/10.1016/j.ultrasmedbio.2013.03.020)

121. Hawthorne C, Piper I. Monitoring of Intracranial Pressure in Patients with Traumatic Brain Injury. Front Neurol. 2014 Jul 16;5:1–16. doi: [10.3389/fneur.2014.00121](https://dx.doi.org/10.3389%2Ffneur.2014.00121)

122. Howells T, Lewén A, Sköld MK, Ronne-Engström E, Enblad P. An evaluation of three measures of intracranial compliance in traumatic brain injury patients. Intensive Care Med. 2012 Jun;38(6):1061–8. doi: [10.1007/s00134-012-2571-7](https://doi.org/10.1007/s00134-012-2571-7)

123. Jha RM, Elmer J, Zusman BE, Desai S, Puccio AM, Okonkwo DO, et al. Intracranial Pressure Trajectories: A Novel Approach to Informing Severe Traumatic Brain Injury Phenotypes*. Critical Care Medicine. 2018 Nov;46(11):1792–802. doi: [10.1097/CCM.0000000000003361](https://doi.org/10.1097/ccm.0000000000003361)

124. Kayani AS, Feldman JP. Prone ventilation in a patient with traumatic brain injury, bifrontal craniectomy and intracranial hypertension. Trauma. 2015 Jul;17(3):224–8. doi: [10.1177/1460408614557857](https://doi.org/10.1177%2F1460408614557857)

125. Kerwin AJ, Croce MA, Timmons SD, Maxwell RA, Malhotra AK, Fabian TC. Effects of Fiberoptic Bronchoscopy on Intracranial Pressure in Patients with Brain Injury: A Prospective Clinical Study: The Journal of Trauma: Injury, Infection, and Critical Care. 2000 May;48(5):878–83. doi: [10.1097/00005373-200005000-00011](https://doi.org/10.1097/00005373-200005000-00011)

126. Kiening KL, Schoening WN, Lanksch WR, Unterberg AW. Intracranial Compliance as a Bed-Side Monitoring Technique in Severely Head-Injured Patients. Acta Neurochir. 2002;81:177–80. doi: [10.1007/978-3-7091-6738-0_46](https://doi.org/10.1007/978-3-7091-6738-0_46)

127. Kiening KL, Schoening W, Unterberg AW, Stover JF, Citerio G, et al. Assessment of the relationship between age and continuous intracranial compliance. Acta Neurochirurgica Supplementum. 2005;95:293–7. doi: [10.1007/3-211-32318-x_60](https://doi.org/10.1007/3-211-32318-x_60)

128. Lalou AD, Levrini V, Czosnyka M, Gergelé L, Garnett M, Kolias A, et al. Cerebrospinal fluid dynamics in non-acute post-traumatic ventriculomegaly. Fluids Barriers CNS. 2020 Dec;17(1):24. doi: [10.1186/s12987-020-00184-6](https://doi.org/10.1186/s12987-020-00184-6)

129. Lemaire JJ, Khalil T, Cervenansky F, Gindre G, Boire JY, Bazin JE, et al. Slow Pressure Waves in the Cranial Enclosure. Acta Neurochir (Wien). 2002;144:243–54. doi: [10.1007/s007010200032](https://doi.org/10.1007/s007010200032)

130. Ludwig HC, Klingler M, Timmermann A, Weyland W, Mursch K, Reparon C, Markakis E. The Influence of Airway Pressure Changes on Intracranial Pressure (ICP) and the Blood Flow Velocity in the Middle Cerebral Artery (V_MCA_). Anästhesiol Intensivmed Notfallmed Schmerzther. 2005;35:141–5. doi: [10.1055/s-2000-13008](https://doi.org/10.1055/s-2000-13008)

131. Nyholm L, Howells T, Lewén A, Hillered L, Enblad P. The influence of hyperthermia on intracranial pressure, cerebral oximetry and cerebral metabolism in traumatic brain injury. Upsala Journal of Medical Sciences. 2017 Jul 3;122(3):177–84. doi: [10.1080/03009734.2017.1319440](https://doi.org/10.1080/03009734.2017.1319440)

132. Panerai RB, Hudson V, Fan L, Mahony P, Yeoman PM, Hope T, et al. Assessment of dynamic cerebral autoregulation based on spontaneous fluctuations in arterial blood pressure and intracranial pressure. Physiol Meas. 2002 Feb 1;23(1):59–72. doi: [10.1088/0967-3334/23/1/306](https://doi.org/10.1088/0967-3334/23/1/306)

133. Pillai S, Praharaj S, Rao G, Kolluri V. Cerebral perfusion pressure management of severe diffuse head injury: Effect on brain compliance and intracranial pressure. Neurology India. 2004;25(1):67–71. Available from: <https://www.neurologyindia.com/text.asp?2004/52/1/67/6701>

134. Salci K, Nilsson P, Howells T, Ronne-Engström E, Piper I, Contant CF, et al. Intracerebral Microdialysis and Intracranial Compliance Monitoring of Patients with Traumatic Brain Injury. J Clin Monit Comput. 2006 Feb;20(1):25–31. doi: [10.1007/s10877-006-2864-x](https://doi.org/10.1007/s10877-006-2864-x)

135. Timofeev I, Dahyot-Fizelier C, Keong N, Nortje J, Al-Rawi PG, Czosnyka M, et al. Ventriculostomy for control of raised ICP in acute traumatic brain injury. Acta Neurochirurgica Supplementum. 2008;102:99–104. doi: [10.1007/978-3-211-85578-2_20](https://doi.org/10.1007/978-3-211-85578-2_20)

136. Vidgeon SD, Strong AJ. Multimodal Cerebral Monitoring in Traumatic Brain Injury. Journal of the Intensive Care Society. 2011 Apr;12(2):126–33. doi: [10.1177/175114371101200208](https://doi.org/10.1177%2F175114371101200208)

137. Westhout FD, Paré LS, Delfino RJ, Cramer SC. Slope of the intracranial pressure waveform after traumatic brain injury. Surgical Neurology. 2008 Jul;70(1):70–4. doi: [10.1016/j.surneu.2007.04.020](https://doi.org/10.1016/j.surneu.2007.04.020)

138. Zeiler FA, Kim D-J, Cabeleira M, Calviello L, Smielewski P, Czosnyka M. Impaired cerebral compensatory reserve is associated with admission imaging characteristics of diffuse insult in traumatic brain injury. Acta Neurochir. 2018 Dec;160(12):2277–87. doi: [10.1007/s00701-018-3681-y](https://doi.org/10.1007/s00701-018-3681-y)

139. Zeiler FA, Donnelly J, Menon DK, Smielewski P, Hutchinson PJA, Czosnyka M. A Description of a New Continuous Physiological Index in Traumatic Brain Injury Using the Correlation between Pulse Amplitude of Intracranial Pressure and Cerebral Perfusion Pressure. Journal of Neurotrauma. 2018 Apr;35(7):963–74. doi: [10.1089/neu.2017.5241](https://doi.org/10.1089/neu.2017.5241)

140. Zeiler FA, Ercole A, Cabeleira M, Beqiri E, Zoerle T, et al. Compensatory-reserve-weighted intracranial pressure versus intracranial pressure for outcome association in adult traumatic brain injury: a CENTER-TBI validation study. Acta Neurochir. 2019 Jul;161(7):1275–84. doi: 10.1007/s00701-019-03915-3

141. Czosnyka M, Guazzo E, Whitehouse M, Smielewski P, Czosnyka Z, Kirkpatrick P, et al. Significance of intracranial pressure waveform analysis after head injury. Acta neurochir. 1996 May;138(5):531–42. doi: [10.1007/BF01411173](https://doi.org/10.1007/bf01411173)

142. Ursino M, Lodi A, Rossi S, Stocchetti N. Estimation of the Main Factors Affecting ICP Dynamics by Mathematical Analysis of PVI Test. Acta Neurochirurgica Supplementum. 1998;71:306–9. doi: 10.1007/978-3-7091-6475-4_89

143. Portella G, Cormio M, Citerio G. Continuous cerebral compliance monitoring in severe head injury: its relationship with intracranial pressure and cerebral perfusion pressure. Acta Neurochir (Wien). 2005 Jul;147(7):707–13. doi: [10.1007/s00701-005-0537-z](https://doi.org/10.1007/s00701-005-0537-z)

144. Czosnyka M, Whitehouse H, Smielewski P, Kirkpatrick P, Guazzo EP, Pickard JD. Computer supported multimodal bed-side monitoring for neuro intensive care. J Clin Monit Comput. 1994 Nov;11(4):223–32. doi: [10.1007/BF01139874](https://doi.org/10.1007/bf01139874)

145. Highton D, Panovska-Griffiths J, Smith M, Elwell CE. Mathematical Modelling of Near-Infrared Spectroscopy Signals and Intracranial Pressure in Brain-Injured Patients. Advances in Experimental Medicine and Biology, 2013;789:345–51. doi: [10.1007/978-1-4614-7411-1_46](https://doi.org/10.1007/978-1-4614-7411-1_46)

146. Ursino M, Ter Minassian A, Lodi CA, Beydon L. Cerebral hemodynamics during arterial and CO _2_ pressure changes: in vivo prediction by a mathematical model. American Journal of Physiology-Heart and Circulatory Physiology. 2000 Nov 1;279(5):H2439–55. doi: [10.1152/ajpheart.2000.279.5.H2439](https://doi.org/10.1152/ajpheart.2000.279.5.h2439)

147. Mursch K, Vogelsang JP, Zimmerer B, Ludwig HC, Behnke J, Markakis E. Bedside measurement of the third ventricle’s diameter during episodes of arising intracranial pressure after head trauma: Using transcranial real-time sonography for a non-invasive examination of intracranial compensation mechanisms. Acta neurochir. 1995 Mar;137(1–2):19–24. doi: [10.1007/BF02188774](https://doi.org/10.1007/bf02188774)

148. Burman R, Shah AH, Benveniste R, Jimsheleishvili G, Lee SH, Loewenstein D, et al. Comparing invasive with MRI-derived intracranial pressure measurements in healthy elderly and brain trauma cases: A pilot study: Comparing LPOP and MR-ICP in Controls. J Magn Reson Imaging. 2019 Sep;50(3):975–81. doi: [10.1002/jmri.26695](https://doi.org/10.1002/jmri.26695)

149. Pomschar A, Koerte I, Lee S, Laubender RP, Straube A, Heinen F, et al. MRI Evidence for Altered Venous Drainage and Intracranial Compliance in Mild Traumatic Brain Injury. PLoS ONE. 2013 Feb 6;8(2):1–9. doi: [10.1371/journal.pone.0055447](https://doi.org/10.1371/journal.pone.0055447)

150. Frigieri G, Andrade RAP, Dias C, Spavieri DL, Brunelli R, Cardim DA, et al. Analysis of a Non-invasive Intracranial Pressure Monitoring Method in Patients with Traumatic Brain Injury. Acta Neurochirurgica Supplement. 2018;126:107–10. doi: [10.1007/978-3-319-65798-1_23](https://doi.org/10.1007/978-3-319-65798-1_23)

151. Kostopoulos V, Loutas TH, Derdas C, Douzinas E. Wavelet Analysis of Head Acceleration Response Under Dirac Excitation for Early Oedema Detection. Journal of Biomechanical Engineering. 2008 Apr 1;130(2):1–8. doi: [10.1115/1.2903432](https://doi.org/10.1115/1.2903432)

152. Asgari S, Adams H, Kasprowicz M, Czosnyka M, Smielewski P, Ercole A. Feasibility of Hidden Markov Models for the Description of Time-Varying Physiologic State After Severe Traumatic Brain Injury. Critical Care Medicine. 2019 Nov;47(11):e880–5. doi: [10.1097/CCM.0000000000003966](https://doi.org/10.1097/ccm.0000000000003966)

153. Bentsen G, Stubhaug A, Eide PK. Differential effects of osmotherapy on static and pulsatile intracranial pressure*. Critical Care Medicine. 2008 Aug;36(8):2414–9. doi: [10.1097/CCM.0b013e318180fe04](https://doi.org/10.1097/ccm.0b013e318180fe04)

154. Eide PK, Rapoport BI, Gormley WB, Madsen JR. A dynamic nonlinear relationship between the static and pulsatile components of intracranial pressure in patients with subarachnoid hemorrhage. Clinical article. JNS. 2010 Mar;112(3):616–25. doi: [10.3171/2009.7.JNS081593](https://doi.org/10.3171/2009.7.jns081593)

155. Eide PK, Sorteberg W. Intracranial pressure levels and single wave amplitudes, Glasgow Coma Score and Glasgow Outcome Score after subarachnoid haemorrhage. Acta Neurochir (Wien). 2006 Dec;148(12):1267–76. doi: [10.1007/s00701-006-0908-0](https://doi.org/10.1007/s00701-006-0908-0)

156. Eide PK, Sorteberg W. Association among intracranial compliance, intracranial pulse pressure amplitude and intracranial pressure in patients with intracranial bleeds. Neurological Research. 2007 Dec;29(8):798–802. doi: [10.1179/016164107X224132](https://doi.org/10.1179/016164107x224132)

157. Ng SCP, Poon WS, Chan MTV. Cerebral haemodynamic assessment in patients with thalamic haemorrhage: a pilot study with continuous compliance monitoring. Acta Neurochir. 2005;95:299–301. doi: [10.1007/3-211-32318-x_61](https://doi.org/10.1007/3-211-32318-x_61)

158. Eide PK, Bentsen G, Stanisic M, Stubhaug A. Association between intracranial pulse pressure levels and brain energy metabolism in a patient with an aneurysmal subarachnoid haemorrhage. Acta Anaesthesiol Scand. 2007 Aug;51:1273–6. doi: [10.1111/j.1399-6576.2007.01433.x](https://doi.org/10.1111/j.1399-6576.2007.01433.x)

159. Cardoso ER, Reddy K, Bose D. Effect of subarachnoid hemorrhage on intracranial pulse waves in cats. Journal of Neurosurgery. 1988 Nov;69(5):712–8. doi: [10.3171/jns.1988.69.5.0712](https://doi.org/10.3171/jns.1988.69.5.0712)

160. Bentsen G. ICP Versus Intracranial Compliance (ICC) Guided Management in SAH. ClinicalTrials.gov. 2011 Jan. Available from https://clinicaltrials.gov/ct2/show/NCT00248690

161. Taylor GA. Sonographic assessment of posthemorrhagic ventricular dilatation. Radiologic Clinics of North America. 2001 May;39(3):541–51. doi: [10.1016/s0033-8389(05)70296-7](https://doi.org/10.1016/s0033-8389(05)70296-7)

162. Adams RE, Powers WJ. Management of hypertension in acute intracerebral hemorrhage. Critical Care Clinics. 1997 Jan;13(1):131–61. doi: [10.1016/s0749-0704(05)70299-2](https://doi.org/10.1016/s0749-0704(05)70299-2)

163. Diringer MN. Intracerebral hemorrhage: Pathophysiology and management. Neurologic Critical Care. 1993;21(10):1591–1603. doi: [10.1097/00003246-199310000-00032](https://doi.org/10.1097/00003246-199310000-00032)

164. Horcajadas Almansa A, Cordero Tous N, Román Cutillas A, Saura Rojas E, Jorques Infante A, Iáñez Velasco B, et al. Utilidad de la monitorización de la presión intracraneal en pacientes con sospecha de hipertensión intracraneal benigna. Neurocirugía. 2015 Jul;26(4):157–66. doi: [10.1016/j.neucir.2014.11.010](https://doi.org/10.1016/j.neucir.2014.11.010)

165. Chisholm JT, Sudhakar P, Alhajeri AN, Smith JH. Intracranial elastance is increased in idiopathic intracranial hypertension. Eur J Neurol. 2017 Dec;24(12):1457–63. doi: [10.1111/ene.13410](https://doi.org/10.1111/ene.13410)

166. De Simone R, Ranieri A, Sansone M, Marano E, Russo CV, Saccà F, et al. Dural sinus collapsibility, idiopathic intracranial hypertension, and the pathogenesis of chronic migraine. Neurol Sci. 2019 May;40(S1):59–70. doi: [10.1007/s10072-019-03775-w](https://doi.org/10.1007/s10072-019-03775-w)

167. Eide PK, Eidsvaag VA, Nagelhus EA, Hansson H-A. Cortical astrogliosis and increased perivascular aquaporin-4 in idiopathic intracranial hypertension. Brain Research. 2016 Aug;1644:161–75. doi: [10.1016/j.brainres.2016.05.024](https://doi.org/10.1016/j.brainres.2016.05.024)

168. Griffith B, Capobres T, Patel SC, Marin H, Katramados A, Poisson LM. CSF Pressure Change in Relation to Opening Pressure and CSF Volume Removed. AJNR Am J Neuroradiol. 2018 Jun;39(6):1185–90. doi: [10.3174/ajnr.A5642](http://dx.doi.org/10.3174/ajnr.A5642)

169. Hoffman KR, Chan SW, Hughes AR, Halcrow SJ. Management of Cerebellar Tonsillar Herniation following Lumbar Puncture in Idiopathic Intracranial Hypertension. Case Reports in Critical Care. 2015;2015:1–4. doi: [10.1155/2015/895035](https://dx.doi.org/10.1155%2F2015%2F895035)

170. Lam MF, Stokes BAR, Lind CRP. Raised intracranial pressure with upright posture. Journal of Clinical Neuroscience. 2011 Jul;18(7):988–9. doi: [10.1016/j.jocn.2010.11.024](https://doi.org/10.1016/j.jocn.2010.11.024)

171. Salman M. Why does tonsillar herniation not occur in idiopathic intracranial hypertension? Medical Hypotheses. 1999 Oct;53(4):270–1. doi: [10.1054/mehy.1998.0756](https://doi.org/10.1054/mehy.1998.0756)

172. Okon MD, Roberts CJ, Mahmoud AM, Springer AN, Small RH, McGregor JM, et al. Characteristics of the cerebrospinal fluid pressure waveform and craniospinal compliance in idiopathic intracranial hypertension subjects. Fluids Barriers CNS. 2018 Dec;15(1):21. doi: [10.1186/s12987-018-0106-5](https://doi.org/10.1186/s12987-018-0106-5)

173. Rasulo F, Piva S, Park S, Oddo M, Megjhani M, Cardim D, et al. The Association Between Peri-Hemorrhagic Metabolites and Cerebral Hemodynamics in Comatose Patients With Spontaneous Intracerebral Hemorrhage: An International Multicenter Pilot Study Analysis. Front Neurol. 2020 Oct 26;11:568536. doi: [10.3389/fneur.2020.568536](https://doi.org/10.3389/fneur.2020.568536)

174. Sahu S, Panda N, Swain A, Mathew P, Singla N, Gupta S, et al. Optic Nerve Sheath Diameter: Correlation With Intra-Ventricular Intracranial Measurements in Predicting Dysfunctional Intracranial Compliance. Cureus. 2021 Jan;13(1):1–9. doi: [10.7759/cureus.13008](https://doi.org/10.7759/cureus.13008)

175. Leech P, Miller JD. Intracranial volume-pressure relationships during experimental brain compression in primates: 2. Effect of induced changes in systemic arterial pressure and cerebral blood flow. Journal of Neurology, Neurosurgery & Psychiatry. 1974 Oct 1;37(10):1099–104. doi: 10.1136/jnnp.37.10.1099

176. Nornes H, Aaslid R, Lindegaard K-F. Intracranial pulse pressure dynamics in patients with intracranial hypertension. Acta neurochir. 1977 Sep;38(3–4):177–86. doi: [10.1007/BF01401089](https://doi.org/10.1007/bf01401089)

177. Piper IR, Chan KH, Whittle IR, Miller JD. An Experimental Study of Cerebrovascular Resistance, Pressure Transmission, and Craniospinal Compliance – Experimental Study. Neurosurgery. 1993;32(5):1992–8. doi: [10.1227/00006123-199305000-00014](https://doi.org/10.1227/00006123-199305000-00014)

178. Tain R-W, Bagci AM, Lam BL, Sklar EM, Ertl-Wagner B, Alperin N. Determination of cranio-spinal canal compliance distribution by MRI: Methodology and early application in idiopathic intracranial hypertension. J Magn Reson Imaging. 2011 Dec;34(6):1397–404. doi: [10.1002/jmri.22799](https://doi.org/10.1002/jmri.22799)

179. Gasparian SS, Serova NK, Sherbakova EYa, Belova TN. Compensatory Mechanisms in Patients with Benign Intracranial Hypertension Syndrome. Acta Neurochirurgica Supplement. 2002;81:31–3. doi: [10.1007/978-3-7091-6738-0_8](https://doi.org/10.1007/978-3-7091-6738-0_8)

180. Schummer W, Schummer C, Niesen W-D. Unrecognized Internal Jugular Vein Obstruction: Cause of Fatal Intracranial Hypertension After Tracheostomy?: Journal of Neurosurgical Anesthesiology. 2002 Oct;14(4):313–5. doi: [10.1097/00008506-200210000-00008](https://doi.org/10.1097/00008506-200210000-00008)

181. Shakhnovich AR, Shakhnovich VA, Galushkina AA. Noninvasive Assessment of the Elastance and Reserve Capacity of the Craniovertebral Contents Via Flow Velocity Measurements in the Straight Sinus by TCD During Body Tilting Test. Journal of Neuroimaging. 1999 Jul;9(3):141–9. doi: [10.1111/jon199993141](https://doi.org/10.1111/jon199993141)

182. Chernov M, Kamikawa S, Toledo R, Yamane F, Izawa M, Hayashi M, et al. Minimally Invasive Management of the Third Ventricle Glioma in a Patient without Hydrocephalus: Neurofiberscopic Biopsy Followed by Gamma Knife Radiosurgery. Minim Invasive Neurosurg. 2004 Aug;47(4):238–41. doi: [10.1055/s-2004-818495](https://doi.org/10.1055/s-2004-818495)

183. Irgau I. Elective Intraoperative Intracranial Pressure Monitoring During Laparoscopic Cholecystectomy. Arch Surg. 1995 Sep 1;130(9):1011–13. doi: [10.1001/archsurg.1995.01430090097028](https://doi.org/10.1001/archsurg.1995.01430090097028)

184. Bedford RF, Dacey R, Winn HR, Lynch C. Adverse impact of a calcium entry-blocker (verapamil) on intracranial pressure in patients with brain tumors. Journal of Neurosurgery. 1983 Nov;59(5):800–2. doi: [10.3171/jns.1983.59.5.0800](https://doi.org/10.3171/jns.1983.59.5.0800)

185. Paltsev EI, Sirovsky EB. Intracranial physiology and biomechanics: Clinical data on pressure-volume relationships and their interpretation. Journal of Neurosurgery. 1982 Oct;57(4):500–10. doi: [10.3171/jns.1982.57.4.0500](https://doi.org/10.3171/jns.1982.57.4.0500)

186. Moyse E, Ros M, Marhar F, Swider P, Schmidt EA. Characterization of Supra and Infratentorial ICP Profiles. Acta Neurochirurgica Supplement. 2016; 122:37–40. doi: [10.1007/978-3-319-22533-3_7](https://doi.org/10.1007/978-3-319-22533-3_7)

187. Sekhon MS, Griesdale DE, Ainslie PN, Gooderham P, Foster D, Czosnyka M, et al. Intracranial pressure and compliance in hypoxic ischemic brain injury patients after cardiac arrest. Resuscitation. 2019 Aug;141:96–103. doi: [10.1016/j.resuscitation.2019.05.036](https://doi.org/10.1016/j.resuscitation.2019.05.036)

188. Bharadwaj S, Venkatraghavan L. Beware of Changes in Intracranial Compliance During Cranioplasty. Journal of Neurosurgical Anesthesiology. 2015 Oct;27(4):350–1. doi: [10.1097/ANA.0000000000000155](https://doi.org/10.1097/ana.0000000000000155)

189. Kataria S, Rakesh SV, Panda NB, Bhagat H, Mukherjee KK, Yadav TD. Effect of pneumoperitoneum on intracranial pressure during supra- tentorial craniotomy : a case report. Acta Anaesth. Belg. 2010;61:217–20. PMID: 21388082

190. Solheim O, Vik A, Gulati S, Eide PK. Rapid and severe rise in static and pulsatile intracranial pressures during a generalized epileptic seizure. Seizure. 2008 Dec;17(8):740–3. doi: [10.1016/j.seizure.2008.05.006](https://doi.org/10.1016/j.seizure.2008.05.006)

191. Dyson EW, Chari A, Toma AK, Thorne LW, Watkins LD. Failed Foramen Magnum Decompression in Chiari I Malformation Is Associated With Failure to Restore Normal Intracranial Compliance: An Observational Cohort Study. Neurosurgery. 2020 Jun 1;86(6):E552–7. doi: [10.1093/neuros/nyaa079](https://doi.org/10.1093/neuros/nyaa079)

192. Frič R, Eide PK. Comparative observational study on the clinical presentation, intracranial volume measurements, and intracranial pressure scores in patients with either Chiari malformation Type I or idiopathic intracranial hypertension. JNS. 2017 Apr;126(4):1312–22. doi: [10.3171/2016.4.JNS152862](https://doi.org/10.3171/2016.4.jns152862)

193. Alperin N, Loftus JR, Oliu CJ, Bagci AM, Lee SH, Ertl-Wagner B, et al. Imaging-Based Features of Headaches in Chiari Malformation Type I. Neurosurgery. 2015 Jul 1;77(1):96–103. doi: [10.1227/NEU.0000000000000740](https://doi.org/10.1227/neu.0000000000000740)

194. Alperin N, Loftus JR, Oliu CJ, Bagci AM, Lee SH, Ertl-Wagner B, et al. Magnetic Resonance Imaging Measures of Posterior Cranial Fossa Morphology and Cerebrospinal Fluid Physiology in Chiari Malformation Type I. Neurosurgery. 2014 Nov 1;75(5):515–22. doi: [10.1227/NEU.0000000000000507](https://doi.org/10.1227/neu.0000000000000507)

195. Alperin N, Sivaramakrishnan A, Lichtor T. Magnetic resonance imaging—based measurements of cerebrospinal fluid and blood flow as indicators of intracranial compliance in patients with Chiari malformation. Journal of Neurosurgery. 2005 Jul;103(1):46–52. doi: [10.3171/jns.2005.103.1.0046](https://doi.org/10.3171/jns.2005.103.1.0046)

196. Frič R, Eide PK. Comparison of pulsatile and static pressures within the intracranial and lumbar compartments in patients with Chiari malformation type 1: a prospective observational study. Acta Neurochir. 2015;157:1411–23. doi: [10.1007/s00701-015-2465-x](https://doi.org/10.1007/s00701-015-2465-x)

197. Frič R, Lindstrøm EK, Ringstad GA, Mardal K-A, Eide PK. The association between the pulse pressure gradient at the cranio-cervical junction derived from phase-contrast magnetic resonance imaging and invasively measured pulsatile intracranial pressure in symptomatic patients with Chiari malformation type 1. Acta Neurochir. 2016 Dec;158(12):2295–304. doi: 10.1007/s00701-016-2979-x

198. Sivaramakrishnan A, Alperin N, Surapaneni S, Lichtor T. Evaluating the Effect of Decompression Surgery on Cerebrospinal Fluid Flow and Intracranial Compliance in Patients with Chiari Malformation with Magnetic Resonance Imaging Flow Studies. Neurosurgery. 2004 Dec 1;55(6):1344–51. doi: [10.1227/01.neu.0000143612.60114.2d](https://doi.org/10.1227/01.neu.0000143612.60114.2d)

199. Shaffer N, Martin B, Loth F. Cerebrospinal fluid hydrodynamics in type I Chiari malformation. Neurological Research. 2011 Apr;33(3):247–60. doi: [10.1179/016164111X12962202723805](https://doi.org/10.1179/016164111x12962202723805)

200. Tsai Y-H, Chen H-C, Tung H, Wu Y-Y, Chen H-M, Pan K-J, et al. Noninvasive assessment of intracranial elastance and pressure in spontaneous intracranial hypotension by MRI: MR Intracranial Pressure for SIH. J Magn Reson Imaging. 2018 Nov;48(5):1255–63. doi: [10.1002/jmri.25976](https://doi.org/10.1002/jmri.25976)

201. Ursino M, Di Giammarco P. A mathematical model of the relationship between cerebral blood volume and intracranial pressure changes: The generation of plateau waves. Ann Biomed Eng. 1991 Jan;19(1):15–42. doi: [10.1007/BF02368459](https://doi.org/10.1007/BF02368459)

202. Azevedo Filho HRC de, Adams CBT, Kerr J. Intracranial compliance during the post-operative period after surgery for intracranial aneurysms. Arq Neuro-Psiquiatr. 1979 Sep;37(3):250–4. doi: [10.1590/S0004-282X1979000300003](https://doi.org/10.1590/S0004-282X1979000300003)

203. Chernov MF, Kamikawa S, Yamane F, Ishihara S, Hori T. Neurofiberscope-guided management of slit-ventricle syndrome due to shunt placement. Journal of Neurosurgery: Pediatrics. 2005 Apr;102(3):260–7. doi: [10.3171/ped.2005.102.3.0260](https://doi.org/10.3171/ped.2005.102.3.0260)

204. Bateman GA. Hypertensive slit ventricle syndrome: pseudotumor cerebri with a malfunctioning shunt?: Report of 3 cases. JNS. 2013 Dec;119(6):1503–10. doi: [10.3171/2013.7.JNS13390](https://doi.org/10.3171/2013.7.jns13390)

205. Lavinio A, Rasulo FA, De Peri E, Czosnyka M, Latronico N. The relationship between the intracranial pressure–volume index and cerebral autoregulation. Intensive Care Med. 2009 Mar;35(3):546–9. doi: [10.1007/s00134-008-1311-5](https://doi.org/10.1007/s00134-008-1311-5)

206. König K, Heissler HE, Zumkeller M, Rickels E. Age-dependence of cerebrospinal parameters. Acta Neurochirurgica Supplementum, 2005;95:315–8. doi: [10.1007/3-211-32318-x_65](https://doi.org/10.1007/3-211-32318-x_65)

207. Svedung Wettervik T, Kumlien E, Rostami E, Howells T, von Seth M, Velickaite V, et al. Intracranial Pressure Dynamics and Cerebral Vasomotor Reactivity in Coronavirus Disease 2019 Patient With Acute Encephalitis. Critical Care Explorations. 2020 Aug;2(8):e0197. doi: [10.1097/CCE.0000000000000197](https://doi.org/10.1097/cce.0000000000000197)

208. Brasil S. Cerebral Compliance Impairment in COVID-19. ClinicalTrials.gov. 2020 Sep. Available from: https://clinicaltrials.gov/ct2/show/NCT04429477

209. Aye T. Evaluation of a Non-invasive Brain Compliance Measurement Device. ClinicalTrials.gov. 2019 Nov. Available from: https://clinicaltrials.gov/ct2/show/NCT01753921

210. De laet I, Citerio G, Malbrain MLNG. The influence of intra-abdominal hypertension on the central nervous system: current insights and clinical recommendations, is it all in the head? Acta Clinica Belgica. 2007 Jan;62(sup1):89–97. doi: [10.1179/acb.2007.62.s1.012](http://dx.doi.org/10.1179/acb.2007.62.s1.012)

211. Kumar A, Cage A, Dhar R. Dialysis-Induced Worsening of Cerebral Edema in Intracranial Hemorrhage: A Case Series and Clinical Perspective. Neurocrit Care. 2015 Apr;22(2):283–7. [10.1007/s12028-014-0063-z](https://doi.org/10.1007/s12028-014-0063-z)

212. Rajagopalan S, Cruz Navarro J, Baghshomali S, Kirschen M, Greer D, Kofke WA, et al. Physiological Signatures of Brain Death Uncovered by Intracranial Multimodal Neuromonitoring. Journal of Neurosurgical Anesthesiology. 2019 Dec 23:1–4. doi: [10.1097/ANA.0000000000000672](https://doi.org/10.1097/ana.0000000000000672)

213. Sood S, Kumar CR, Jamous M, Schuhmann MU, Ham SD, Canady AI. Pathophysiological changes in cerebrovascular distensibility in patients undergoing chronic shunt therapy. Journal of Neurosurgery: Pediatrics. 2004 May;100(5):447–53. doi: [10.3171/ped.2004.100.5.0447](https://doi.org/10.3171/ped.2004.100.5.0447)

214. Lim ST, Potts DG, Deonarine V, Deck MDF. Ventricular compliance in dogs with and without aqueductal obstruction. Journal of Neurosurgery. 1973 Oct;39(4):463–73. doi: [10.3171/jns.1973.39.4.0463](https://doi.org/10.3171/jns.1973.39.4.0463)

215. Gilland O. CSF dynamic diagnosis of spinal block II. Acta Neurologica Scandinavica. 1965 Dec;41(5):487–96. doi: [10.1111/j.1600-0404.1965.tb04739.x](https://doi.org/10.1111/j.1600-0404.1965.tb04739.x)

216. Löfgren J, Zwetnow NN. Cranial and spinal components of the cerebrospinal fluid pressure-volume curve. Acta Neurologica Scandinavica. 2009 Jan 29;49(5):575–85. doi: [10.1111/j.1600-0404.1973.tb01331.x](https://doi.org/10.1111/j.1600-0404.1973.tb01331.x)

217. Doyle DJ, Mark PWS. Analysis of intracranial pressure. Journal of Clinical Monitoring. 1992;8(1):81–90. doi: [10.1007/BF01618093](https://doi.org/10.1007/bf01618093)

218. Germon K. Intracranial pressure monitoring in the 1990s. Critical Care Nursing Quarterly. 1994 May;17(1):21–32. doi: [10.1097/00002727-199405000-00004](https://doi.org/10.1097/00002727-199405000-00004)

219. Germon K. Interpretation of ICP pulse waves to determine intracerebral compliance. Journal of Neuroscience Nursing. 1988 Dec;20(6):344–350. doi: [10.1097/01376517-198812000-00004](https://doi.org/10.1097/01376517-198812000-00004)

220. Hickey JV, Olson DM, Turner DA. Intracranial Pressure Waveform Analysis During Rest and Suctioning. Biological Research For Nursing. 2009 Oct;11(2):174–86. doi: [10.1177/1099800409332902](https://doi.org/10.1177/1099800409332902)

221. Lai H-Y, Lee C-H, Lee C-Y. The Intracranial Volume Pressure Response in Increased Intracranial Pressure Patients: Clinical Significance of the Volume Pressure Indicator. Boltze J, editor. PLoS ONE. 2016 Oct 10;11(10):e0164263. doi: [10.1371/journal.pone.0164263](https://dx.doi.org/10.1371%2Fjournal.pone.0164263)

222. Latka M, Kolodziej W, Turalska M, Latka D, Zub W, West BJ. Wavelet assessment of cerebrospinal compensatory reserve and cerebrovascular reactivity. Physiol Meas. 2007 May 1;28(5):465–79. doi: [10.1088/0967-3334/28/5/002](https://doi.org/10.1088/0967-3334/28/5/002)

223. Marshall SA, Kalanuria A, Markandaya M, Nyquist PA. Management of Intracerebral Pressure in the Neurosciences Critical Care Unit. Neurosurgery Clinics of North America. 2013 Jul;24(3):361–73. doi: [10.1016/j.nec.2013.03.004](https://doi.org/10.1016/j.nec.2013.03.004)

224. Ross N, Eynon CA. Intracranial pressure monitoring. Current Anaesthesia & Critical Care. 2005 Jan;16(4):255–61. doi: [10.1016/j.cacc.2005.11.013](https://doi.org/10.1016/j.cacc.2005.11.013)

225. Schaller B, Graf R. Different Compartments of Intracranial Pressure and Its Relationship to Cerebral Blood Flow: The Journal of Trauma: Injury, Infection, and Critical Care. 2005 Dec;59(6):1521–31. doi: [10.1097/01.ta.0000197402.20180.6b](https://doi.org/10.1097/01.ta.0000197402.20180.6b)

226. Wilkinson HA, Schuman N, Ruggiero J. Nonvolumetric methods of detecting impaired intracranial compliance or reactivity: Pulse width and wave form analysis. Journal of Neurosurgery. 1979 Jun;50(6):758–67. doi: 10.3171/jns.1979.50.6.0758

227. Tirado-Caballero J, Rivero-Garvia M, Moreno-Madueño G, Gómez-González E, Márquez-Rivas J. Cranial expansion and aqueductoplasty for combined isolated fourth ventricle and slit-ventricle syndrome: a surgical alternative. Childs Nerv Syst. 2021 Mar;37(3):885–94. doi: [10.1007/s00381-020-04939-2](https://doi.org/10.1007/s00381-020-04939-2)

228. Shapiro K, Marmarou A, Shulman K. Characterization of clinical CSF dynamics and neural axis compliance using the pressure-volume index: I. The normal pressure-volume index. Ann Neurol. 1980 Jun;7(6):508–14. doi: [10.1002/ana.410070603](https://doi.org/10.1002/ana.410070603)

229. Al-Mufti F, Sursal T, Kim M, Menjivar AM, Cole C, Chandy D, et al. Noninvasive Multimodality Cerebral Monitoring Modalities in Neurosurgical Critical Care. World Neurosurgery. 2019 Jan;121:249–50. doi: [10.1016/j.wneu.2018.10.076](https://doi.org/10.1016/j.wneu.2018.10.076)

230. Chan MTV, Lam JMK. New monitors of neurological functions - part 2. Current Anaesthesia and Critical Care. 1999;10:147–157. doi: [10.1016/S0953-7112(99)80007-6](https://doi.org/10.1016/S0953-7112(99)80007-6)

231. Czosnyka M, Lavinio A, Pickard JD. Intracranial Pressure: More Than a Number. Neurosurg Focus. 2007;22(5):1–7. doi: [10.3171/foc.2007.22.5.11](https://doi.org/10.3171/foc.2007.22.5.11)

232. Kocaeli H, Korfalı E, Taşkapılıoğlu Ö, Özcan T. Analysis of intracranial pressure changes during early versus late percutaneous tracheostomy in a neuro-intensive care unit. Acta Neurochir (Wien). 2008 Dec;150(12):1263–7. doi: [10.1007/s00701-008-0153-9](https://doi.org/10.1007/s00701-008-0153-9)

233. Schneider G-H, Helden A, Franke R, Lanksch WR, Unterberg A. Influence of Body Position on Jugular Venous Oxygen Saturation, Intracranial Pressure and Cerebral Perfusion Pressure. Acta Neutochirurgica Supplements. 1993; 59:107–12. doi: [10.1007/978-3-7091-9302-0_19](https://doi.org/10.1007/978-3-7091-9302-0_19)

234. Wright BD, Young B. Automatic intracranial pressure regulation. Critical Care Medicine. 1978;6(6):373–5. doi: [10.1097/00003246-197811000-00006](https://doi.org/10.1097/00003246-197811000-00006)

235. Gonzalez MA, Quiles MA, Pulido N, Garcia-Sola R, Salvador CH. Intracranial pressure dynamics in clinical practice: online PC-based ICP monitoring system. Medical & Biological Engineering & Computing. 1994;32:512–20. doi: [10.1007/BF02515309](https://doi.org/10.1007/bf02515309)

236. Lazaridis C, Czosnyka M. Cerebral Blood Flow, Brain Tissue Oxygen, and Metabolic Effects of Decompressive Craniectomy. Neurocrit Care. 2012 Jun;16(3):478–84. doi: [10.1007/s12028-012-9685-1](https://doi.org/10.1007/s12028-012-9685-1)

237. Unnerbäck M, Bloomfield EL, Söderström S, Reinstrup P. The intracranial pressure curve correlates to the pulsatile component of cerebral blood flow. J Clin Monit Comput. 2019 Feb;33(1):77–83. doi: [10.1007/s10877-018-0129-0](https://doi.org/10.1007/s10877-018-0129-0)

238. Yau Y, Piper I, Contant C, Citerio G, Kiening K, Enblad P, et al. Multi-Centre Assessment of the Spiegelberg Compliance Monitor: Interim Results. Acta Neurochirurgica Supplements, 2002;81:167–70. doi: [10.1007/978-3-7091-6738-0_43](https://doi.org/10.1007/978-3-7091-6738-0_43)

239. Norager NH, Olsen MH, Riedel CS, Juhler M. Changes in intracranial pressure and pulse wave amplitude during postural shifts. Acta Neurochir. 2020 Dec;162(12):2983–9. doi: [10.1007/s00701-020-04550-z](https://doi.org/10.1007/s00701-020-04550-z)

240. Eide PK, Egge A, Due-Tønnessen BJ, Helseth E. Is Intracranial Pressure Waveform Analysis Useful in the Management of Pediatric Neurosurgical Patients? Pediatr Neurosurg. 2007;43(6):472–81. doi: [10.1159/000108790](https://doi.org/10.1159/000108790)

241. Piper O, Dunn L, Contant C, You Y, Whittle I, Citerio G, Kiening K, Shcvning W, et al. Multi-Centre Assessment of Spielberg COmpliance Monitor: Preliminary Results. Acta Neutochirurgica Supplements. 2000;76:491–4. doi: [10.1016/j.jvs.2020.12.092](https://doi.org/10.1016/j.jvs.2020.12.092)

242. Alperin N. MR–Intracranial Compliance and Pressure: A Method for Noninvasive Measurement of Important Neurophysiologic Parameters. Methods in Enzymology. 2004;386:323–49. doi: [10.1016/S0076-6879(04)86016-6](https://doi.org/10.1016/s0076-6879(04)86016-6)

243. Robba C, Cardim D, Sekhon M, Budohoski K, Czosnyka M. Transcranial Doppler: a stethoscope for the brain-neurocritical care use. J Neuro Res. 2018 Apr;96(4):720–30. doi: [10.1002/jnr.24148](https://doi.org/10.1002/jnr.24148)

244. Wagshul ME, Eide PK, Madsen JR. The pulsating brain: A review of experimental and clinical studies of intracranial pulsatility. Fluids Barriers CNS. 2011 Dec;8(1):5. doi: [10.1186/2045-8118-8-5](https://doi.org/10.1186/2045-8118-8-5)

245. Brasil S. A Novel Non-invasive Technique of Cerebral Compliance and Auto-regulation Assessment. ClinicalTrials.gov. 2020 Sep. Available from: https://clinicaltrials.gov/ct2/show/NCT03144219

246. Chambers IR, Daubaris G, Jarzemskas E, Fountas K, Kvascevicius R, Ragauskas A, et al. The clinical application of non-invasive intracranial blood volume pulse wave monitoring. Physiol Meas. 2005 Dec 1;26(6):1019–32. doi: [10.1088/0967-3334/26/6/011](https://doi.org/10.1088/0967-3334/26/6/011)

247. Spiegelberg A, Preuß M, Kurtcuoglu V. B-waves revisited. Interdisciplinary Neurosurgery. 2016 Dec;6:13–7. doi: [10.1016/j.inat.2016.03.004](https://doi.org/10.1016/j.inat.2016.03.004)

248. Alperin N, Mazda M, Lichtor T, Lee S. From Cerebrospinal Fluid Pulsation to Noninvasive Intracranial Compliance and Pressure Measured by MRI Flow Studies. CMIR. 2006 Feb 1;2(1):117–29. doi: [10.2174/157340506775541622](https://doi.org/10.2174/157340506775541622)

249. Ohara S, Nagai H, Matsumoto T, Banno T. MR imaging of CSF pulsatory flow and its relation to intracranial pressure. Journal of Neurosurgery. 1988 Nov;69(5):675–82. doi: [10.3171/jns.1988.69.5.0675](https://doi.org/10.3171/jns.1988.69.5.0675)

250. Ursino M, Giulioni M, Lodi CA. Relationships among cerebral perfusion pressure, autoregulation, and transcranial Doppler waveform: a modeling study. Journal of Neurosurgery. 1998 Aug;89(2):255–66. doi: [10.3171/jns.1998.89.2.0255](https://doi.org/10.3171/jns.1998.89.2.0255)

251. Schummer W, Schummer C, Niesen W-D, Gerstenberg H. Doppler-guided cannulation of internal jugular vein, subclavian vein and innominate (brachiocephalic) vein—a case-control comparison in patients with reduced and normal intracranial compliance. Intensive Care Med. 2003 Sep;29(9):1535–40. doi: 10.1007/s00134-003-1862-4

252. Baglio S. Signal processing methodologies of intracranial pressure measurements for the compliance characterization. Engineering in Medicine and Biology. 2002:118–9. doi: [10.1109/IEMBS.2002.1134414](https://doi.org/10.1109/IEMBS.2002.1134414)

253. Lodi CA, Ursino M, Minassian AT, Beydon L. A Mathematical Model of Intracranial Pressure and Cerebral hemodynamics Response to CO_2_ changes. Transactions on Biomedicine and Health. 1997;4:101–11. doi: 10.2495/BIO970101

254. Qiu L, Xu L, Wang Y. Modeling of the Interaction between Intracranial Pressure and Cerebral Blood Flow. Third International Conference on Biomedical Engineering and Informatics. 2010:1217–20. doi: [10.1109/BMEI.2010.5639292](https://doi.org/10.1109/BMEI.2010.5639292)

255. Czosnyka M, Batorski L, Roszkowski M, et al. Cerebrospinal compensation in hydrocephalic children. Child's Nerv Syst. 1993;9:17–22. doi: [10.1007/BF00301929](https://doi.org/10.1007/bf00301929)

256. Eide PK. Assessment of Childhood Intracranial Pressure Recordings Using a New Method of Processing Intracranial Pressure Signals. Pediatr Neurosurg. 2005;41(3):122–30. doi: [10.1159/000085868](https://doi.org/10.1159/000085868)

257. Schuhmann MU, Sood S, McAllister JP, Jaeger M, Ham SD, Czosnyka Z, et al. Value of Overnight Monitoring of Intracranial Pressure in Hydrocephalic Children. Pediatric Neurosurgery. 2008;44(4):269–79. doi: [10.1159/000131675](https://doi.org/10.1159/000131675)

258. Sæhle T, Eide PK. Intracranial pressure monitoring in pediatric and adult patients with hydrocephalus and tentative shunt failure: a single-center experience over 10 years in 146 patients. JNS. 2015 May;122(5):1076–86. doi: [10.3171/2014.12.JNS141029](https://doi.org/10.3171/2014.12.jns141029)

259. Sæhle T, Eide PK. Characteristics of intracranial pressure (ICP) waves and ICP in children with treatment-responsive hydrocephalus. Acta Neurochir. 2015 Jun;157(6):1003–14. doi: [10.1007/s00701-015-2410-z](https://doi.org/10.1007/s00701-015-2410-z)

260. Ahmad I, Wahab S, Chana RS, Khan RA, Wahab A. Role of Transcranial Doppler and Pressure Provocation in Evaluation of Cerebral Compliance in Children with Hydrocephalus. HK J Paediatr. 2008;13:110–15. Available from: http://hkjpaed.org/pdf/2008%3B13%3B110-115.pdf

261. Goh D, Minns RA, Pye SD, Steers AJW. Cerebral blood-flow velocity and intermittent intracranial pressure elevation during sleep in hydrocephalic children. Developmental Medicine & Child Neurology. 2008 Nov 12;34(8):676–89. doi: [10.1111/j.1469-8749.1992.tb11503.x](https://doi.org/10.1111/j.1469-8749.1992.tb11503.x)

262. Paraguassu G, Khilnani M, Rabelo NN, Cobos LD, Frigieri G. Case Report: Untreatable Headache in a Child With Ventriculoperitoneal Shunt Managed by Use of New Non-invasive Intracranial Pressure Waveform. Front Neurosci. 2021 Feb 10;15:1–4. doi: [10.3389/fnins.2021.601945](https://doi.org/10.3389/fnins.2021.601945)

263. Aboy M, McNames J, Wakeland W, Goldstein B. Pulse and mean intracranial pressure analysis in pediatric traumatic brain injury. Acta Neurochirurgica Supplementum. 2005;95:307–10. doi: [10.1007/3-211-32318-x_63](https://doi.org/10.1007/3-211-32318-x_63)

264. Fanelli A, Vonberg FW, LaRovere KL, Walsh BK, Smith ER, Robinson S, et al. Fully automated, real-time, calibration-free, continuous noninvasive estimation of intracranial pressure in children. Journal of Neurosurgery: Pediatrics. 2019 Nov;24(5):509–19. doi: [10.3171/2019.5.PEDS19178](https://doi.org/10.3171/2019.5.peds19178)

265. Wolf MS, Rakkar J, Horvat CM, Simon DW, Kochanek PM, Clermont G, et al. Assessment of Dynamic Intracranial Compliance in Children with Severe Traumatic Brain Injury: Proof-of-Concept. Neurocrit Care. 2021 Feb;34(1):209–17. doi: [10.1007/s12028-020-01004-3](https://dx.doi.org/10.1007%2Fs12028-020-01004-3)

266. Yikilmaz A, Taylor GA. Sonographic findings in bacterial meningitis in neonates and young infants. Pediatr Radiol. 2008 Feb;38(2):129–37. doi: [10.1007/s00247-007-0538-6](https://dx.doi.org/10.1007%2Fs00247-007-0538-6)

267. Meyer PG, Ducrocq S, Rackelbom T, Orliaguet G, Renier D, Carli P. Surgical evacuation of acute subdural hematoma improves cerebral hemodynamics in children: a transcranial Doppler evaluation. Childs Nerv Syst. 2005 Feb;21(2):133–7. doi: [10.1007/s00381-004-1016-1](https://doi.org/10.1007/s00381-004-1016-1)

268. Westra SJ, Stotland MA, Lazareff J, Anderson CTM, Sayre JW, Kawamoto H. Perioperative Transcranial Doppler US to Evaluate Intracranial Compliance in Young Children Undergoing Craniosynostosis Repair Surgery. Radiology. 2001 Mar;218(3):816–23. doi: [10.1148/radiology.218.3.r01mr36816](https://doi.org/10.1148/radiology.218.3.r01mr36816)

269. Zipfel J, Jager B, Collmann H, Czosnyka Z, Schuhmann MU, Schweitzer T. The role of ICP overnight monitoring (ONM) in children with suspected craniostenosis. Childs Nerv Syst. 2020 Jan;36(1):87–94. doi: [10.1007/s00381-019-04288-9](https://doi.org/10.1007/s00381-019-04288-9)

270. Taylor GA. Effect of Scanning Pressure on Intracranial Hemodynamics during Transfontanellar Duples US. Pediatric Radiology. 1992;185:763–6. doi: [10.1148/radiology.185.3.1438759](https://doi.org/10.1148/radiology.185.3.1438759)

271. Taylor GA, Phillips MD, Ichord RN, Carson BS, Gates JA, James CS. Intracranial Compliance in Infants: Evaluation with Doppler US. Pediatric Radiology. 1994;191:787–791. doi: [10.1148/radiology.191.3.8184065](https://doi.org/10.1148/radiology.191.3.8184065)

272. Uzzo RG, Bilsky M, Mininberg DT, Poppas DP. Laparoscopic surgery in children with ventriculoperitoneal shunts: Effect of pneumoperitoneum on intracranial pressure—preliminary experience. Urology. 1997 May;49(5):753–7. doi: [10.1016/S0090-4295(97)00233-1](https://doi.org/10.1016/s0090-4295(97)00233-1)

273. Johnson MJ, Ayzman I, Wood AS, Tkach JA, Klauschie J, Skarupa DJ, et al. Development and characterization of an adult model of obstructive hydrocephalus. Journal of Neuroscience Methods. 1999 Sep;91(1–2):55–65. doi: [10.1016/s0165-0270(99)00072-2](https://doi.org/10.1016/s0165-0270(99)00072-2)

274. Dombrowski SM, Schenk S, Leichliter A, Leibson Z, Fukamachi K, Luciano MG. Chronic Hydrocephalus-Induced Changes in Cerebral Blood Flow: Mediation through Cardiac Effects. J Cereb Blood Flow Metab. 2006 Oct;26(10):1298–310. doi: [10.1038/sj.jcbfm.9600282](https://doi.org/10.1038/sj.jcbfm.9600282)

275. Fukuhara T, Luciano MG, Brant CL, Klauscie J. Effects of ventriculoperitoneal shunt removal on cerebral oxygenation and brain compliance in chronic obstructive hydrocephalus. Journal of Neurosurgery. 2001 Apr;94(4):573–81. doi: [10.3171/jns.2001.94.4.0573](https://doi.org/10.3171/jns.2001.94.4.0573)

276. Brinker T, Beck H, Klinge P, Kischnik B, Oi S, Samii M. Sinusoidal Intrathecal Infusion for Assessment of CSF Dynamics in Kaolin-Induced Hydrocephalus. Acta Neurochirurgica. 1998 Oct 22;140(10):1069–75. doi: [10.1007/s007010050216](https://doi.org/10.1007/s007010050216)

277. Matsumoto T, Nagai H, Fukushima T, Mase M. Analysis of intracranial pressure pulse wave in experimental hydrocephalus. Child’s Nerv Syst. 1994 Mar;10(2):91–5. doi: [10.1007/BF00302770](https://doi.org/10.1007/bf00302770)

278. Castro ME, Portnoy HD, Maesaka J. Elevated Cortical Venous Pressure in Hydrocephalus. Neurosurgery. 1991;29(2):232–8. doi: [10.1097/00006123-199108000-00011](https://doi.org/10.1097/00006123-199108000-00011)

279. Xu H, Fame RM, Sadegh C, Sutin J, Naranjo C, Della Syau, et al. Choroid plexus NKCC1 mediates cerebrospinal fluid clearance during mouse early postnatal development. Nat Commun. 2021 Dec;12(1):447. doi: [10.1038/s41467-020-20666-3](https://doi.org/10.1038/s41467-020-20666-3)

280. Gu M, Kawoos U, McCarron R, Chavko M. Protection against Blast-Induced Traumatic Brain Injury by Increase in Brain Volume. BioMed Research International. 2017;2017:1–10. doi: [10.1155/2017/2075463](https://doi.org/10.1155/2017/2075463)

281. Hariri RJ. Traumatic brain injury, hemorrhagic shock, and fluid resuscitation: effects on intracranial pressure and brain compliance. J Neurosurg. 1993;79:421–7. doi: [10.3171/jns.1993.79.3.0421](https://doi.org/10.3171/jns.1993.79.3.0421)

282. Salci K, Enblad P, Piper I, Contant C, Nilsson P. A Model for Studies of Intracranial Volume Pressure Dynamics in Traumatic Brain Injury. Journal of Neurotrauma. 2004 Mar;21(3):317–27. doi: [10.1089/089771504322972103](https://doi.org/10.1089/089771504322972103)

283. Salci K, Nilsson P, Goiny M, Contant C, Piper I, Enblad P. Low Intracranial Compliance Increases the Impact of Intracranial Volume Insults to the Traumatized Brain. Neurosurgery. 2006 Aug 1;59(2):367–73. doi: [10.1227/01.NEU.0000222648.61065.38](https://doi.org/10.1227/01.NEU.0000222648.61065.38)

284. Smith DW, Bailes JE, Fisher JA, Robles J, Turner RC, Mills JD. Internal Jugular Vein Compression Mitigates Traumatic Axonal Injury in a Rat Model by Reducing the Intracranial Slosh Effect: Neurosurgery. 2012 Mar;70(3):740–6. doi: [10.1227/NEU.0b013e318235b991](https://doi.org/10.1227/neu.0b013e318235b991)

285. Williamson MR, Wilkinson CM, Dietrich K, Colbourne F. Acetazolamide Mitigates Intracranial Pressure Spikes Without Affecting Functional Outcome After Experimental Hemorrhagic Stroke. Transl Stroke Res. 2019 Aug;10(4):428–39. doi: [10.1007/s12975-018-0663-6](https://doi.org/10.1007/s12975-018-0663-6)

286. Puchstein C, Van Aken H, Anger C, Hidding J. Influence of urapidil on intracranial pressure and intracranial compliance in dogs. British Journal of Anaesthesia. 1983 May;55(5):443–8. doi: [10.1093/bja/55.5.443](https://doi.org/10.1093/bja/55.5.443)

287. Van Aken H, Puchstein C, Anger C, Heinecke A, Lawin P. Changes in Intracranial Pressure and Compliance during Adenosine Triphosphate-induced Hypotension in Dogs. Anesthesia & Analgesia. 1984 Apr;63(4):381–5. doi: [10.1213/00000539-198404000-00001](https://doi.org/10.1213/00000539-198404000-00001)

288. Van Aken H, Anger C, Puchstein C, Thijs J, Lawin P. Influence of ketanserin, and antihypertensive agent with specific 5-HT_2_-receptor blocking activity, on intracranial pressure. Critical Care Medicine. 1984 Jan;12(1):4–7. doi: [10.1097/00003246-198401000-00002](https://doi.org/10.1097/00003246-198401000-00002)

289. Douzinas EE, Kostopoulos V, Kypriades E, Pappas YZ, Lymberis A, Karmpaliotis DI, et al. Brain eigenfrequency shifting as a sensitive index of cerebral compliance in an experimental model of epidural hematoma in the rabbit: Preliminary study. Critical Care Medicine. 1999 May;27(5):978–84. doi: [10.1097/00003246-199905000-00040](https://doi.org/10.1097/00003246-199905000-00040)

290. Ramirez de Noriega F, Manley GT, Moscovici S, Itshayek E, Tamir I, Fellig Y, et al. A swine model of intracellular cerebral edema – Cerebral physiology and intracranial compliance. Journal of Clinical Neuroscience. 2018 Dec;58:192–9. doi: [10.1016/j.jocn.2018.10.051](https://doi.org/10.1016/j.jocn.2018.10.051)

291. Whittle IR, Piper IR, Miller JD. The contribution of arachidonic acid to the aetiology and pathophysiology of focal brain oedema; studies using an infusion oedema model. Acta neurochir. 1991 Mar;113(1–2):57–68. doi: [10.1007/BF01402116](https://doi.org/10.1007/bf01402116)

292. Whittle IR, Piper IR, Miller JD. The role of bradykinin in the etiology of vasogenic brain edema and perilesional brain dysfunction. Acta neurochir. 1992 Mar;115(1–2):53–9. doi: [10.1007/BF01400591](https://doi.org/10.1007/bf01400591)

293. Whittle IR, Ironside JW, Piper IR, Miller JD. Neuropathological and neurophysiological effects of interstitial white matter autologous and non-autologous protein containing solutions: Further evidence for a glioma derived permeability factor. Acta neurochir. 1993 Sep;120(3–4):164–74. doi: [10.1007/BF02112037](https://doi.org/10.1007/bf02112037)

294. Yang YB, Sun B, Yang Z, Wang J, Pong Y. Effects of acute hypoxia on intracranial dynamics in unanesthetized goats. Journal of Applied Physiology. 1993 May 1;74(5):2067–71. doi: [10.1152/jappl.1993.74.5.2067](https://doi.org/10.1152/jappl.1993.74.5.2067)

295. Ducey JP, Deppe SA, Foley KT. A Comparison of the Effects of Suxamethonium, Atracurium and Vecuronium on Intracranial Haemodynamics in Swine. Anaesth Intensive Care. 1989 Nov;17(4):448–55. doi: [10.1177/0310057X8901700409](https://doi.org/10.1177/0310057x8901700409)

296. Kotani J, Adachi R, Fujita N, Sugioka S, Ueda Y. Effect of Cerebral Venous Congestion on the Pressure-Volume Index in the Evaluation of Intracranial Pressure Dynamics. Journal of Neurosurgical Anesthesiology. 1993;5(2):121–6. doi: [10.1097/00008506-199304000-00009](https://doi.org/10.1097/00008506-199304000-00009)

297. Schettini A, Walsh EK. Contribution of brain distortion and displacement to CSF dynamics in experimental brain compression. American Journal of Physiology-Regulatory, Integrative and Comparative Physiology. 1991 Jan 1;260(1):R172–8. doi: [10.1152/ajpregu.1991.260.1.R172](https://doi.org/10.1152/ajpregu.1991.260.1.r172)

298. Purins K, Sedigh A, Molnar C, Jansson L, Korsgren O, Lorant T, et al. Standardized experimental brain death model for studies of intracranial dynamics, organ preservation, and organ transplantation in the pig*: Critical Care Medicine. 2011 Mar;39(3):512–7. doi: [10.1097/CCM.0b013e318206b824](https://doi.org/10.1097/ccm.0b013e318206b824)
